# Accelerating PROTACs Discovery Through a Direct‐to‐Biology Platform Enabled by Modular Photoclick Chemistry

**DOI:** 10.1002/advs.202400594

**Published:** 2024-04-30

**Authors:** Ke‐Nian Yan, Yong‐Qiang Nie, Jia‐Yu Wang, Guang‐Liang Yin, Qia Liu, Hao Hu, Xiaoxia Sun, Xiao‐Hua Chen

**Affiliations:** ^1^ State Key Laboratory of Drug Research Shanghai Institute of Materia Medica Chinese Academy of Sciences Shanghai 201203 China; ^2^ University of Chinese Academy of Sciences Beijing 100049 China; ^3^ Jiangxi Key Laboratory of Organic Chemistry Jiangxi Science and Technology Normal University Nanchang 330013 China; ^4^ School of Chinese Materia Medica Nanjing University of Chinese Medicine Nanjing 210023 China; ^5^ School of Pharmaceutical Science and Technology Hangzhou Institute for Advanced Study University of Chinese Academy of Sciences Hangzhou 310024 China

**Keywords:** High‐throughput chemical synthesis, modular synthesis, photoclick chemistry, PROTACs library, targeted protein degradation

## Abstract

Proteolysis targeting chimeras (PROTACs) have emerged as a promising strategy for drug discovery and exploring protein functions, offering a revolutionary therapeutic modality. Currently, the predominant approach to PROTACs discovery mainly relies on an empirical design–synthesis–evaluation process involving numerous cycles of labor‐intensive synthesis‐purification and bioassay data collection. Therefore, the development of innovative methods to expedite PROTAC synthesis and exploration of chemical space remains highly desired. Here, a direct‐to‐biology strategy is reported to streamline the synthesis of PROTAC libraries on plates, enabling the seamless transfer of reaction products to cell‐based bioassays without the need for additional purification. By integrating amide coupling and light‐induced primary amines and o‐nitrobenzyl alcohols cyclization (PANAC) photoclick chemistry into a plate‐based synthetic process, this strategy produces PROTAC libraries with high efficiency and structural diversity. Moreover, by employing this platform for PROTACs screening, we smoothly found potent PROTACs effectively inhibit triple‐negative breast cancer (TNBC) cell growth and induce rapid, selective targeted degradation of cyclin‐dependent kinase 9 (CDK9). The study introduces a versatile platform for assembling PROTACs on plates, followed by direct biological evaluation. This approach provides a promising opportunity for high‐throughput synthesis of PROTAC libraries, thereby enhancing the efficiency of exploring chemical space and accelerating the discovery of PROTACs.

## Graphical abstract

A direct‐to‐biology platform has been established to streamline proteolysis targeting chimeras (PROTACs) library synthesis in plate, via integrating amide coupling and light‐induced primary amines and o‐nitrobenzyl alcohols cyclization (PANAC) photoclick chemistry, allowing seamless transfer for immediate cell‐based evaluation. This approach enables rapid discovery of potent cyclin‐dependent kinase 9‐targeting PROTACs. The platform enhances efficiency in chemical space exploration, accelerating PROTAC discovery.

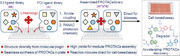

## 1 Introduction

Proteolysis targeting chimeras (PROTACs) are heterobifunctional molecules consisting of a target protein ligand, a connecting linker, and an E3 ligand, which recruit the target protein to E3 ubiquitin ligase for ubiquitination, subsequent degradation of the target protein via the ubiquitin‐proteasome system (UPS).^[^ ^1^, ^2^, ^3^, ^4^, ^5^ ^]^ PROTACs have exhibited notable advantages compared to traditional occupancy‐driven small molecule inhibitors, and the event‐driven protein degradation mechanism of PROTACs provides a unique capability to target a wide range of previously considered undruggable targets.^[^ ^6^, ^7^ ^]^ To date, more than 100 different proteins have been effectively targeted using PROTAC technology, and over 20 PROTAC molecules have advanced into clinical trials.^[^ ^2^, ^3^, ^8^ ^]^ Clearly, PROTAC technology provides a powerful tool for the degradation of disease‐causing proteins, representing a revolutionary strategy in drug discovery and opening a new therapeutic avenue for various diseases.^[^ ^2^, ^3^ ^]^ In addition, it is estimated that more than 1000 proteins within the human proteome may be considered amenable to PROTACs targeting. Consequently, the PROTACtable genome offers numerous opportunities for future efforts in PROTAC‐based degradation.^[^ ^9^, ^10^ ^]^

In comparison to traditional small‐molecule therapeutics, the heterobifunctional PROTACs possess more complex structures and relatively high molecular weights.^[^ ^2^, ^5^, ^7^ ^]^ The development of effective heterobifunctional PROTACs is primarily contingent on the attributes of the ligands within the protein of interest (POI)/ligase pairs, the composition of the linker, the site of linkage, and the length of the linker.^[^ ^8^, ^11^, ^12^, ^13^, ^14^, ^15^, ^16^ ^]^ As a result, the synthesis and optimization of PROTAC molecules present several challenges.^[^ ^8^, ^15^, ^16^, ^17^ ^]^ Currently, the design of PROTACs predominantly follows an empirical cycle of design‐synthesis‐evaluation, guided by multiple iterations of biological assay data obtained from the multi‐step synthesized PROTACs.^[^ ^15^, ^18^, ^19^ ^]^ Indeed, the synthesis of PROTACs is a time‐consuming and labor‐intensive process compared to smaller‐sized inhibitors. Hence, a central goal is to efficiently assemble ligands of POIs, linkers, and ligands of the ligase, with the aim to accelerate the exploration of the chemical space of PROTACs, thus, streamlining the design–synthesis–bioassay cycles.^[^ ^8^, ^15^, ^19^ ^]^ Moreover, it has become increasingly clear that the length and composition of the linker play pivotal roles in the ADME (absorption, distribution, metabolism, and excretion) properties of PROTACs,^[^ ^14^, ^15^, ^16^ ^]^ the formation of ternary complexes,^[^ ^8^, ^11^, ^12^, ^20^ ^]^ and the degradation of target proteins ^[^ ^13^, ^15^, ^21^ ^]^ Clearly, the advancement of efficient and versatile synthetic methods to access diverse linker structures would accelerate the identification of ideal PROTACs and enhance their bioactivity. Therefore, the development of advanced strategies to streamline the process of design–synthesis–evaluation for PROTACs and expand the chemical space available for degrader screening remains highly desired. Such advancements would speed up the optimization and discovery of PROTACs.^[^ ^8^, ^15^, ^21^, ^22^, ^23^ ^]^

Recently, the direct‐to‐biology strategy has been used for compound synthesis and screening, which integrates wellplate‐based high‐throughput compound synthesis with bioassays enabling streamlining time‐consuming steps in synthesis and bioactive molecules screening.^[^ ^24^, ^25^, ^26^, ^27^, ^28^, ^29^ ^]^ In contrast, the integration of plate‐based PROTACs synthesis approaches with direct bioassays remains largely underdeveloped. Despite the recent efforts that have introduced innovative strategies aimed at facilitating direct‐to‐biology approaches to expedite PROTAC synthesis and evaluation,^[^ ^30^, ^31^, ^32^, ^33^ ^]^ several challenges persist in the employed conjugation methods for PROTACs. These obstacles encompass issues such as unstable linkages,^[^ ^30^ ^]^ the persistent necessity for product purification,^[^ ^32^ ^]^ and the inclusion of coupling reagents in crude reaction mixtures may impede subsequent cellular bioassays.^[^ ^33^ ^]^ Moreover, the linker design in PROTACs is constrained to a single conjugation fashion, thereby limiting the exploration of the chemical space for PROTAC candidates.^[^ ^8^, ^14^, ^30^, ^31^, ^32^, ^33^ ^]^ Indeed, a primary synthetic challenge in developing a robust method for synthesizing a successful PROTAC library lies in the need for a conjugation strategy that provides sufficient structural diversity. This is crucial for exploring the chemical space of PROTAC candidates. More importantly, the reaction conditions employed in the PROTAC library synthesis process must be compatible with subsequent direct bioassays, thereby eliminating the necessity for the purification of reaction mixtures. Clearly, the seamless integration of a high‐throughput synthesis method for constructing a PROTAC library with subsequent direct bioassays remains a challenging and unmet requirement in the field. Such integration aims to streamline PROTAC synthesis and biological evaluation, thereby significantly facilitating the exploration of chemical space and accelerating the discovery of PROTACs.

**Figure 1:**
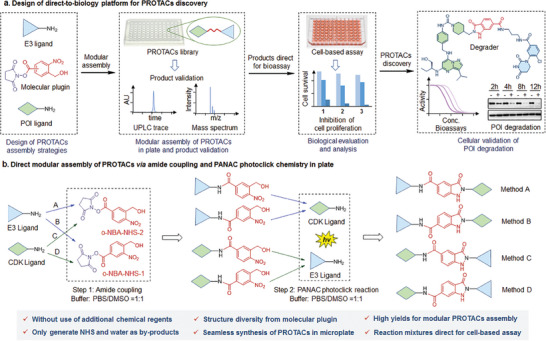
Design of direct‐to‐biology platform for plate‐based PROTAC library synthesis and subsequently direct for cell‐based evaluation. a). The workflow of a direct‐to‐biology platform based on the modular assembly of PROTAC library in plate, then subsequently direct for cell‐based evaluation, and further cellular validation of target degradation with identified degraders. b). The conjugation strategies for direct modular assembly of PROTACs through amide coupling via molecular plugin (o‐NBA‐NHS‐1 or o‐NBA‐NHS‐2), and PANAC photoclick chemistry in plate.

Herein, we have successfully established a direct‐to‐biology platform to efficiently streamline the synthesis of the PROTAC library on a plate, subsequently directly employed for cell‐based biological evaluations (**Figure** **1**a). By integrating amide coupling and light‐induced primary amines and o‐nitrobenzyl alcohols cyclization (PANAC) photoclick chemistry into a plate‐based synthetic process, this strategy enables the seamless transfer of crude reaction mixtures to cell‐based evaluation without the need for additional purification steps. Leveraging the flexibility in amide coupling sequences of primary amines derived from POI and E3 ligands via utilizing the molecular plugin o‐nitrobenzyl alcohol derived N‐hydroxysuccinimide‐esters (o‐NBA‐NHSs), then followed by PANAC photoclick reaction (Figure 1b), our approach rapidly produces 580 PROTAC candidates with high efficiency and structural diversity in short time. Moreover, by employing this direct‐to‐biology platform for PROTACs screening, coupled with the assessment of a few isolated PROTACs, we smoothly identified potent and selective cyclin‐dependent kinase 9 (CDK9) degraders that efficiently inhibit triple‐negative breast cancer (TNBC) cell growth and induce rapid, selective targeted degradation of CDK9, presenting a potential degrader for targeted therapy of TNBC. This study presents a promising and versatile platform for high‐throughput synthesis of PROTAC libraries on plate, thereby enhancing the efficiency of exploring chemical space and accelerating the discovery of PROTACs (Figure 1).

## 2 Results and Discussion

### 2.1 Designing a Direct‐to‐Biology Platform for Modular Assembly of PROTACs and Direct Cell‐Based Assay

Recently, the integration of miniaturized high‐throughput chemical synthesis approaches with biochemical assays has facilitated the rapid exploration of chemical space for small‐molecule hits and chemical probes, thereby accelerating the race in drug discovery.^[^ ^24^, ^25^, ^26^, ^27^, ^28^, ^29^ ^]^ Notably, in recent years, click chemistry has been employed for the construction of PROTACs and fine‐tuning the properties of the designed degraders.^[^ ^34^, ^35^ ^]^ However, this synthetic strategy was mainly used for a single experiment of PROTAC synthesis and still required further product purification processes to remove the metal catalyst and ligands.^[^ ^18^, ^36^, ^37^, ^38^ ^]^ We reasoned that several factors are required for the successful implementation of this direct‐to‐biology strategy: i) PROTACs are heterobifunctional molecules consisting of a target protein ligand, a connecting linker, and an E3 ligand, Therefore, the fragments for PROTAC library synthesis should be readily accessible; ii) The high‐throughput synthesis of PROTACs should be conducted without the use of toxic metal catalysts and additional chemical reagents or ligands, with the goal of minimizing toxicity to biological systems; iii) The by‐products generated during synthetic steps ideally should not interfere with subsequent bioassays; for instance, by‐products like water are considered acceptable; iv) The multi‐step synthesis reactions should be highly efficient and compatible in plates without the need for further purification steps, which ensures a seamless synthesis‐bioassay process; v) Due to the critical roles that linker structures (e.g., linkage site and linker length) play in the bioactivity of degraders,^[^ ^14^, ^15^, ^16^ ^]^ the linker should be stable and exhibit diversity in linkages.

Very recently, we developed the light‐induced primary amines and o‐nitrobenzyl alcohols cyclization (PANAC) photoclick reaction for the modular functionalization of diverse small molecules and native proteins,^[^ ^39^ ^]^ as well as covalent capture of protein–biomolecule interactions.^[^ ^40^, ^41^ ^]^ The PANAC photoclick reaction employs primary amines as direct click handles, where the o‐nitrobenzyl alcohol (o‐NBA) functionalities undergo conversion into aryl–nitroso intermediates upon light activation. The PANAC photoclick reaction selectively reacts with primary amines, exhibiting fast kinetics, exquisite chemoselectivity, and a wide scope in reactants.^[^ ^39^ ^]^ Moreover, the light‐induced PANAC reaction exhibits high efficiency even at low concentrations of reactants under operationally simple and mild conditions, requiring no toxic metal catalysts or ligands in vitro and in living systems.^[^ ^39^, ^40^, ^42^ ^]^ Furthermore, PANAC photoclick chemistry features a temporal‐controlled manner to drive chemical transformations, generating only two molecules of water as a by‐product. Based on these insights into PANAC photoclick chemistry,^[^ ^39^, ^40^, ^41^, ^42^ ^]^ we envisioned that the PANAC photoclick reaction would serve as an ideal chemistry for the modular assembly of PROTACs in a wellplate‐based synthetic process.

On the other hand, primary amines represent one of the most abundant functional groups, and primary‐amine‐containing molecules are prevalent and widespread in both synthetic chemistry and natural products.^[^ ^43^ ^]^ With this in mind, we reasoned that premodifying E3 ligands or POI ligands with primary amine functional groups would afford rapid access to a diverse array of E3 ligands and POI ligand fragments. Specifically, considering the extensive use and effectiveness of N‐hydroxysuccinimide‐esters (NHS‐esters) in bioconjugation reactions for efficiently forming amide bonds with amines, we recognized that the o‐NBA‐derived NHS‐esters (o‐NBA‐NHS‐1 or o‐NBA‐NHS‐2) could function as molecular plugins for amide coupling of primary amines derived from E3 ligands or POI ligands (Figure 1a,b). Thus, this concept affords the flexibility of o‐NBA‐NHS linkerology and amide coupling sequences of ligand‐derived primary amines for PROTAC assembly (Figure 1b), thereby expanding the chemical space available for PROTAC construction and facilitating a more in‐depth exploration of PROTAC screening and optimization.

In this context, we aimed to establish a direct‐to‐biology platform for PROTACs synthesis and evaluation by integrating o‐NBA‐derived NHS‐ester amide coupling with PANAC photoclick chemistry in a wellplate‐based synthetic process (Figure 1a). To be more specific, the two‐step modular assembly protocol involves o‐NBA‐NHS‐ester‐related amide coupling followed by the PANAC photoclick reaction, resulting in a PROTAC library (Figure 1b). This wellplate‐based PROTAC assembly allows for the direct addition of crude reaction mixtures to cell‐based bioassays without the need for purification (Figure 1a).

### 2.2 Establishing the Optimal Procedure for Plate‐Based Protacs Assembly

To investigate the efficiency and accessibility of the wellplate‐based synthesis protocol, which integrates o‐NBA‐derived NHS‐ester amide coupling and PANAC photoclick chemistry, we initially implemented a two‐step method for PROTAC synthesis in a 96‐well plate. Lenalidomide and its analogs stand out as the most prevalent and widely employed cereblon (CRBN) ligands in the development of PROTACs, employed to degrade targeted proteins across diverse domains of cancer research and treatment.^[^ ^2^, ^3^, ^4^ ^]^ In addition, selective degradation of the transcription regulator CDK9 via PROTACs represents a promising strategy and exhibits potential as a new targeted treatment option for various cancers.^[^ ^44^ ^]^ Thus, we synthesized the lenalidomide‐derived primary amine (E3‐L1) as the ligand fragment for the E3 ligase CRBN and the cyclin‐dependent kinase (CDK) inhibitor‐derived primary amine (POI‐L1) as ligand fragment for target protein CDK9,^[^ ^45^ ^]^ aiming to establish the procedure for plate‐based PROTACs assembly. The fragments E3‐L1, o‐NBA‐NHS‐1, and POI‐L1, were individually prepared as 100 mm stock solutions in DMSO. As the compounds of E3‐L1 and POI‐L1 were obtained in the form of hydrochloride salts, we used the basic phosphate buffer (20 mm PBS/DMSO = 1:1, pH 10.5) as the reaction buffer.

**Figure 2:**
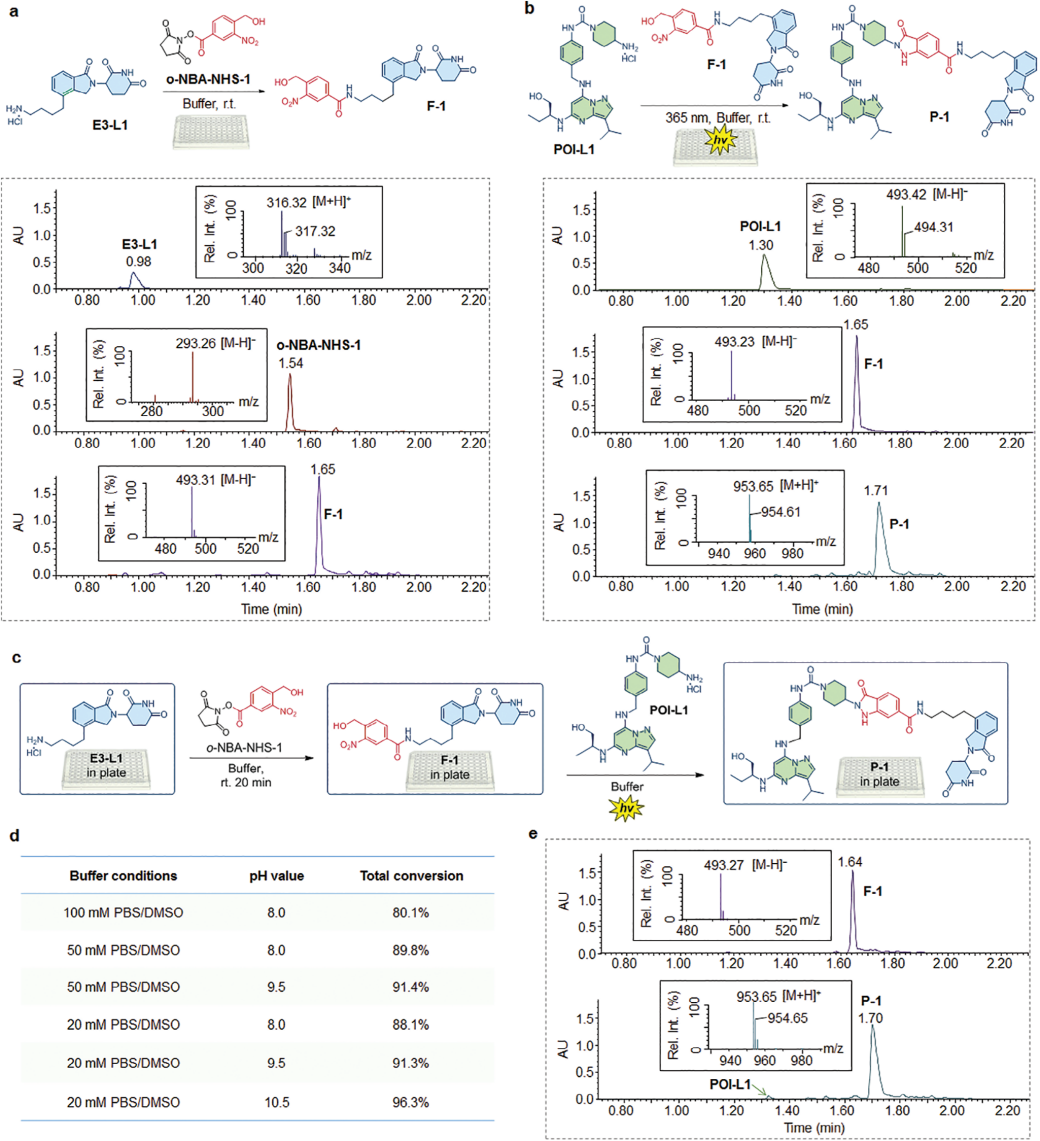
Establishing the procedure for plate‐based PROTAC assembly and validation of the assembly efficiency. a). Conjugation of E3 ligand (E3‐L1) with o‐NBA‐NHS‐1 in plate, and the UPLC trace and MS validation of the amide coupling efficiency, reaction conditions: E3‐L1 (4 mm) was incubated with o‐NBA‐NHS‐1 (4 mm) in reaction buffer (20 mm PBS/DMSO = 1:1, pH 10.5) for 20 min at room temperature (r.t.). b). PANAC photoclick conjugation of POI ligand (POI‐L1) with amide coupling product (F‐1) in plate, and the UPLC trace and MS validation of the PANAC photoclick efficiency, reaction conditions: mixture of POI‐L1 (2 mm) and F‐1 (2 mm) in reaction buffer (20 mm PBS/DMSO = 1:1, pH 10.5) was activated under 365 nm for 20 min at r.t. c). Schematic representation of the integration of amide coupling and PANAC photo click chemistry for the seamless synthesis of PROTAC **P‐1** in the plate. d) Screening of the reaction conditions for PROTAC **P‐1** assembly of (c) in plate, the total conversion of two‐step conjugation reactions, determined by the UPLC trace and MS analysis. e). UPLC trace and MS validation of the synthesis of PROTAC **P‐1** from (c) with buffer condition 20 mm PBS/DMSO, pH 10.5, in plate.

To evaluate the efficiency of the amide coupling between E3‐L1 and o‐NBA‐NHS‐1, a solution of E3‐L1 was mixed with an equal amount of o‐NBA‐NHS‐1, with the reactants in a basic phosphate reaction buffer. After shaking the reaction mixture at room temperature for 20 min, the reaction proceeded rapidly, yielding the desired product (F‐1) with nearly quantitative conversion (**Figure** **2**a), as determined by Ultra‐performance liquid chromatography (UPLC)–mass spectrometry (MS) analysis of the reaction mixture. In the subsequent step, we explored the potential of PANAC photoclick chemistry for PROTAC conjugation using an equimolar mixture of the POI‐L1 fragment and the amide coupling product F‐1 in a reaction buffer within a plate. The reaction mixture was directly subjected to light activation for 20 min on the plate. To our delight, the PANAC conjugation proceeded smoothly, resulting in over 95% conversion as determined by UPLC–MS analysis of reaction mixtures (Figure 2b and P‐1). Taken together, these results indicate that the o‐NBA‐NHS‐related amide coupling and PANAC photoclick chemistry are both suitable and highly efficient for the assembly of heterobifunctional molecules through the plate‐based protocol.

After obtaining these initial results, we conducted a survey of the reaction buffer to enable a seamless synthesis process for PROTAC assembly in a plate. This involved the o‐NBA‐NHS‐related amide coupling in the first step, followed by the addition of the POI‐L1 fragment in the second step via PANAC photoclick chemistry (Figure 2c). The reaction successfully produced the desired product with high conversions, and the conditions screening revealed that a basic PBS buffer resulted in an increasing conversion (Figure 2d), achieving up to 96% conversion as determined by UPLC–MS analysis through the seamless synthesis process in a plate (Figure 2e).

We conducted further investigation into PROTAC assembly in a plate by switching o‐NBA‐NHS‐1 to o‐NBA‐NHS‐2 and performing amide coupling with fragment E3‐L1 (Figure 1b, Method A, Figure S1A, Supporting Information). Subsequently, PROTAC conjugation involved the amide coupling product F‐2 with the POI‐L1 fragment, employing PANAC photoclick chemistry. Remarkably, the PROTAC assembly also proceeded smoothly, achieving excellent conversion in the seamless synthesis process (Figure S1B, Supporting Information). Alternatively, by initially incorporating the POI‐L1 fragment in the first step for amide coupling with o‐NBA‐NHS‐1 or o‐NBA‐NHS‐2 (Figure 1b, Method C, D), followed by PANAC conjugation of the corresponding amide coupling product with the E3‐L1 fragment in the second step, these PROTAC assemblies also exhibited superior conversions, as determined by UPLC–MS analysis. (Figures S2 and S3, Supporting Information). It is also worth mentioning that we successfully obtained the desired PROTAC products with four different linker structures through a seamless synthesis process via amide coupling followed by PANAC photoclick chemistry in a plate (Figure 1b). This was achieved by either simply switching the o‐NBA‐NHS compounds (from o‐NBA‐NHS‐1 to o‐NBA‐NHS‐2) or by altering the primary fragments (from E3‐L1 to POI‐L1) in the first step for amide couplings. Furthermore, we examined the stability of the assembled products at various buffer pH values and observed that the compounds remained relatively stable under different buffer conditions (Figure S4, Supporting Information). Altogether, these results suggest that our PROTAC assembly strategy, merging amide coupling with PANAC photoclick chemistry, could offer four different linker structures with high efficiency using one E3 ligand fragment and one POI ligand fragment. This is achieved by simply switching o‐NBA‐NHS compounds and amide coupling sequences of primary amines derived from POI and E3 ligands in a plate‐based seamless synthesis protocol.

### 2.3 High‐Throughput Synthesis of PROTAC Library with Structural Diversity via Plate‐Based Assembly

**Figure 3:**
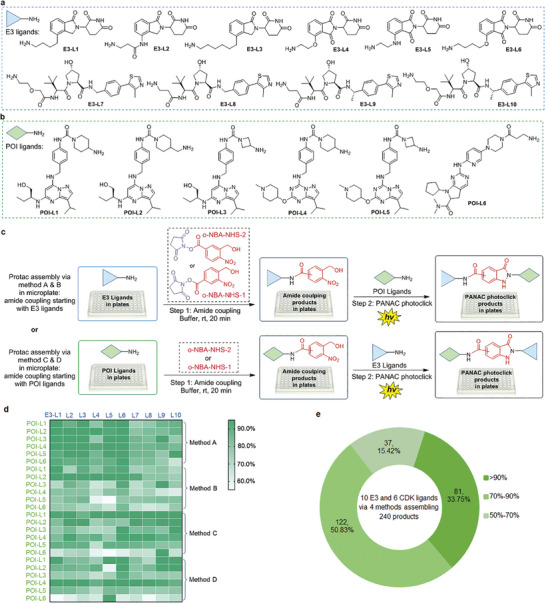
Assembled structure diversity of PROTACs from different E3 ligands and POI ligands via plate‐based synthesis. a) E3 ligands for PROTACs assembly. b) POI ligands for PROTACs assembly. c) Schematic representation of the PROTACs assembly methods and conjugation reactions in plates. d) Heatmap of the conversions of the assembled PROTACs determined by the UPLC trace and MS, heatmap colored by UPLC‐MS conversion corresponding to related E3 ligands and POI ligands. For conversion details please see Table S1 (Supporting Information). e) The pie chart showing the distribution of percentage conversion of PROTACs assembly in (d).

Our next step involved assembling PROTACs with structural diversity from different E3 ligands and POI ligands using a plate‐based synthesis protocol. We synthesized multiple E3 ligands (**Figure** **3**a, E3‐L2 – E3‐L10) targeting CRBN and VHL, the two most successful and widely used E3 ligases involved in targeted protein degradation.^[^ ^5^, ^18^, ^36^ ^]^ In addition, five different CDK9 inhibitor‐derived primary amines were also synthesized as POI ligands (Figure 3b, POI‐L1 – POI‐L5). Additionally, we synthesized a highly selective cyclin‐dependent kinase 4/6 (CDK4/6) inhibitor (Ribociclib)‐derived primary amine (POI‐L6), which does not favor the ligand binding of CDK9.^[^ ^46^ ^]^ This was done for the purpose of comparing the ligand effects and assessing the feasibility of a direct cell‐based biological evaluation of assembled PROTACs.

As illustrated in Figure 3, the amide coupling commenced with E3 ligands using either o‐NBA‐NHS‐1 or o‐NBA‐NHS‐2 in the first step, followed by the PANAC photoclick reaction with POI ligands (Figure 3c, method A, method B). Alternatively, amide coupling could also initiate from POI ligands with o‐NBA‐NHS‐1 or o‐NBA‐NHS‐2 in the first step, followed by the PANAC photoclick reaction with E3 ligands (Figure 3c, method C, method D). From the UPLC–MS analysis, we observed that the amide coupling in the first step could be completely converted to amide products in most reactions. In addition, we found that the plate‐based PROTAC assembly successfully generated 240 (e.g., 10 × 6 × 4 = 240) in the presence of a molecular plugin, either o‐NBA‐NHS‐1 or o‐NBA‐NHS‐2. This determination was based on UPLC–MS analysis of the reaction mixtures in plates (Figure 3d, heatmap). Among the 240 PROTAC assemble reactions (Figure 3e), 81 (33.7%) PROTAC molecules from 10 E3 ligands and 6 POI ligands reached greater than 90% conversion, and 203 (85%) afforded greater than 70% conversion, in the two‐step assembly process. Notably, the remaining 37 assembled PROTACs (15.4%) still demonstrated conversion rates exceeding 50% (Figure 3e). On the other hand, we noticed that during the two‐step synthesis of PROTACs, the modular assembly process only generated N‐hydroxysuccinimide (NHS) and water as by‐products. More importantly, the plate‐based PROTAC synthesis in a two‐step modular assembly protocol, without the use of toxic metal catalysts and additional chemical reagents, only generated N‐hydroxysuccinimide (NHS) and water as by‐products. Consequently, we considered the crude reaction mixtures could be directly used for cell‐based bioassays without the need for any purification.

### 2.4 Assembled PROTAC Library in Plate Directly Used for Cell‐Based Evaluation

**Figure 4:**
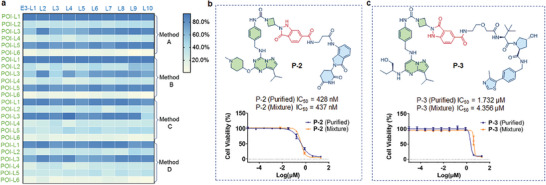
The cell growth inhibition rate of assembled PROTACs on MDA‐MB‐231 cells. a) Heatmap of cell growth inhibition rate profiles of assembled PROTACs on MDA‐MB‐231 cells, heatmap colored by cell growth inhibition rate of assembled compounds corresponding to related E3 ligands and POI ligands. Cell growth inhibition rate was measured via CCK8 analysis. For cell growth inhibition rate details, please see Table S2 (Supporting Information). b,c) PROTACs **P‐2**, **P‐3** were purified, and the cell growth inhibition data half‐maximal inhibitory concentration (IC_50_) for purified compounds showed a good correlation with data from mixture products via a direct‐to‐biology platform. *n* = three biological replicates. Error bars represent standard deviations.

With the assurance of high conversion, we conducted cellular activity evaluations with the plate‐based assembled PROTAC library. TNBC is highly heterogeneous and aggressive with very limited treatment options due to the lack of efficient targeted therapies, and thus still remains clinically challenging. Recently, targeting transcription‐associated cyclin‐dependent kinases (e.g., CDK9) to remodel transcriptional regulation has shown great promise in cancer therapy.^[^ ^46^, ^47^ ^]^ Increasing evidence supports that depletion of CDK9 activity led to effective antiproliferation effect in both in vitro and in vivo TNBC models, indicating that targeting CDK9 is a promising avenue for developing new TNBC therapeutics; however, this endeavor is still at an early stage and there remains a paucity of CDK9 degraders specifically designed for TNBC treatment.^[^ ^48^ ^]^ Thus, we chose the TNBC cell line MDA‐MB‐231 for direct cell‐based evaluation. We initially investigated the effect of the by‐product N‐hydroxysuccinimide (NHS), generated from the amide coupling step in PROTAC assembly, on cell growth inhibition. The NHS compound demonstrated no activity even as the concentration increased up to 20 µm in the TNBC cell line, and also there was no effect of the PBS buffer used for PROTAC assembly in plates (Figure S5, Supporting Information). These observations indicate that both NHS and PBS buffer almost have no effect on cell growth inhibition, supporting the notion that the crude mixtures of reactions were suitable for direct cell‐based bioassays. We also tested the antiproliferation activity for a selection of structurally diversified E3‐L and all POI‐L (POI‐L1∼L6) on MDA‐MB‐231 and MDA‐MB‐468 cells. All tested POI‐L and E3‐L fragments did not show obvious cell growth inhibition (Figure S10, Supporting Information). We diluted each of the product mixtures to concentrations of 20 and 2 µm in the medium for the purpose of cell growth inhibition evaluations (**Figure** **4**A; Figure S6, Supporting Information). Based on the analysis of the cell growth inhibition rate profiles, we observed that a diverse array of anticipated assembled product mixtures exhibited high activities for cell growth inhibition (Figure 4A, e.g, at a concentration of 2 µm). This suggests that assembled PROTACs via plate‐based synthesis are applicable for direct cell‐based bioassay, enabling accelerating the discovery of new structures and potential degraders.

Upon further comparison of the assembled product mixtures at different concentrations (20 vs 2 µm), we observed that numerous product mixtures exhibited superior cell growth inhibitory activities both at high and low concentrations (Figure S6, Supporting Information). Notably, one PROTAC (Figure 4b and **P‐2**) assembled from CRBN ligands E3‐L2 and POI‐L3 exhibited an IC_50_ value ≈400 nm in MDA‐MB‐231 cells. On the other hand, among the VHL ligand‐derived PROTACs, we also identified PROTAC, which exhibited good cell growth inhibitory activity (Figure 4c and **P‐3**). Of note, a CDK9 PROTAC derived from a VHL ligand has not been reported to date. We subsequently purified these two PROTACs (**P‐2** and **P‐3**), and observed an excellent correlation between crude mixture values and purified product values (Figure 4b,c). This indicates that the direct cell‐based bioassay with assembled product mixtures in plates is feasible, and our strategy streamlines the PROTAC synthesis and biological evaluation process. Interestingly, the majority of PROTACs derived from the CDK4/6 inhibitor showed low cell growth inhibitory activities (Figure 4a, POI‐L6 lane, and Figure S6, Supporting Information, POI‐L6 lane). This observation is consistent with a previous report indicating that PROTACs based on CDK4/6 inhibitor and CRBN ligands are not effective on MDA‐MB‐231 cells in protein degradation and cytotoxicity assays.^[^ ^47^ ^]^ Taken together, these results demonstrate that the direct cell‐based bioassay using the assembled PROTAC library in plates is a practical approach for discovering effective PROTACs for CDK9 protein. In comparison, the prevalent synthetic process of PROTACs demands multiple synthesis‐purification steps, resulting in a time‐consuming and labor‐intensive process, primarily attributed to their structural complexity. As a result, the purification process presents a challenge, mainly due to the presence of multiple functional groups in their structures and the likelihood of undesired by‐products. By integrating amide coupling and PANAC photoclick chemistry into a plate‐based synthetic process, our established direct‐to‐biology strategy enables the seamless transfer of crude reaction mixtures to cell‐based biological evaluation, obviating the requirement for resynthesizing analogs with more stable linkages in the next stage and for the purification of reaction mixtures.

### 2.5 Application of the Direct‐to‐Biology Platform for Screening Potential CRBN Ligands and Cell‐Based Bioassay for Discovery of PROTACs

**Figure 5:**
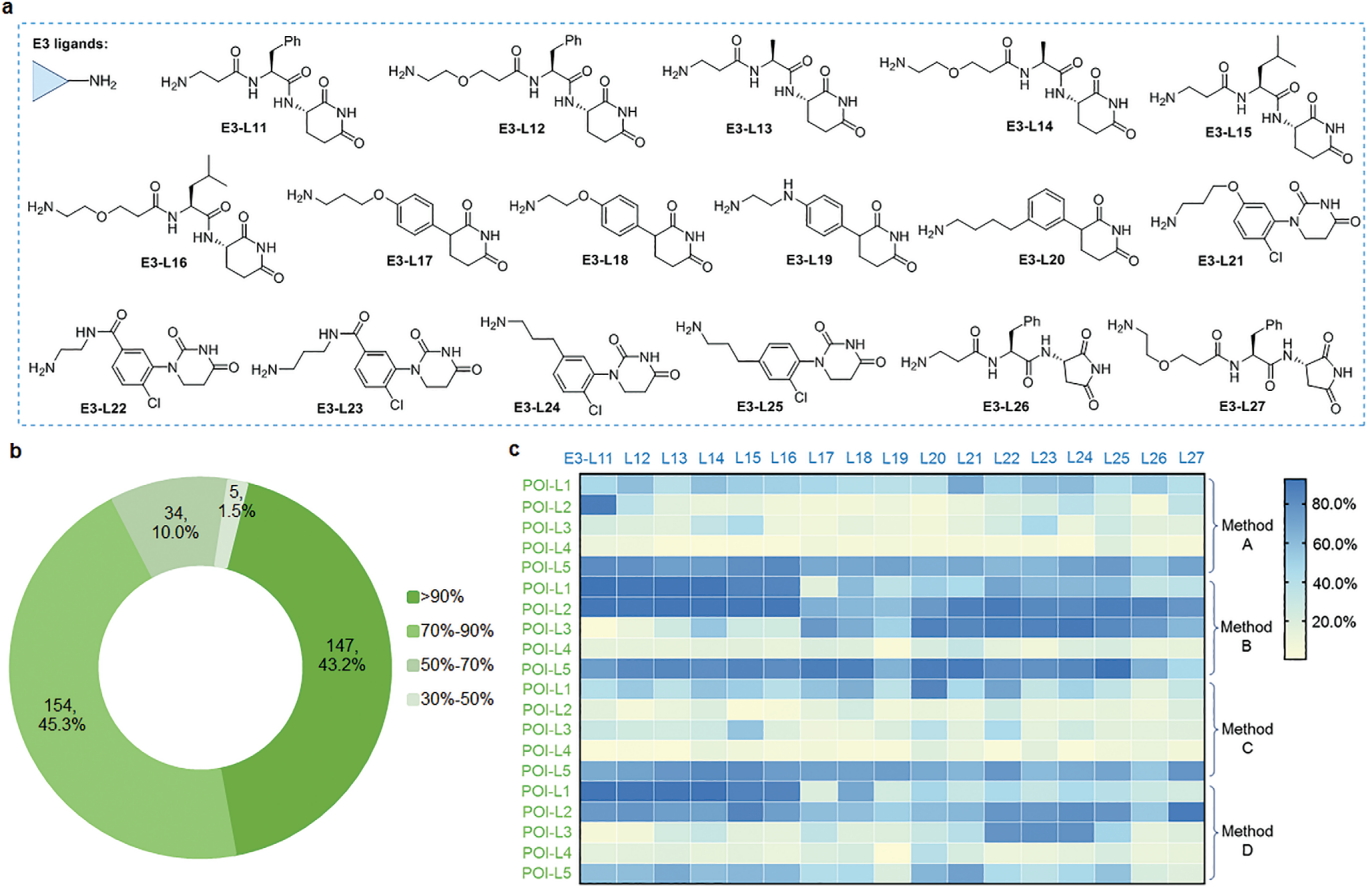
Application of direct‐to‐biology platform to screen different kinds of E3 ligands for PROTACs discovery. a) Different kinds of E3 ligands for assembling structure diversity PROTACs. b) The pie chart showing the distribution of percentage conversion of PROTACs assembly with E3 ligands (E3‐L11‐27) and POI ligands (POI‐L1‐5), see Supporting Information for the heat map of the conversions, determined by the UPLC trace and MS analysis. c) Heatmap of cell growth inhibition rate profiles of assembled PROTACs from (b) on MDA‐MB‐231 cells, heatmap colored by cell growth inhibition rate of assembled compounds corresponding to related E3 ligands and POI ligands. For cell growth inhibition rate details please see Table S4 (Supporting Information).

To explore the chemical space of PROTACs and discover more effective PROTACs for targeted protein degradation, we further applied the direct‐to‐biology platform to cell‐based bioassay and PROTAC discovery. We synthesized the recently reported CRBN binders (**Figure** **5**, E3‐L11 – E3‐L27) for our research.^[^ ^36^, ^38^, ^49^ ^]^ Again, the tested E3‐L fragments did not show obvious cell growth inhibition on MDA‐MB‐231 and MDA‐MB‐468 cells (Figure S10, Supporting Information). Similarly, we performed plate‐based PROTAC assembly from these CRBN binders (Figure 5a, E3‐L11 – E3‐L27) and POI ligands (Figure 3b, POI‐L1 – POI‐L5). Following UPLC–MS analysis, the plate‐based PROTAC assembly successfully yielded 340 anticipated assembled molecules (e.g., 17 × 5 × 4 = 340) using 17 E3 ligands and 5 POI ligands in the presence of molecular plugin, either o‐NBA‐NHS‐1 or o‐NBA‐NHS‐2. Out of the 340 PROTAC assembly reactions, 147 (43%) achieved greater than 90% conversion, and 301 (88%) resulted in greater than 70% conversions (Figure 5b; Figure S7 and Table S3, Supporting Information). Taken together, these results demonstrate that our modular assembly strategy in the plate is applicable and efficient for high‐throughput synthesis of a diverse PROTAC library from various CRBN binders, thereby providing structural diversity. Moreover, utilizing operational simplicity and mild conditions, in addition to readily accessible fragments of POI and E3 ligand‐derived primary amines, and demonstrating high efficiency in PROTAC construction, we anticipate that our modular assembly strategy holds significant promise for the automated, miniaturized, and high‐throughput chemical synthesis of structurally diverse bioactive compound libraries.^[^ ^24^, ^28^ ^]^

**Figure 6:**
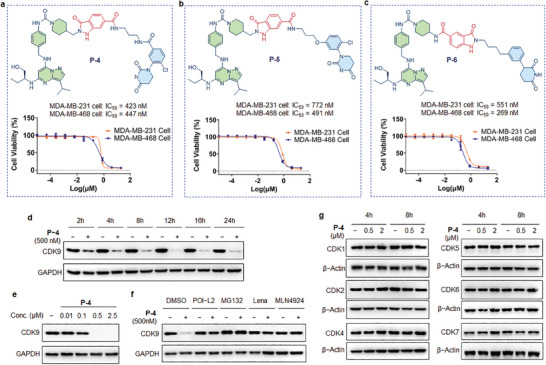
The cell growth inhibitory activities of the purified PROTACs in MDA‐MB‐231 and MDA‐MB‐468 TNBC cells and validation of the compound **P‐4** for protein degradation. a) The growth inhibitory activities of PROTAC **P‐4** in two kinds of TNBC cells. b) The growth inhibitory activities of PROTAC **P‐5** in two kinds of TNBC cells. c) The growth inhibitory activities of PROTAC **P‐6** in two kinds of TNBC cells. For a–c, *n* = three biological replicates. Error bars represent standard deviations. d) Results of time‐dependent degradation test of compound **P‐4** on CDK9 with indicated concentrations in MDA‐MB‐231 cells. e) Immunoblot of CDK9 and glyceraldehyde‐3‐phosphate dehydrogenase (GAPDH) following 8 h treatment with DMSO or the indicated concentrations of compound **P‐4** in MDA‐MB‐231 cells. f) Western blotting for CDK9 and GAPDH after 4 h pretreatment with DMSO, POI‐L2 (10 µm), MG132 (5 µm), Lena (10 µm), MLN4924 (1 µm), followed by a 4 h treatment with compound **P‐4** at 500 nm in MDA‐MB‐231 cells. g) Immunoblot of CDK proteins and β‐actin following 4 or 8 h of incubation with DMSO or the indicated concentrations of compound **P‐4** in MDA‐MB‐231 cells.

Next, we conducted screening for growth inhibitory activity on TNBC MDA‐MB‐231 cells using the assembled PROTAC library. Through an examination of cell growth inhibition rate profiles, many products displayed significant cell inhibitory activities (Figure 5c; Figure S8, Supporting Information). Following the synthesis and purification of three selected PROTACs (**Figure** **6**a–c and **P‐4** – **P‐6**), the purified PROTAC **P4** demonstrated significant cell inhibitory activity against MDA‐MB‐231 cells, achieving an IC_50_ value of 423 nm (Figure 6a). In addition, PROTAC **P‐6** exhibited superior cell inhibitory activity compared to PROTAC **P‐4** on other TNBC MDA‐MB‐468 cells, with an IC_50_ value of 269 nm (Figure 6c). These selected PROTACs also showed effective anti‐proliferation activities against other CDK9‐dependent cell lines (Table S5, Supporting Information). Altogether, our results illustrate the potential of the direct‐to‐biology platform for screening a range of CRBN binders, and this approach offers a diverse array of PROTAC structures, contributing to enhanced activities against cancer cells.

In light of recent research highlighting the critical impact of linkers on PROTAC activity,^[^ ^14^, ^15^, ^16^ ^]^ we wondered whether the rigidity of the indazolone linker motifs generated via the PANAC photoclick reaction has an effect on the activities of PROTACs. Therefore, we chose to substitute the indazolone motif in PROTAC **P‐4** and **P‐5** with a carbon chain to derive corresponding compounds (Figure S9, Supporting Information). Cell growth inhibition assays indicated a noticeable decline in activities on MDA‐MB‐231 cells associated with the flexible carbon chain linker in the heterobifunctional compounds (Figure S9, Supporting Information). This observation indicates that the rigidity of indazolone motifs positively influences cellular activities, particularly in terms of linker effects. In principle, our strategy would be practical as long as both NHS‐ester and o‐NBA functionalities are retained in the linker for PROTAC assembly, regardless of whether they are in the compacted forms (e.g., o‐NBA‐NHS‐1 and o‐NBA‐NHS‐2 in Figure 1,) or separated by a diverse structural spacer. Thus, we envisage that diverse linkers, in which NHS‐ester and o‐NBA moieties are connected by different spacers (in length, rigidity, hydrophobicity, etc.) would make our methodology much tunable for PROTACs activity optimization. Altogether, the photoclick strategy for constructing a PROTAC library, also suggests the potential for optimizing the intended PROTACs with indazolone linkers in PROTAC development to enhance their activity.

### 2.6 Validation of the Assembled PROTAC for Targeted Protein Degradation

We selected the screened PROTAC **P‐4** (Figure 6a) for the CDK9 protein degradation experiments. We examined the degradation efficiency of CDK9 protein induced by **P‐4** at various time points (Figure 6d). We found that the target protein CDK9 in MDA‐MB‐231 cells almost could be degraded after the treatment with **P‐4** at 500 nm for 8 h. Subsequently, based on immunoblot results, **P‐4** is a potent degrader targeting CDK9 in a dose‐dependent manner (Figure 6e). The degradation profiles indicate that **P‐4** could induce the degradation of CDK9 protein at concentrations as low as 100 nm in TNBC cells after an 8‐h treatment (Figure 6e).

Next, the mechanism of protein CDK9 degradation induced by **P‐4** was further investigated (Figure 6f). We chose compound POI‐L2 (Figure 3b), a ligand for CDK9 protein but lacking the linker and E3 ligand fragment, as a competitive CDK9 binder in comparison to compound **P‐4**. We pre‐treated MDA‐MB‐231 cells with compound POI‐L2 at a concentration of 10 µm for 4 h. Subsequently, 500 nm of **P‐4** was added, and the cells were treated for an additional 4 h. Western blotting analysis revealed that the addition of compound POI‐L2 effectively blocked the degradation of CDK9 induced by **P‐4** (Figure 6f), thus, these results clearly confirmed the degradation of CDK9 protein by compound **P‐4** depending on the binding to the CDK9 protein. Moreover, in the presence of the CRBN ligand lenalidomide (lena), the degradation of CDK9 protein was also effectively blocked, which also obviously indicated that the degradation of CDK9 protein induced by **P‐4** was CRBN dependent (Figure 6f). Furthermore, the proteasome inhibitor MG132 and the NEDD8‐activating enzyme (NAE) inhibitor MLN4924 also blocked CDK9 protein degradation, revealing that degradation of CDK9 was dependent on the functions of proteasome and NAE. Altogether, these results demonstrate that in order to achieve CDK9 degradation, compound **P‐4** must bind to both target protein CDK9 and E3 ligase CRBN, and the subsequent protein degradation requires the UPS, which is consistent with the mechanism of action of PROTAC molecules. In addition, as expected, MDA‐MB‐231 treated with **P‐3** and **P‐6** at 2.5 µm for 8 h, demonstrated effective degradation of CDK9 protein (Figure S11, Supporting Information).

To assess the selectivity of PROTAC **P‐4** in degrading CDK9 protein, we analyzed degradation profiles at different time points (Figure 6g and 4 and 8 h). Western blotting assays revealed that, at concentrations ranging from 0.5 to 2.0 µm, PROTAC **P‐4** did not influence the protein levels of other members of the CDK family, including CDK1, 2, 4, 5, 6, and 7 (Figure 6g). These findings collectively demonstrate that the PROTAC **P‐4** is a potent and selective CDK9 degrader for MDA‐MB‐231 cells. Collectively, by employing this direct‐to‐biology platform for PROTACs synthesis and screening, we smoothly found potent CDK9 degraders that efficiently inhibit TNBC cell growth and induce rapid targeted degradation of CDK9, presenting a highly promising degrader for targeted therapy of TNBC.

## 3 Conclusion

In summary, we have successfully developed a direct‐to‐biology strategy to streamline PROTAC library synthesis on a plate and subsequently direct biological evaluation. By integrating amide coupling and PANAC photoclick chemistry in a plate‐based synthetic process, this approach enables the seamless transfer of crude reaction mixtures to cell‐based bioassay without the necessity for purification. Harnessing the flexibility in amide coupling sequences of primary amine fragments through the use of molecular plugin o‐NBA‐NHSs, followed by the PANAC photoclick chemistry, our plate‐based synthetic protocol successfully yields PROTACs library with high efficiency and structural diversity, which significantly expands the chemical space available for degrader screening. Moreover, employing this direct‐to‐biology platform for PROTACs evaluation and discovery, coupled with the assessment of a few isolated heterobifunctional PROTACs, we smoothly identified potent CDK9 degraders that effectively inhibit TNBC cell growth and induce rapid targeted degradation of CDK9, offering promising degrader for targeted therapy of TNBC.

In contrast to the prevailing PROTACs discovery approach, which relies on an empirical design−synthesis−evaluation process driven by many cycles of time‐consuming multi‐step synthesis and bioassay data collection, our developed direct‐to‐biology platform provides hundreds of heterobifunctional molecules assembled from different POI and E3 ligands derived primary amines in a short time, thus, enables us to accelerate the optimization and discovery of degraders. In addition, in comparison to previous click reactions used for constructing single heterobifunctional structures, the flexibility of o‐NBA‐NHSs linkerology and amide coupling sequences of ligand‐derived primary amines, significantly expand the amounts of the available products for potential degrader screening. Furthermore, unlike other direct‐to‐biology strategies for PROTACs synthesis and evaluation, our strategy provides the heterobifunctional molecules with structure stability, realizing high‐throughput and seamless synthesis of PROTAC library in plate, without the need for additional coupling chemicals and further purification process, followed by direct transfer of the crude reaction mixtures to cell‐based evaluations. Collectively, our approach substantially enhances the efficiency of exploring chemical space, accelerating the discovery process of PROTACs. As primary amines represent one of the most abundant functional groups in synthetic chemistry, by extending this method to encompass a broader range of POI and E3 ligand‐derived primary amines, we anticipate that our direct‐to‐biology strategy will provide a comprehensive opportunity for high‐throughput synthesis of PROTAC libraries, thereby expediting the optimization and discovery of degraders.

## 4 Experimental Section

### Methods of Light‐Induced Reactions in Plates and LC‐MS Analysis—Method A

Reaction buffer (20 mm PBS)/DMSO = 1:1, pH 10.5. 2 µL stock of E3 ligand (100 mm) was mixed with equal volume of o‐NBA‐NHS‐2 stock (100 mm) in 46 µL buffer followed by shaking for 20 min. Next, to the reaction mixture, 2 µL stock of POI ligand (100 mm) and 48 µL buffer were added (final concentration of all reactants, 2 mm). The mixture was then exposed to 365 nm UV for 10 min and further incubated for 5 min. Repeat the UV irradiation and incubation once. The reaction crude was directly used in the inhibition rate test or diluted with ACN/H2O = 1:1 solution for LC‐MS analysis. Method B: the protocol is similar with Method A, only with the difference in switching the o‐NBA‐NHS‐2 to o‐NBA‐NHS‐1. Method C: Reaction buffer (20 mm PBS)/DMSO = 1:1, pH 10.5. 2 µL stock of POI ligand (100 mm) was mixed with an equal volume of o‐NBA‐NHS‐2 stock (100 mm) in 46 µL buffer followed by shaking for 20 min. Next, to the reaction mixture, 2 µL stock of E3 ligand (100 mm) and 48 µL buffer were added (final concentration of all reactants, 2 mm). The mixture was then exposed to 365 nm UV for 10 min and further incubated for 5 min. Repeat the UV irradiation and incubation once. The reaction crude was directly used in the inhibition rate test or diluted with ACN/H2O = 1:1 solution for LC‐MS analysis. Method D: the protocol is similar to Method C, only with the difference in switching the o‐NBA‐NHS‐2 to o‐NBA‐NHS‐1.

### Cellular Inhibition Rate Assay

The human TNBC cells MDA‐MB‐231 and MDA‐MB‐468 used in this work were all purchased from the Cell Bank of Type Culture Collection, Chinese Academy of Sciences (Shanghai, China). MDA‐MB‐231 and MDA‐MB‐468 cells were cultured in Leibovitz's (L‐15) media supplemented with 10% FBS and 1% penicillin−streptomycin antibiotics in an incubator without CO_2_ at 37 °C. For the cell viability assay, 3 × 10^4^ MDA‐MB‐231 cells and 3 × 10^4^ MDA‐MB‐468 cells of 80 µL cell suspension were plated in 96‐well cell culture plates. The tested compounds were gradient‐diluted with the corresponding media, 20 µL of the diluted compound was added to each well, and then cells were cultured at 37 °C for another 4 days. The cell viability was measured by the Cell Counting Kit‐8 reagent (DOJINDO, Cat. CK04) according to the manufacturer's instructions.

### Cellular Viability Assay

The 3 × 10^4^ MDA‐MB‐231 cells and 3 × 10^4^ MDA‐MB‐468 cells of 90 µL cell suspension were plated in 96‐well cell culture plates. The tested compounds were gradient‐diluted with the corresponding media, 10 µL of the diluted compound was added to each well, and then cells were cultured at 37 °C for another 4 days. The cell viability was measured by the Cell Counting Kit‐8 reagent (DOJINDO, Cat. CK04) according to the manufacturer's instructions.

### Western Blotting Assay

Cells were lysed with cold RIPA lysis buffer (Beyotime, P0013B) containing the complete protease inhibitor cocktail (ROCHE) and 1 mm PMSF for 30 min on ice before insoluble debris was pelleted by centrifugation at 4 °C at 12 000 g for 10 min. The protein concentration was determined by a BCA Protein Assay Kit (Thermo Fisher Scientific, 23225), and the absorbance at 562 nm was measured by spectrophotometry (Tecan Spark 10 m). The samples were separated by 10% sodium dodecyl sulfate‐PAGE using 10−20 µg of protein per well and transferred to 0.2 µm PVDF membrane (Bio‐Rad). After blocking with TBS containing 0.1% Tween‐20 (TBS‐T) with 5% skimmed milk, the membrane was incubated with primary antibodies. The membranes were washed three times for 5 min with TBS‐T before incubating with HRP‐conjugated secondary antibodies in TBS‐T with 5% milk for 1 h at room temperature. Then, the membranes were washed three times for 5 min with TBS‐T. Immunodetection was performed using the SuperSignal West Immunodetection was performed using the SuperSignal West Pico PLUS Chemiluminescent Substrate (Thermo Fisher Scientific, 34577).

## Conflict of Interest

The authors declare no conflict of interest.

## Supporting information

Supporting Information

## Data Availability

The data that support the findings of this study are available in the supplementary material of this article.
