# Supplementary material for: Accelerating PROTACs Discovery Through a Direct‐to‐Biology Platform Enabled by Modular Photoclick Chemistry
Source: Adv Sci (Weinh). 2024 Apr 30;11(26):2400594. doi: 10.1002/advs.202400594 (PMC11234393; doi:10.1002/advs.202400594)
Supplement: Supplementary file 1 — Supporting Information [file ADVS-11-2400594-s001.pdf]

ADVANCED SCIENCE
Open Access

Supporting Information

for *Adv. Sci.*, DOI 10.1002/advs.202400594

Accelerating PROTACs Discovery Through a Direct-to-Biology Platform Enabled by Modular Photoclick Chemistry

Ke-Nian Yan, Yong-Qiang Nie, Jia-Yu Wang, Guang-Liang Yin, Qia Liu, Hao Hu, Xiaoxia Sun and Xiao-Hua Chen**

Supporting Information

Accelerating PROTACs Discovery through a Direct-to-Biology Platform Enabled by Modular Photoclick Chemistry

Ke-Nian Yan,[1, 5] † Yong-Qiang Nie,[2,] † Jia-Yu Wang,[3,] † Guang-Liang Yin,[1, 5] Qia Liu,[4, 5] Hao Hu,[1] Xiaoxia Sun[2] * and Xiao-Hua Chen[1, 3, 4, 5] *

[1] State Key Laboratory of Drug Research, Shanghai Institute of Materia Medica, Chinese Academy of Sciences, Shanghai, 201203, China.

[2] Jiangxi Key Laboratory of Organic Chemistry, Jiangxi Science and Technology Normal University, Nanchang, 330013, China.

[3] School of Chinese Materia Medica, Nanjing University of Chinese Medicine, Nanjing, 210023, China.

[4] School of Pharmaceutical Science and Technology, Hangzhou Institute for Advanced Study, University of Chinese Academy of Sciences, Hangzhou, China.

[5] University of Chinese Academy of Sciences, Beijing 100049, China.

† These authors contributed equally to this work.

*Corresponding Author(s): Xiao-Hua Chen: xhchen@simm.ac.cn; Xiaoxia Sun: xxsun@jxstnu.edu.cn

Table of contents

Supplementary Figures and Table S3

Methods S29

1.1 General information S29

1.2 Methods of light-induced reactions in plates and LC-MS analysis. S30

1.3 Methods of cellular experiment S32

1.4 Synthesis methods and NMR data S33

^{1}H NMR and ^{13}C NMR Spectra S55

Reference S69

Supplementary Figures and Table

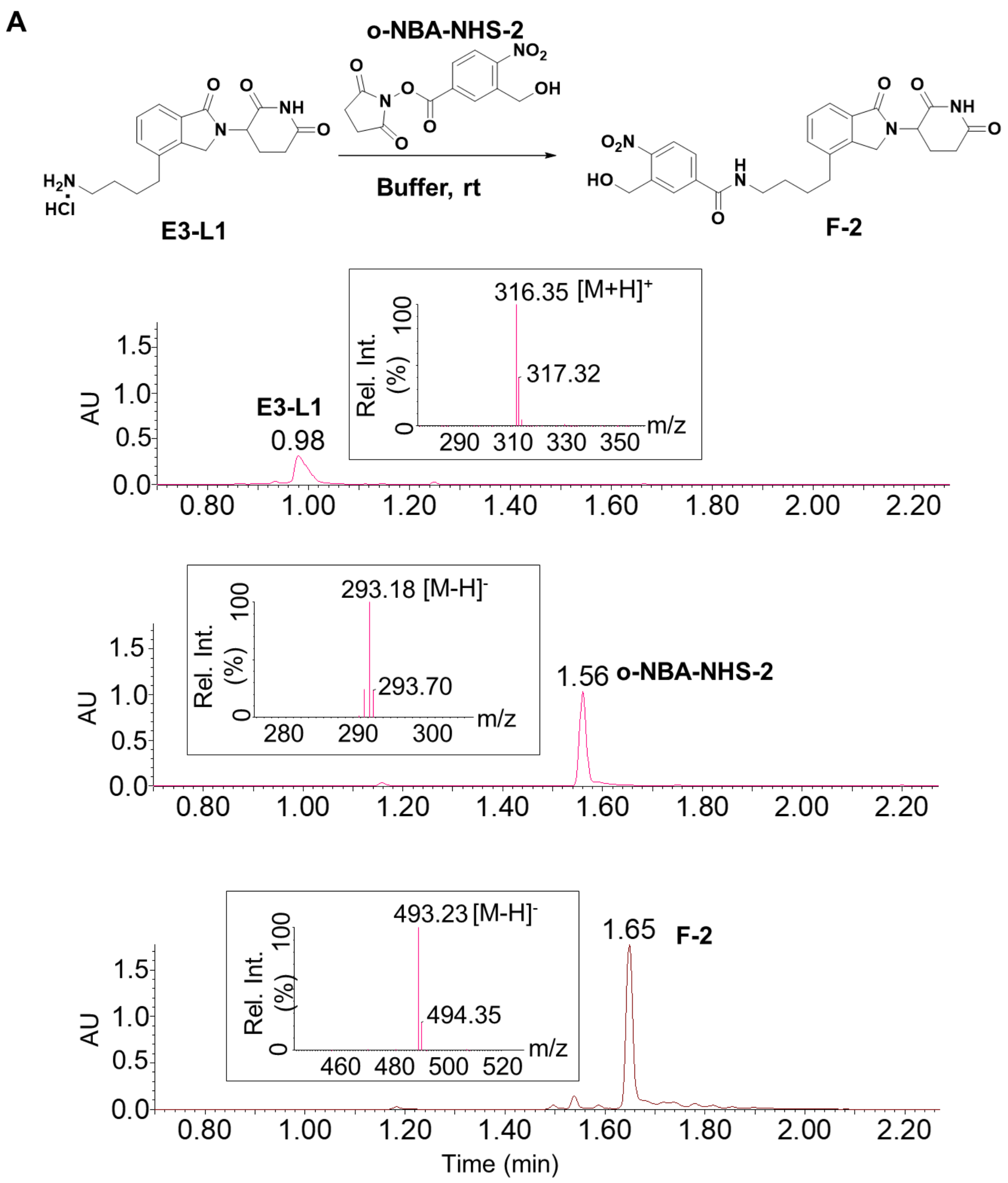

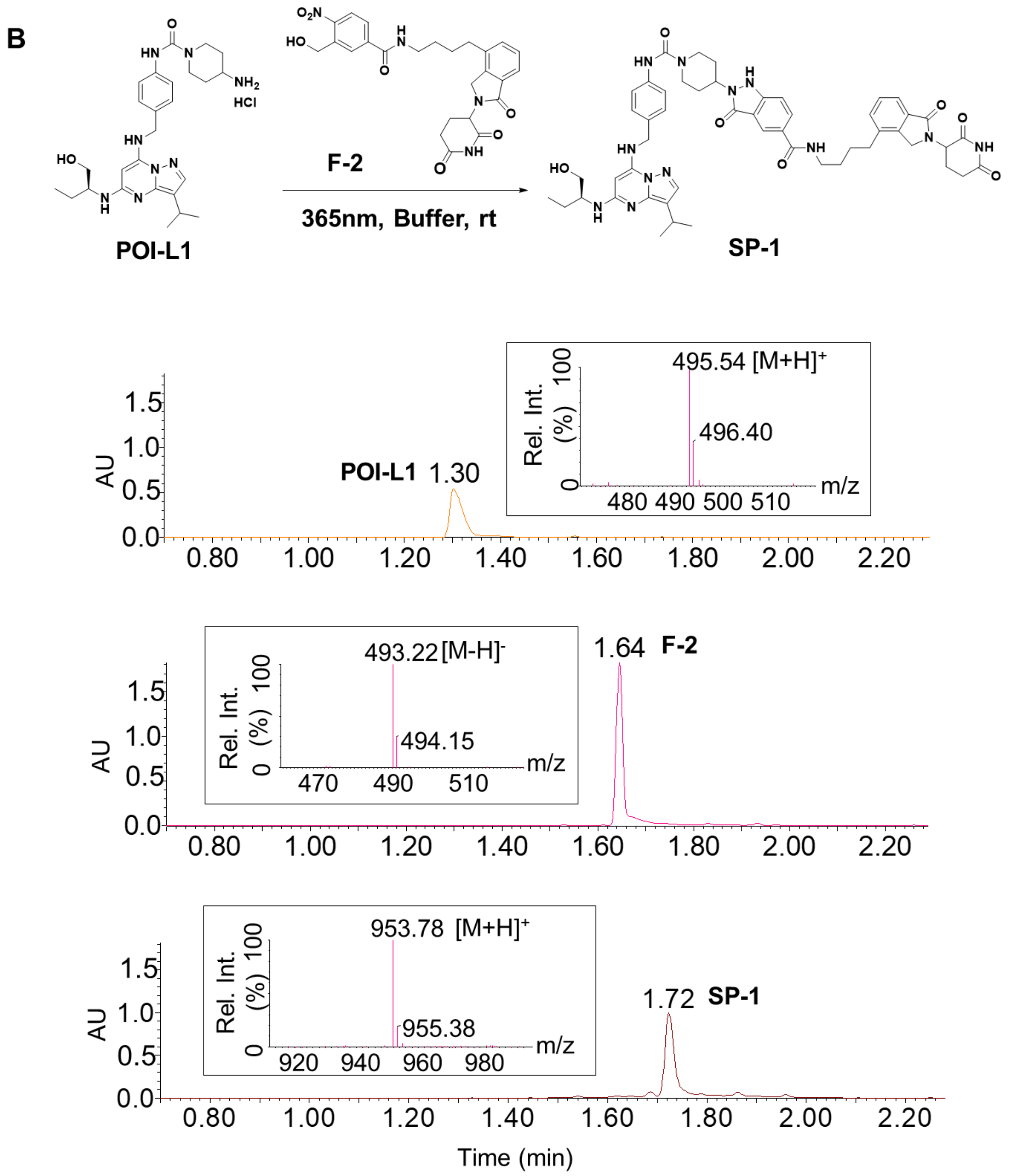

Figure S1. Wellplate-based PROTAC assembly for **SP-1**. (A) Conjugation of E3 ligand (E3-L1) with o-NBA-NHS-2 in wellplate, and the UPLC trace and Mass spectrum validation of the amide coupling efficiency. (B) PANAC photoclick conjugation of POI ligand (POI-L1) with amide coupling product (**F-2**) in wellplate, and the UPLC trace and Mass spectrum validation of the PANAC photoclick efficiency. The reaction conditions of the two steps are the same as the corresponding steps in Figure 2 in the main text.

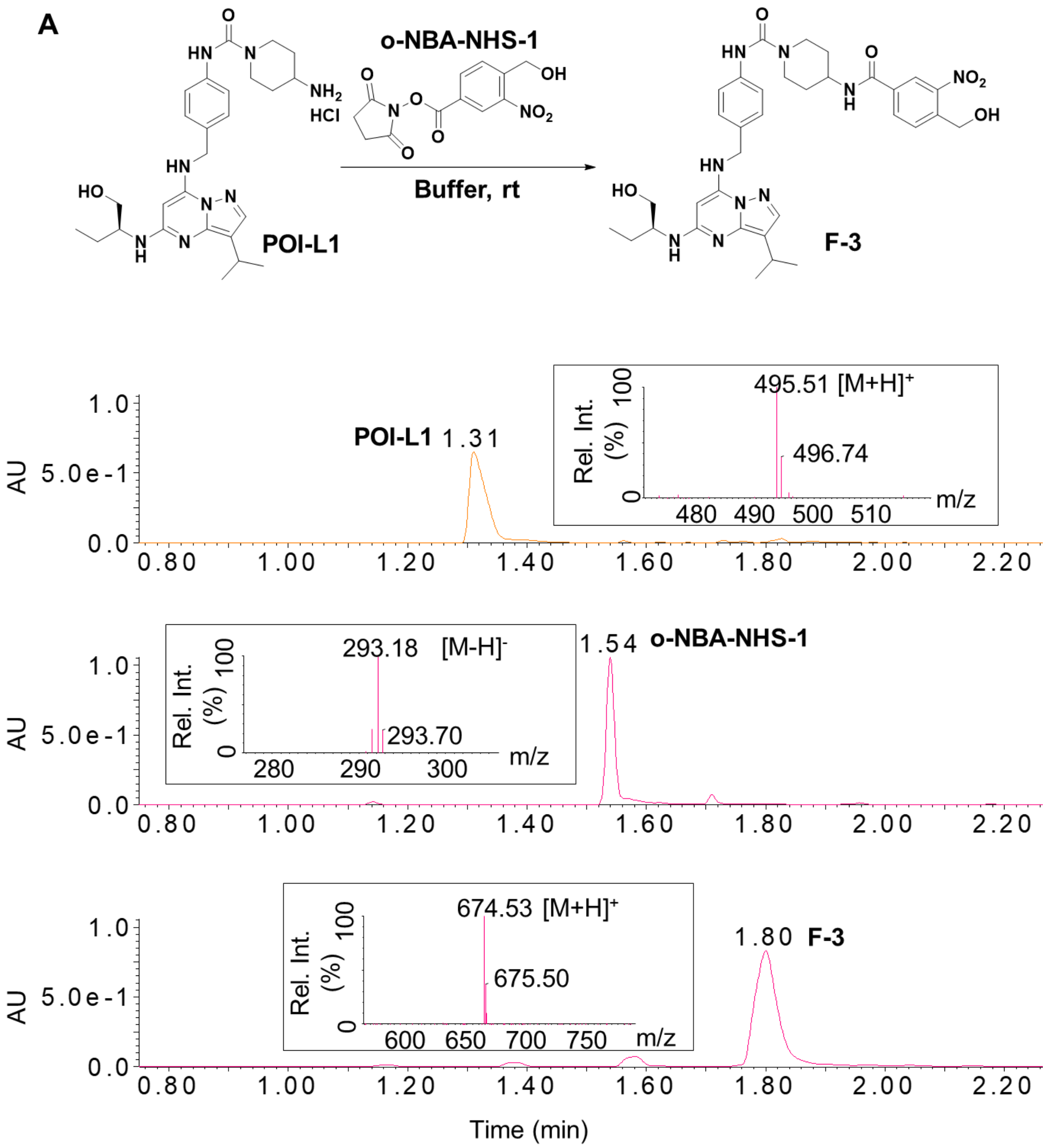
A
o-NBA-NHS-1
Buffer, rt
POI-L1
F-3
AU
1.0
5.0e-1
0.0
POI-L1 1.31
495.51 [M+H]+
496.74
Rel. Int. (%)
m/z
480 490 500 510
1.54 o-NBA-NHS-1
293.18 [M-H]-
293.70
280 290 300
1.80 F-3
674.53 [M+H]+
675.50
600 650 700 750
0.80 1.00 1.20 1.40 1.60 1.80 2.00 2.20
Time (min)

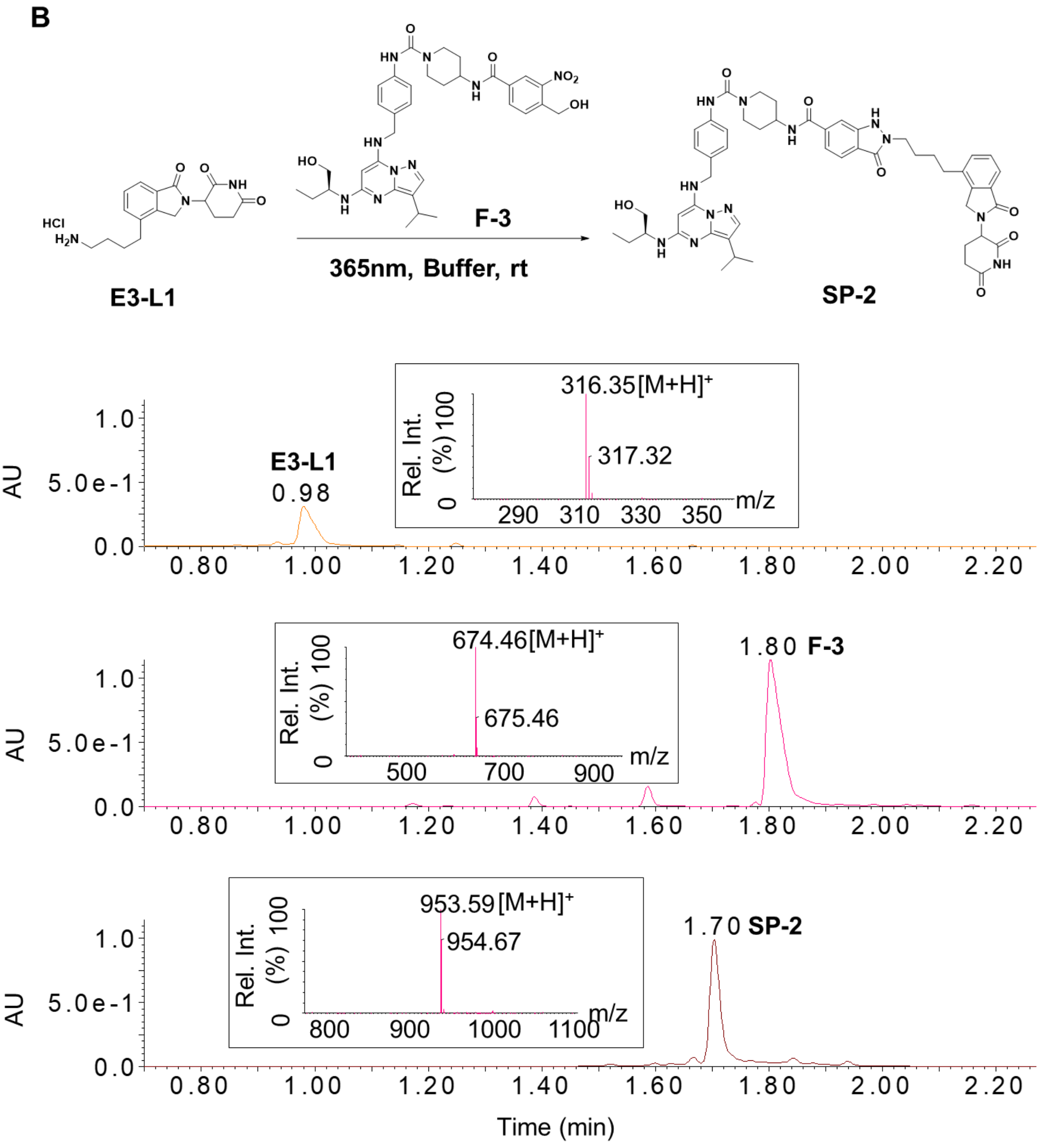

Figure S2. Wellplate-based PROTAC assembly for **SP-2**. (A) Conjugation of POI ligand (POI-L1) with o-NBA-NHS-1 in wellplate, and the UPLC trace and Mass spectrum validation of the amide coupling efficiency. (B) PANAC photoclick conjugation of E3 ligand (E3-L1) with amide coupling product (**F-3**) in wellplate, and the UPLC trace and Mass spectrum validation of the PANAC photoclick efficiency. The reaction conditions of the two steps are the same as the corresponding steps in Figure 2 in the main text.

A

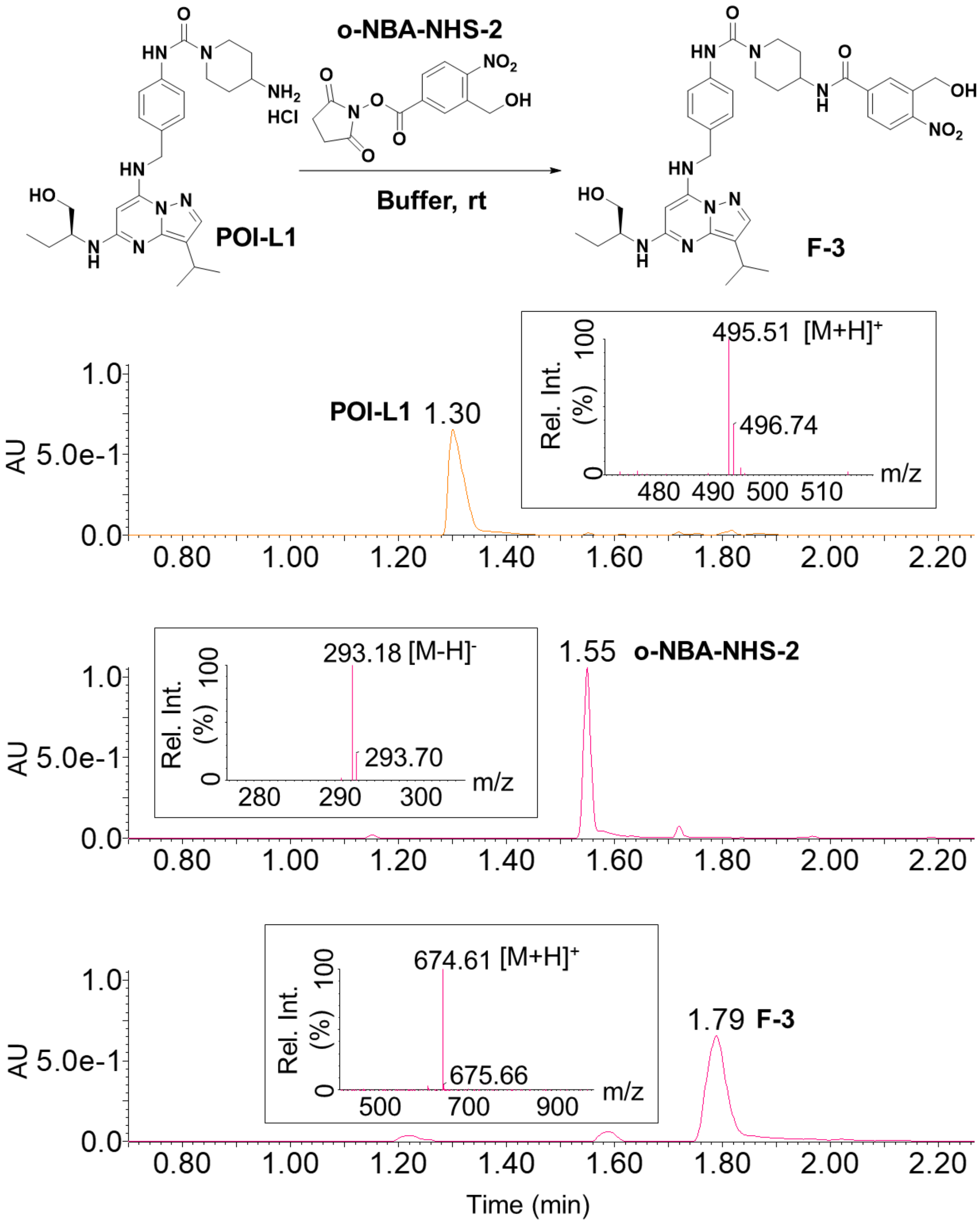
o-NBA-NHS-2
Buffer, rt
POI-L1
F-3
POI-L1 1.30
495.51 [M+H]+
496.74
1.55 o-NBA-NHS-2
293.18 [M-H]-
293.70
1.79 F-3
674.61 [M+H]+
675.66
Rel. Int. (%)
m/z
AU
Time (min)

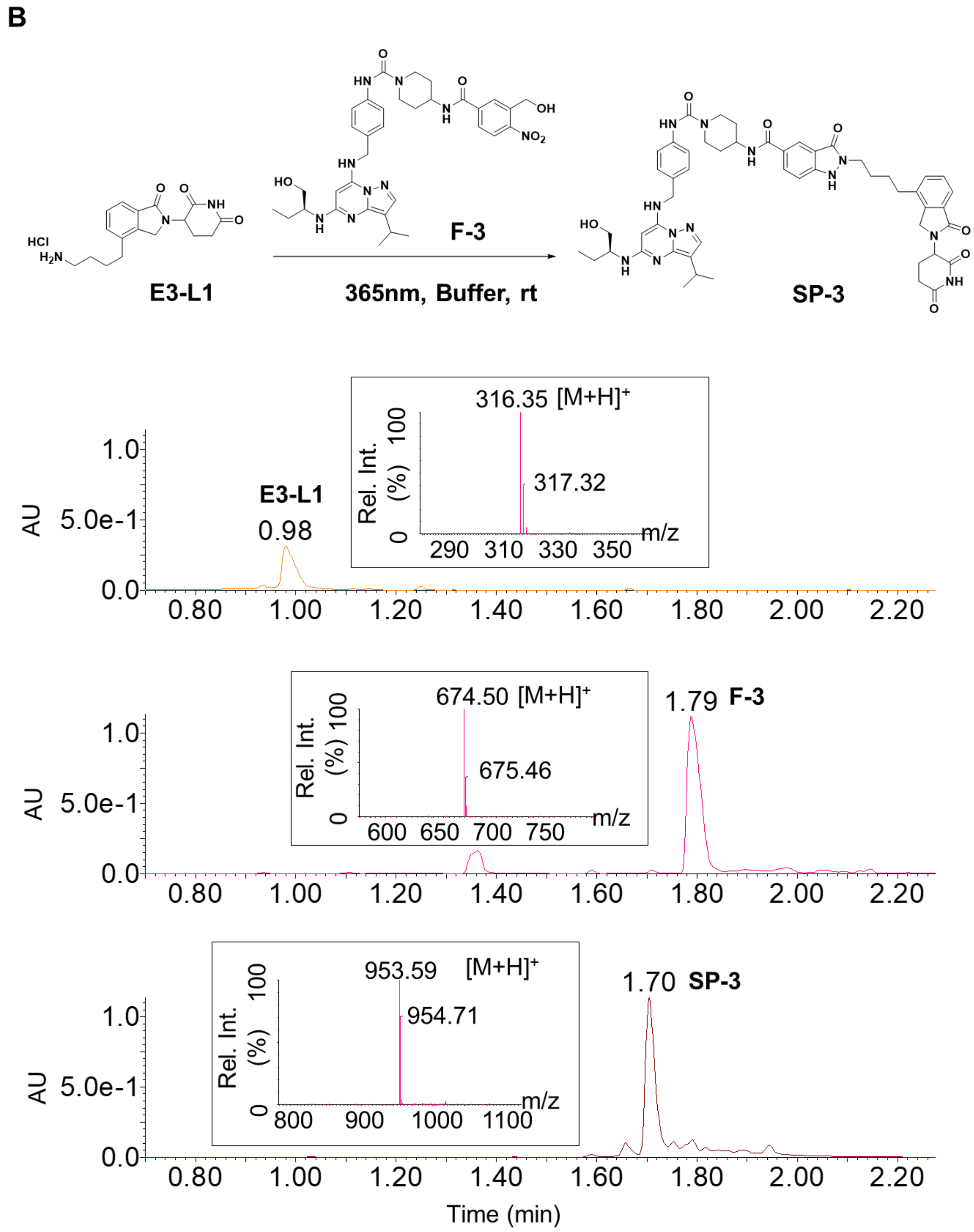

Figure S3. Wellplate-based PROTAC assembly for **SP-3**. (A) Conjugation of POI ligand (POI-L1) with o-NBA-NHS-2 in wellplate, and the UPLC trace and Mass spectrum validation of the amide coupling efficiency. (B) PANAC photoclick conjugation of E3 ligand (E3-L1) with amide coupling product (**F-4**) in wellplate, and the UPLC trace and Mass spectrum validation of the PANAC photoclick efficiency. The reaction conditions of the two steps are the same as the corresponding steps in Figure 2 in the main text.

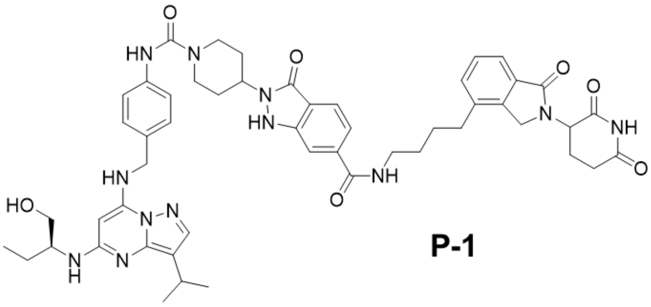

P-1

(50mM PBS)/MeOH = 1:1, pH = 4.0

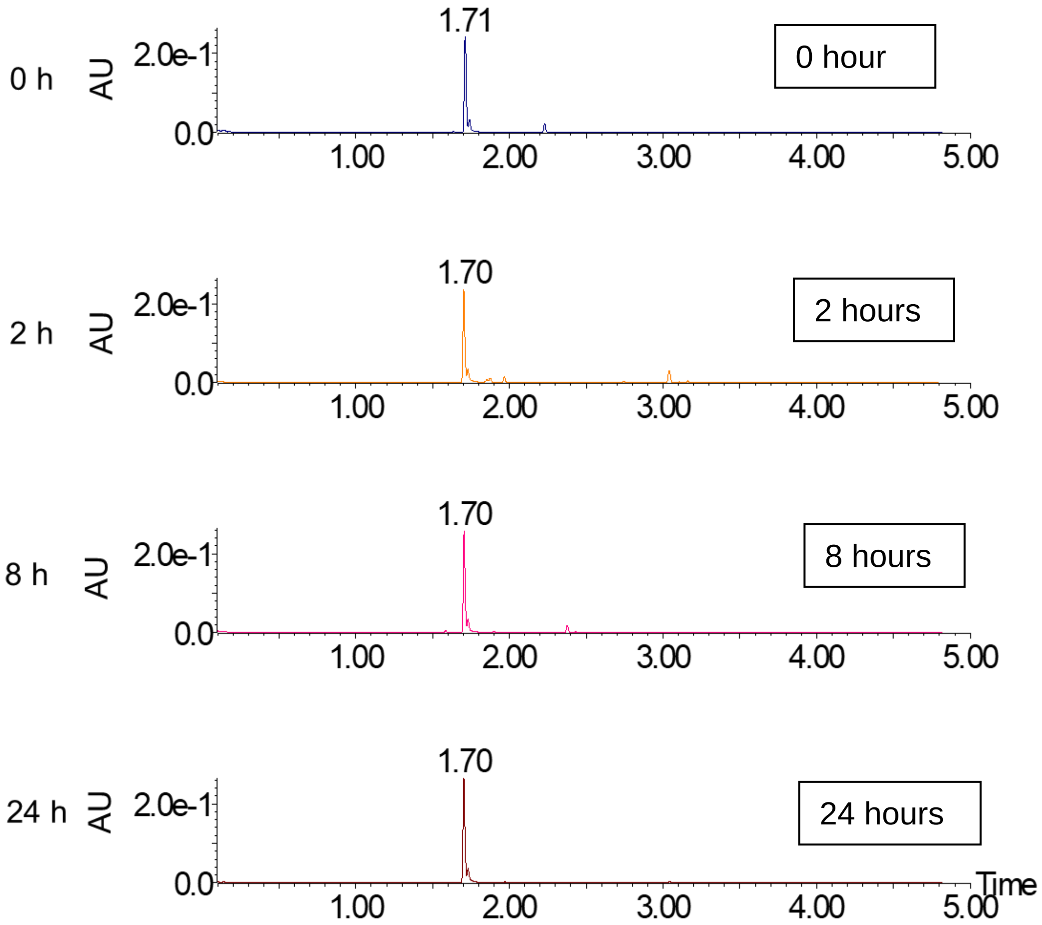

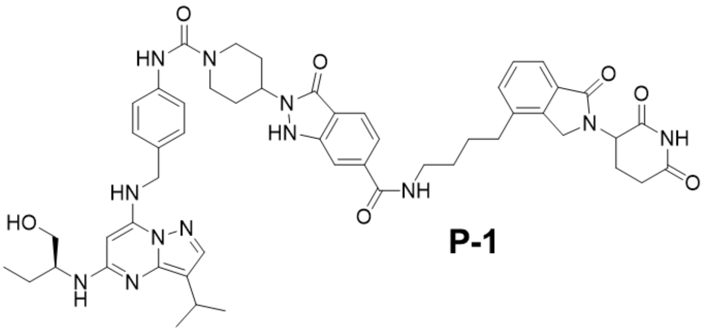

P-1

(50mM PBS)/MeOH = 1:1, pH = 7.0

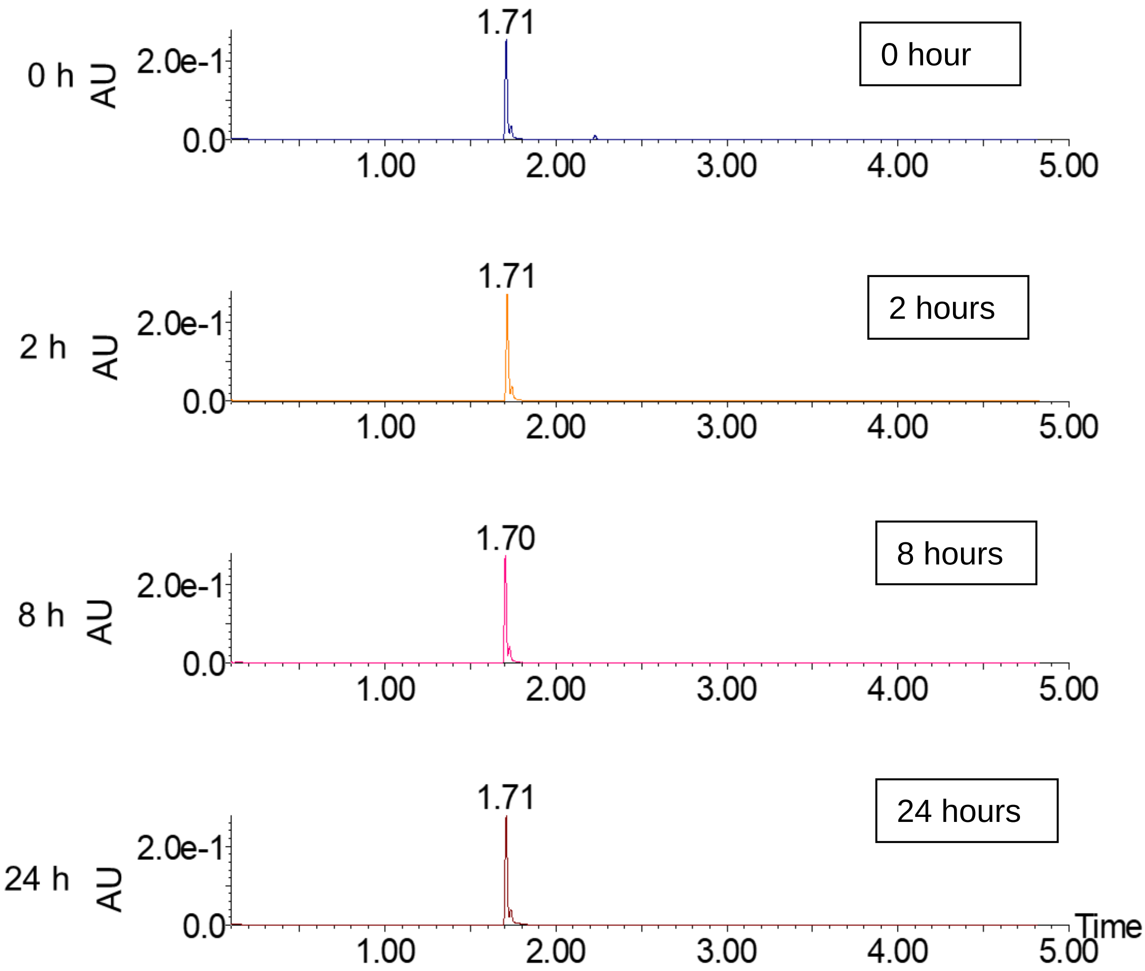

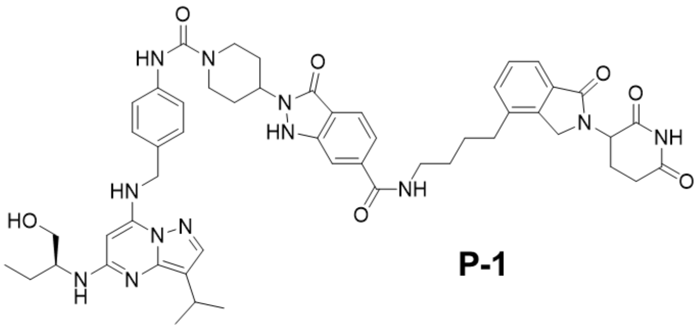

P-1

(50mM PBS)/DMSO = 1:1, pH = 10.0

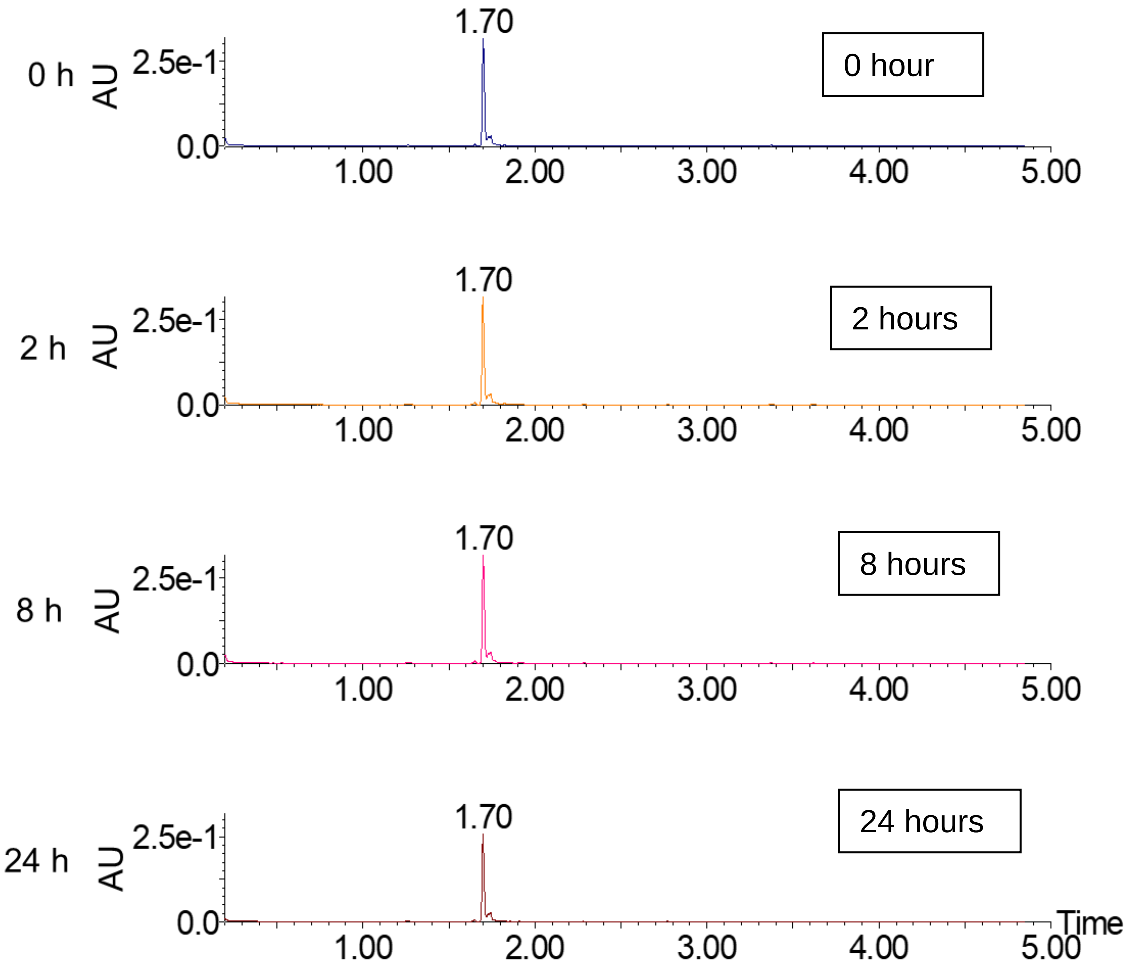

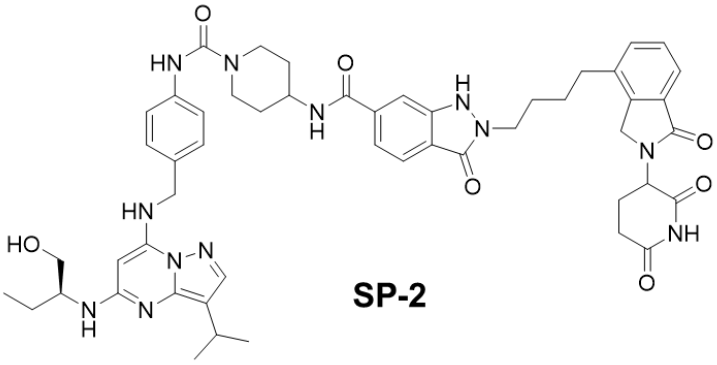

SP-2

(50mM PBS)/MeOH = 1:1, pH = 4.0

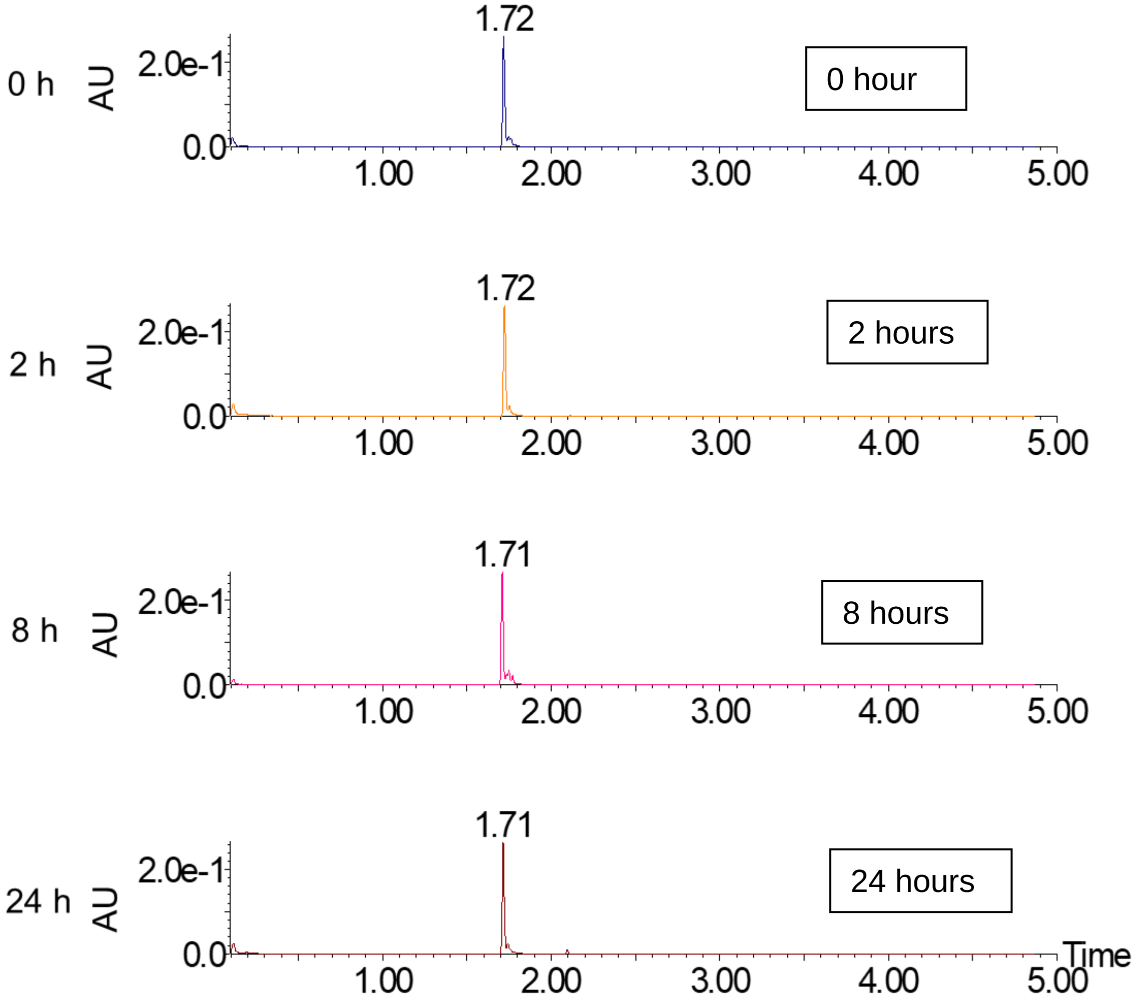

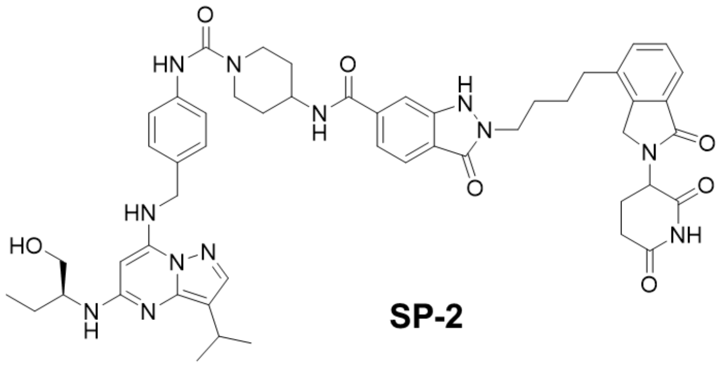

SP-2

(50mM PBS)/MeOH = 1:1, pH = 7.0

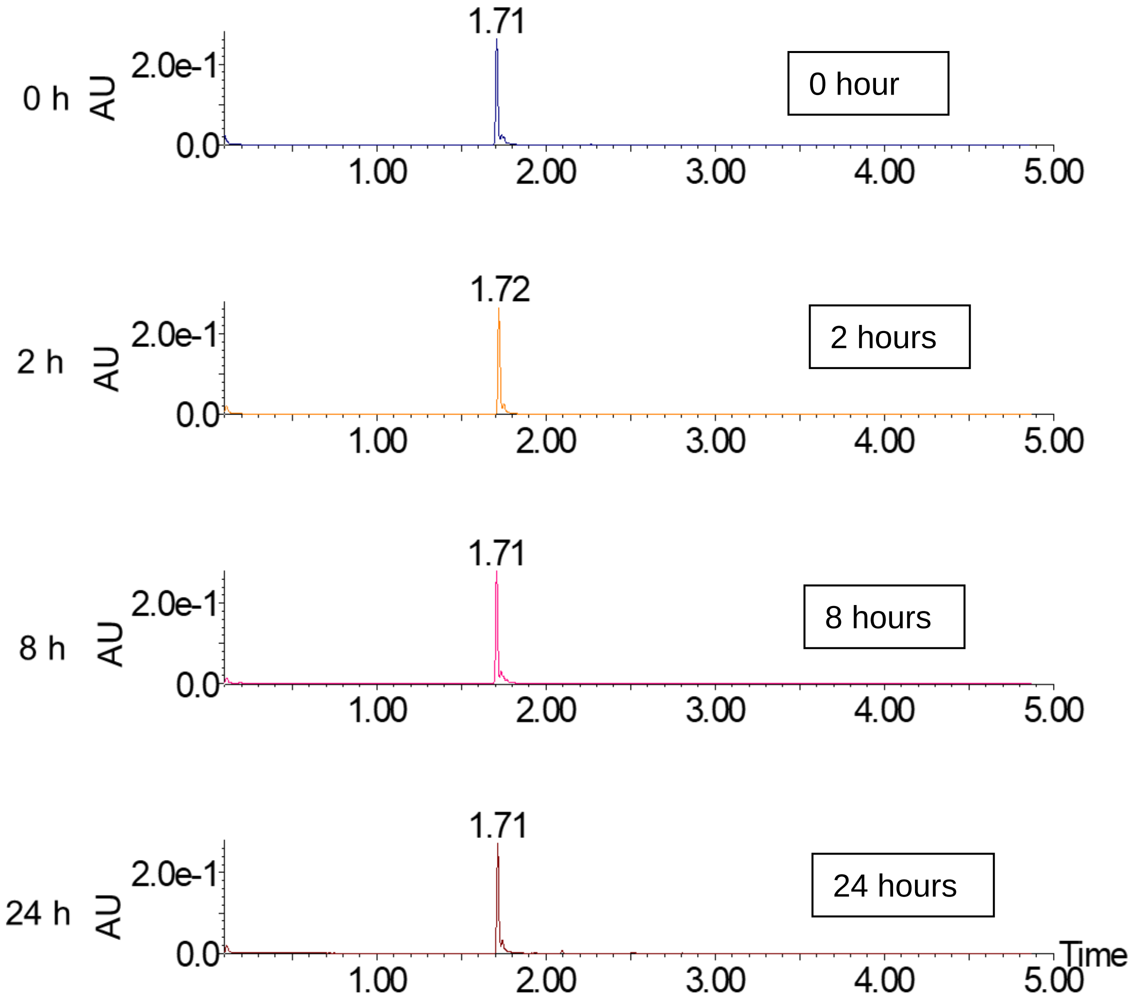

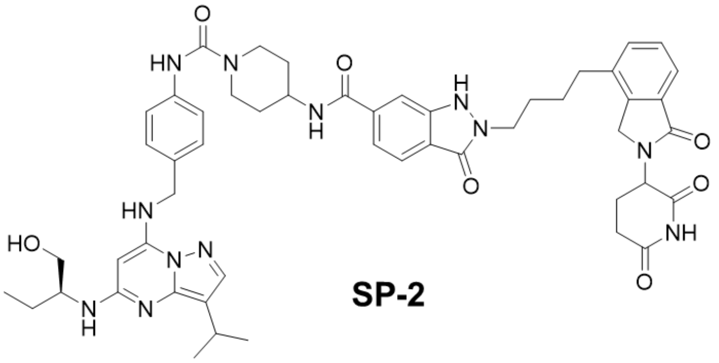

SP-2

(50mM PBS)/DMSO = 1:1, pH = 10.0

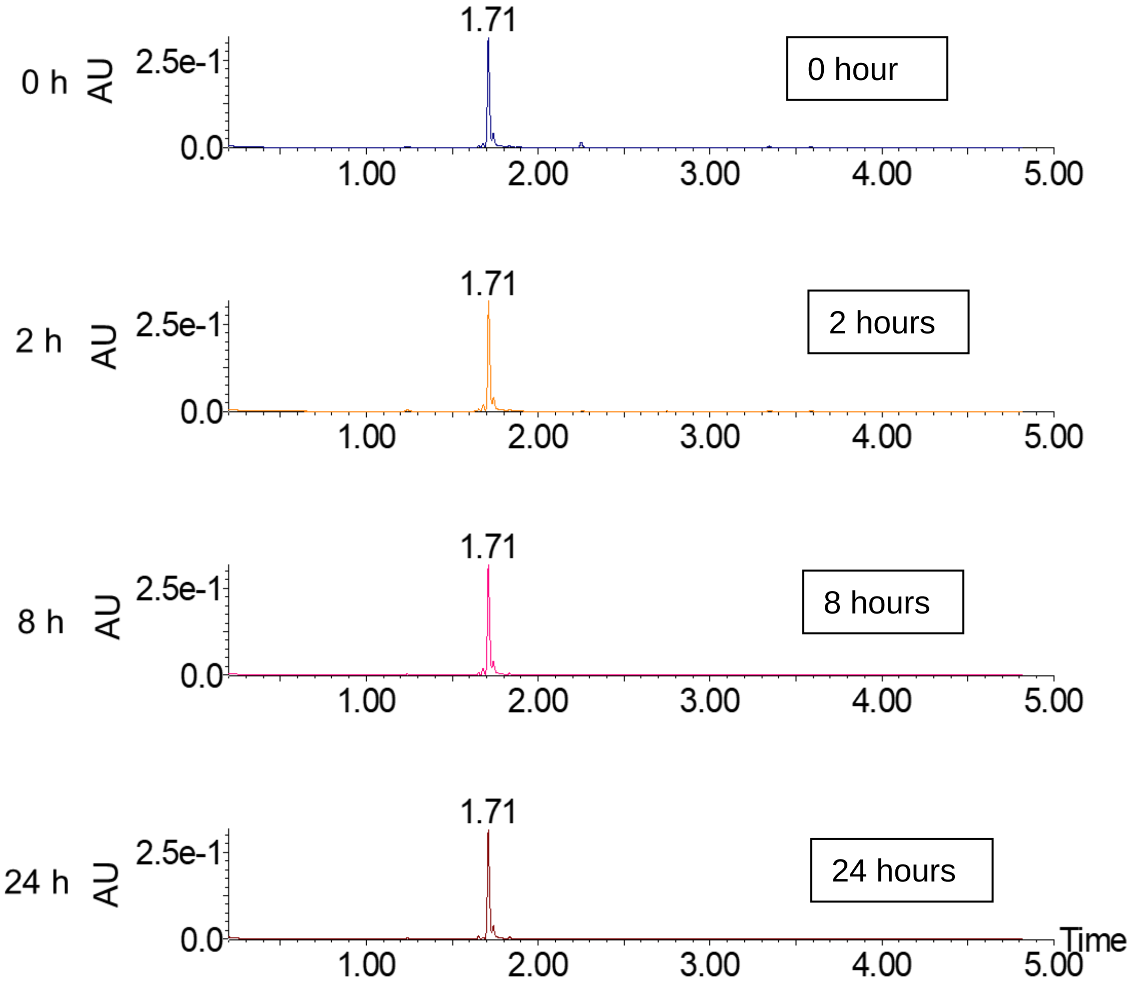

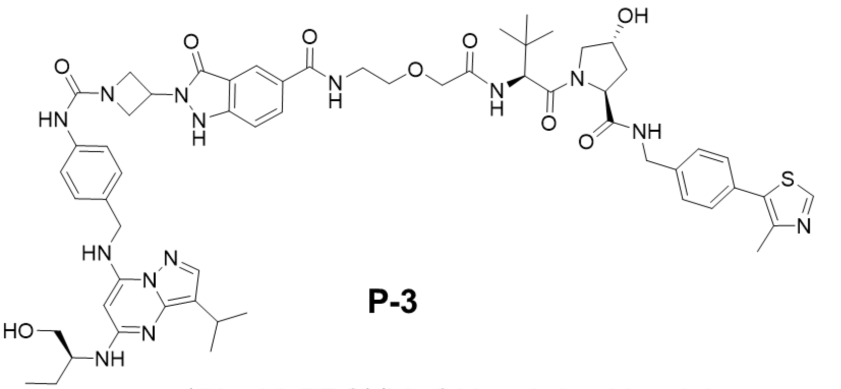

P-3

(50mM PBS)/MeOH = 1:1, pH = 4.0

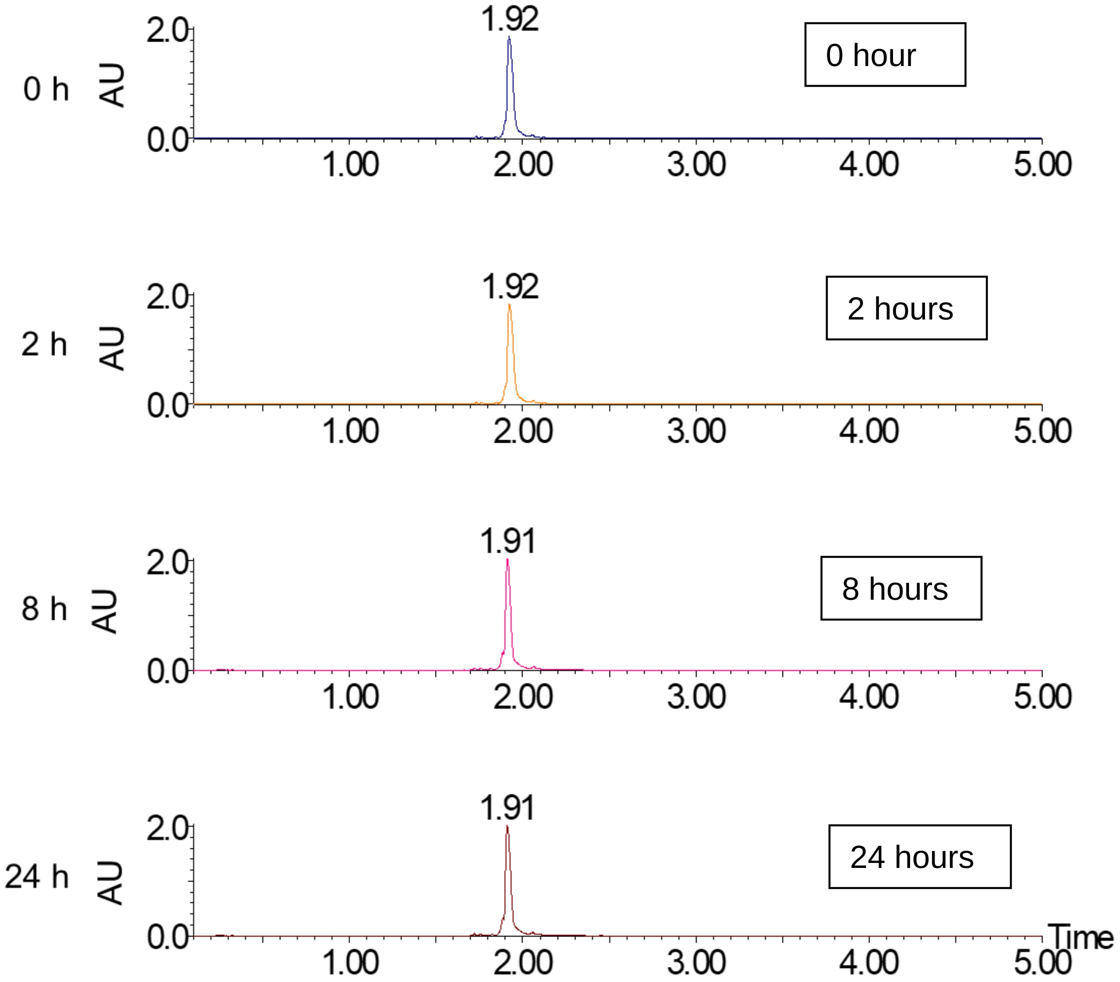

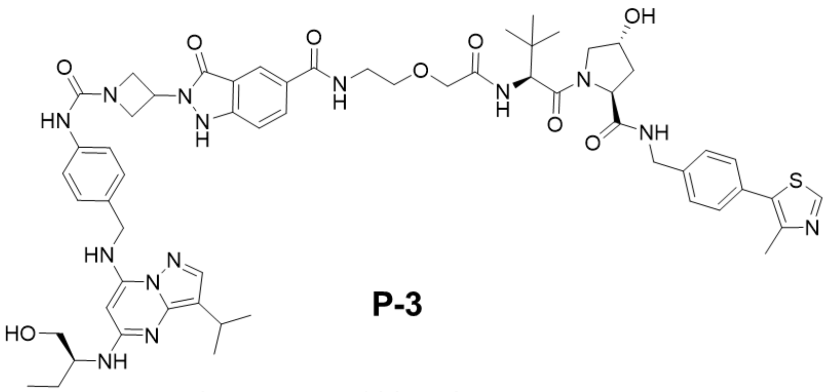

P-3

(50mM PBS)/MeOH = 1:1, pH = 7.0

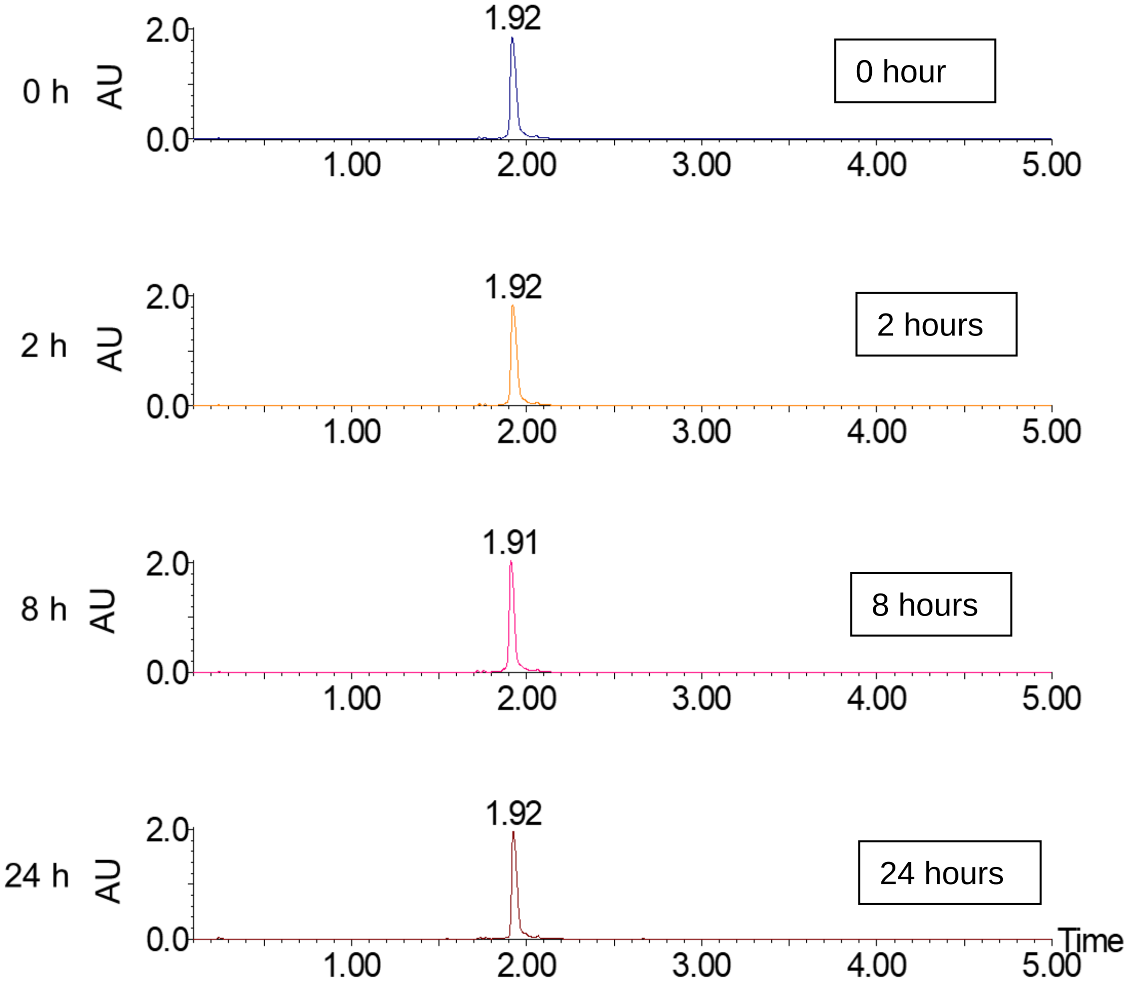

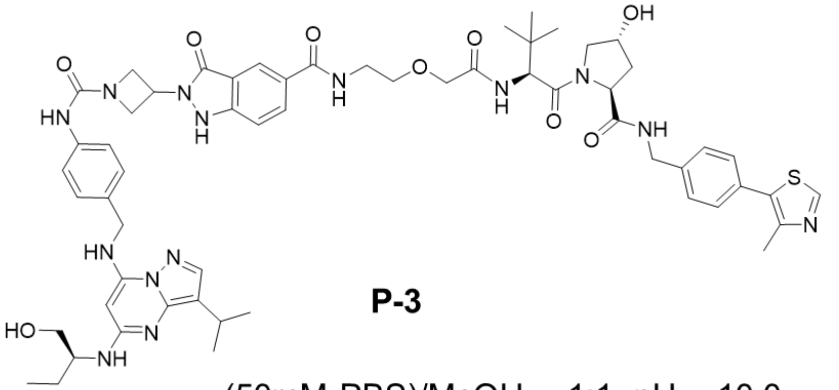

P-3

(50mM PBS)/MeOH = 1:1, pH = 10.0

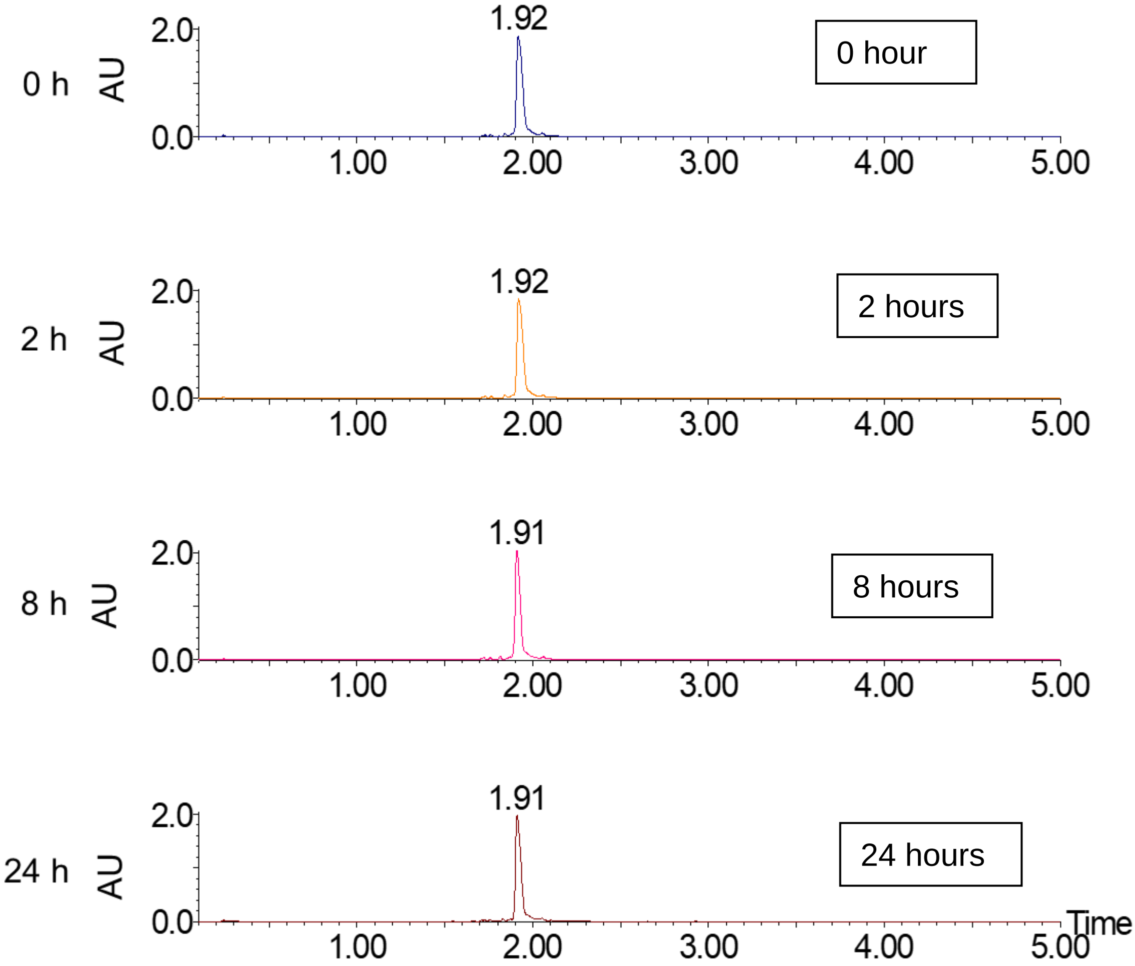

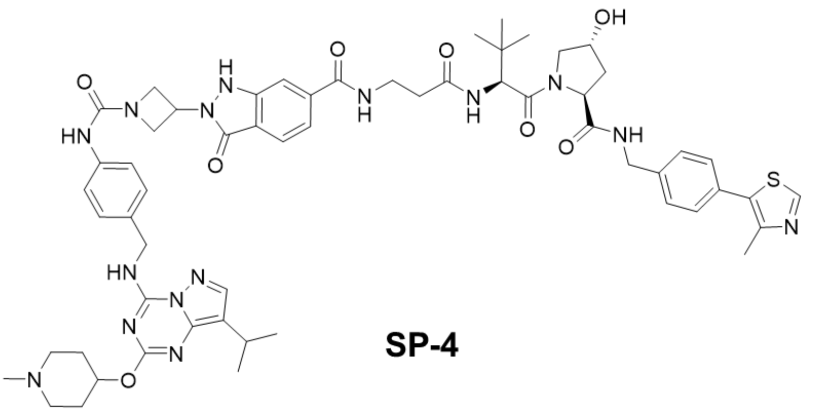

SP-4

(50mM PBS)/MeOH = 1:1, pH = 4.0

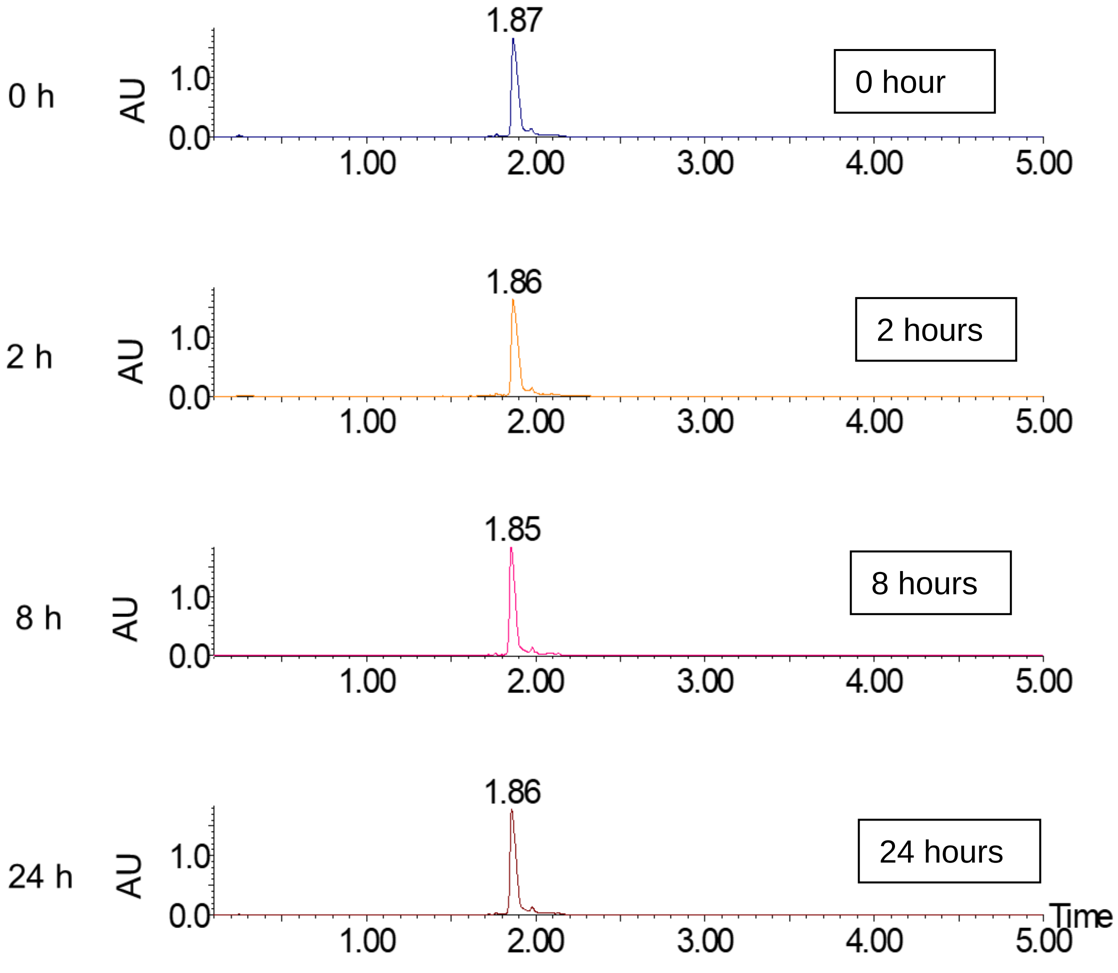

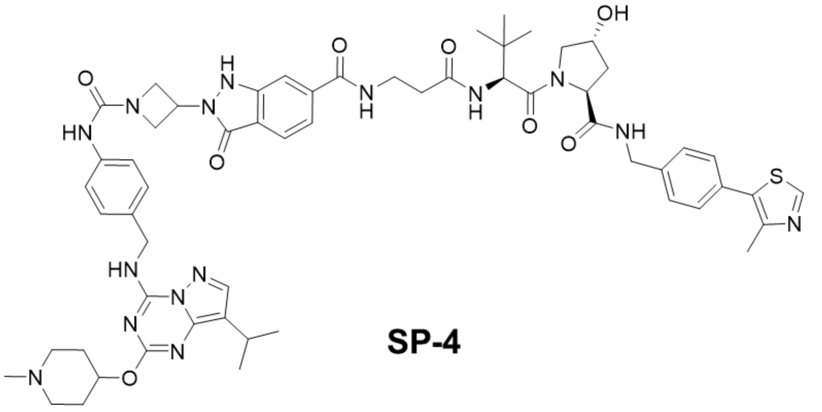

SP-4

(50mM PBS)/MeOH = 1:1, pH = 7.0

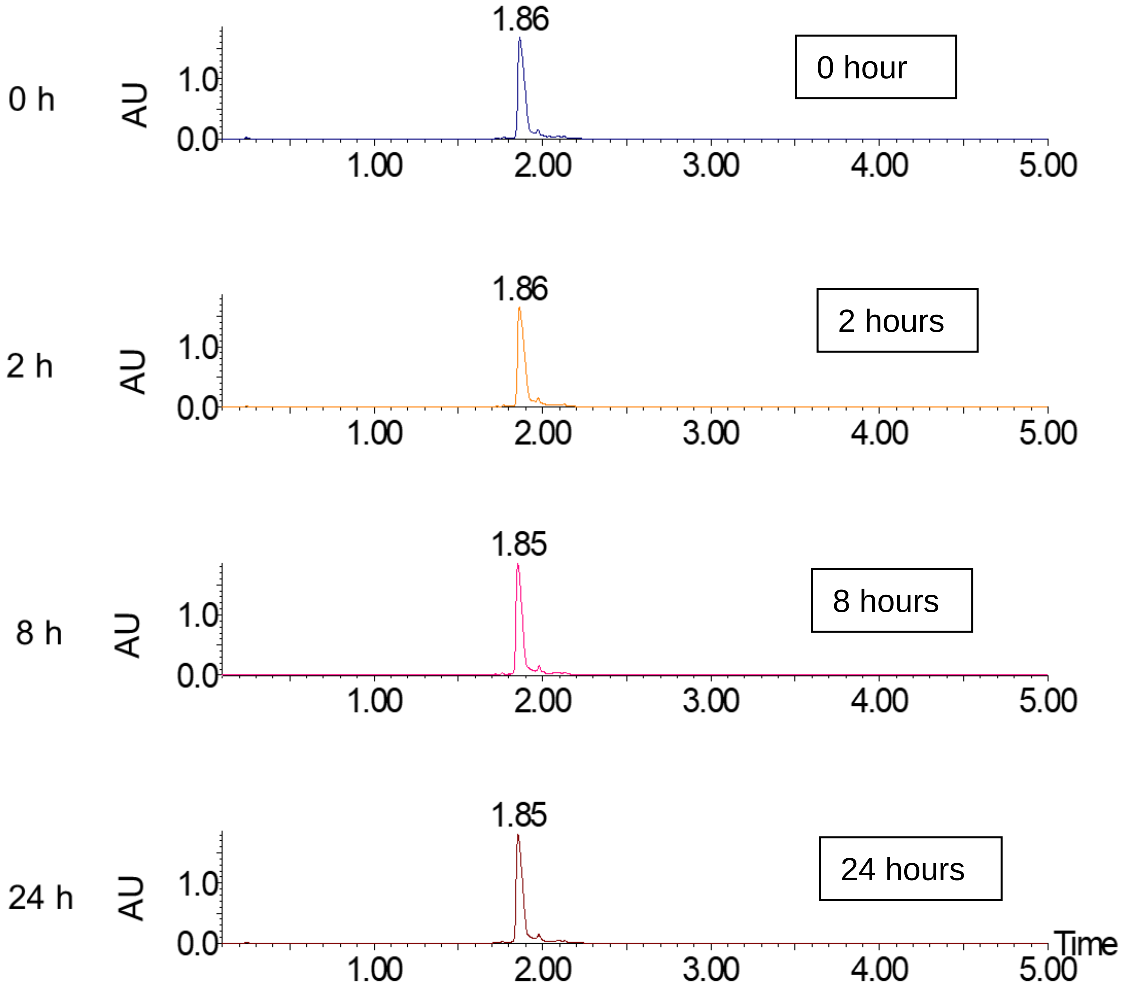

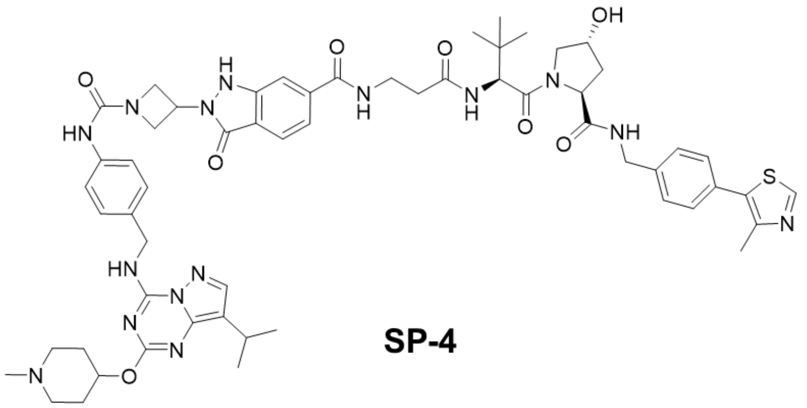

SP-4

(50mM PBS)/MeOH = 1:1, pH = 10.0

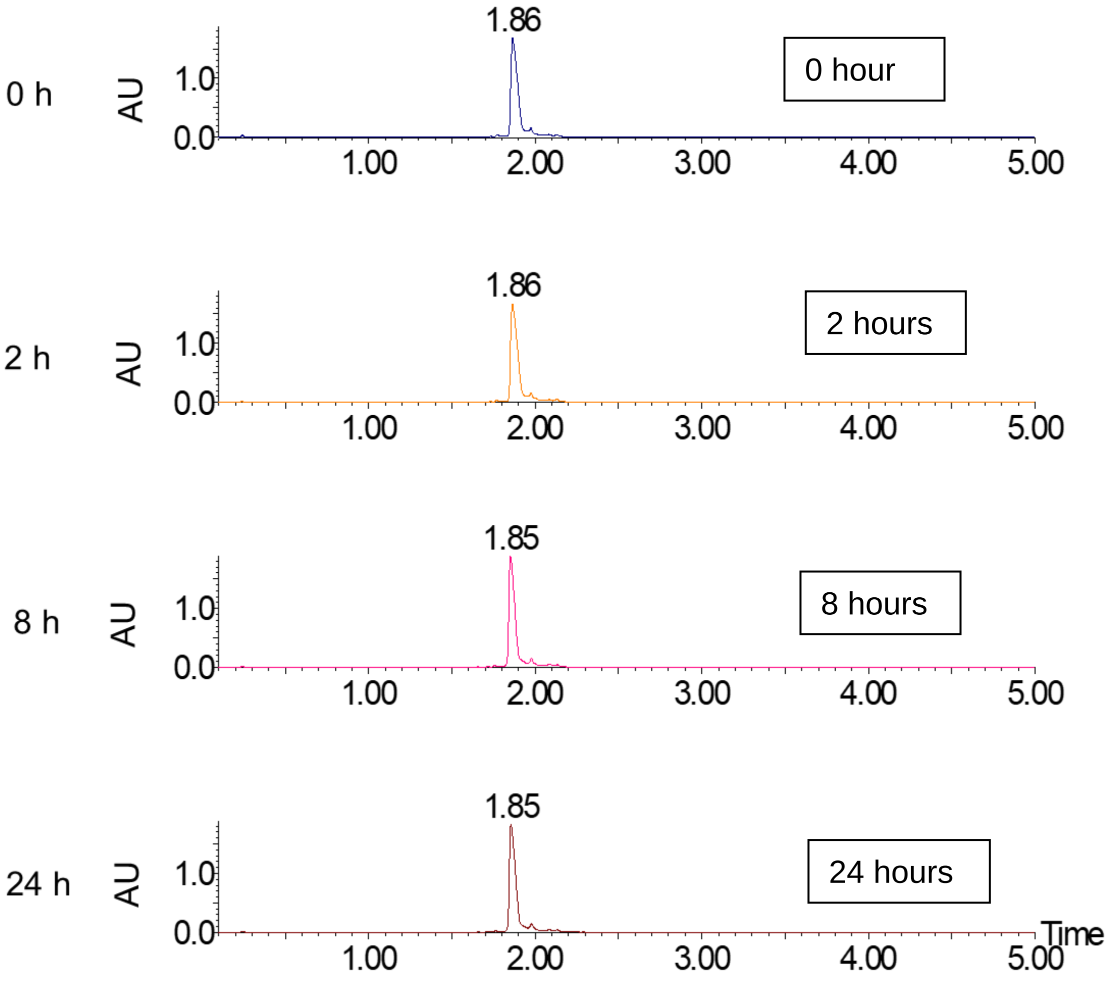

Figure S4. Stability analysis for **P-1**, **SP-2**, **P-3** and **SP-4** under different buffer pH conditions at indicated times (0, 2, 8, 24 hours.). Compounds were incubated in indicated buffer condition at room temperature, and subjected to UPLC analysis

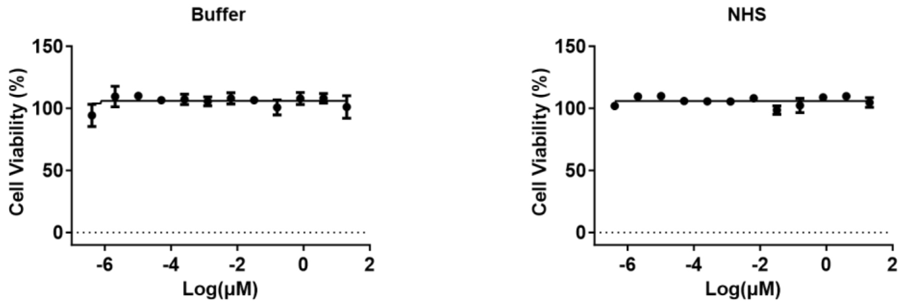

Figure S5. Cell growth inhibition curves of NHS and PBS.

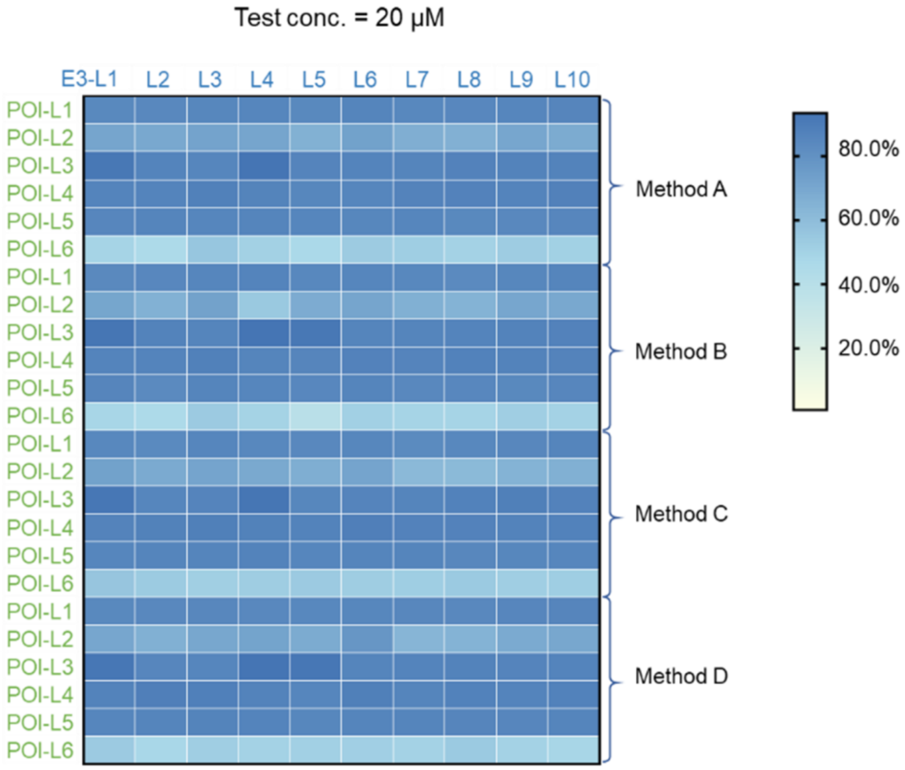

Figure S6. Heatmap of the activities of assembled PROTACs at 20 μM on TNBC MDA-MB-231 cells, heatmap colored by cell growth inhibition rate of assembled compounds corresponding to related E3 ligands and POI ligands.

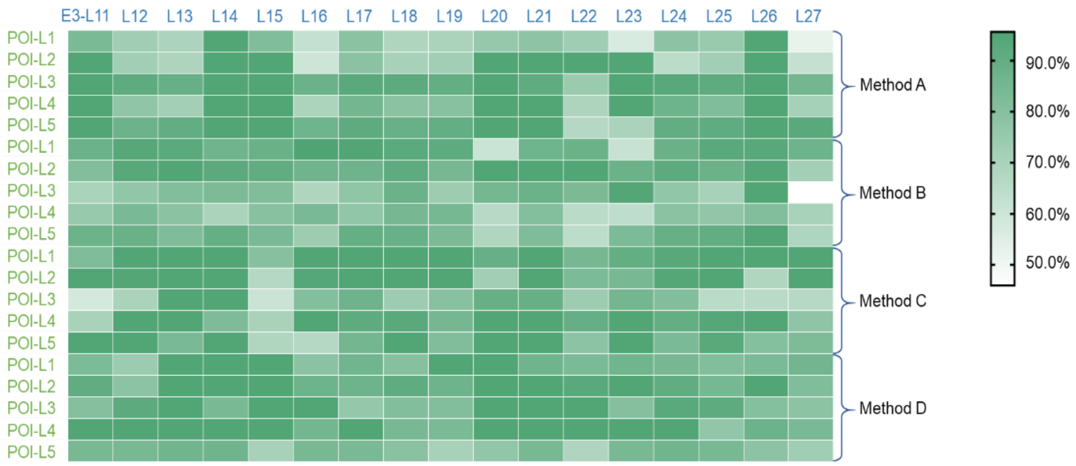

Figure S7. Heatmap of the convention of PROTACs assembly with E3 ligands (E3-L11 - E3-L27) and POI ligands (POI-L1 - POI-L5), heatmap colored by UPLC-MS conversion corresponding to related E3 ligands and POI ligands.

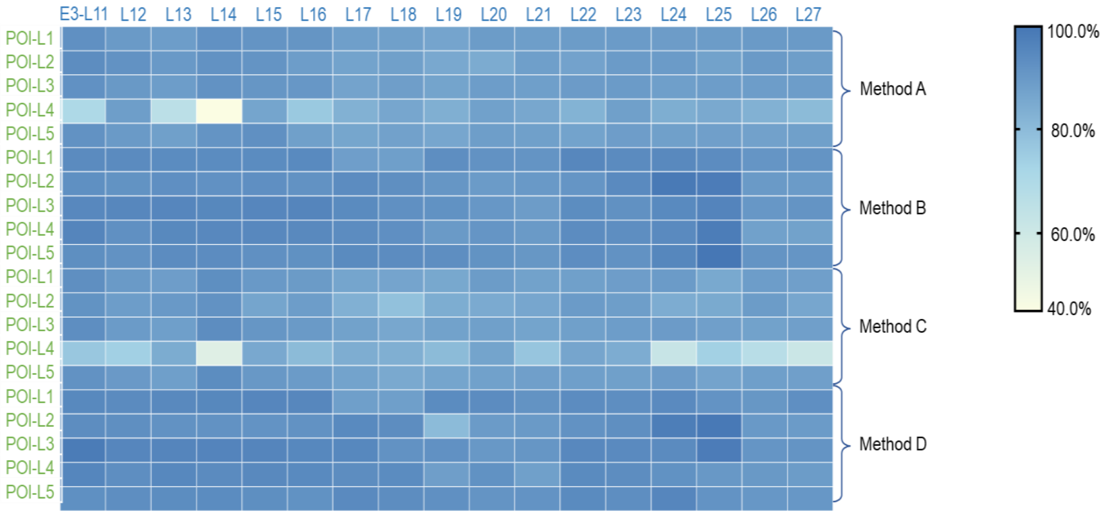

Figure S8. Heatmap of the activities of assembled PROTACs with E3 ligands (E3-L11 - E3-L27) and POI ligands (POI-L1 - POI-L5) at 20 μM on TNBC MDA-MB-231 cells, heatmap colored by cell growth inhibition rate of assembled compounds corresponding to related E3 ligands and POI ligands.

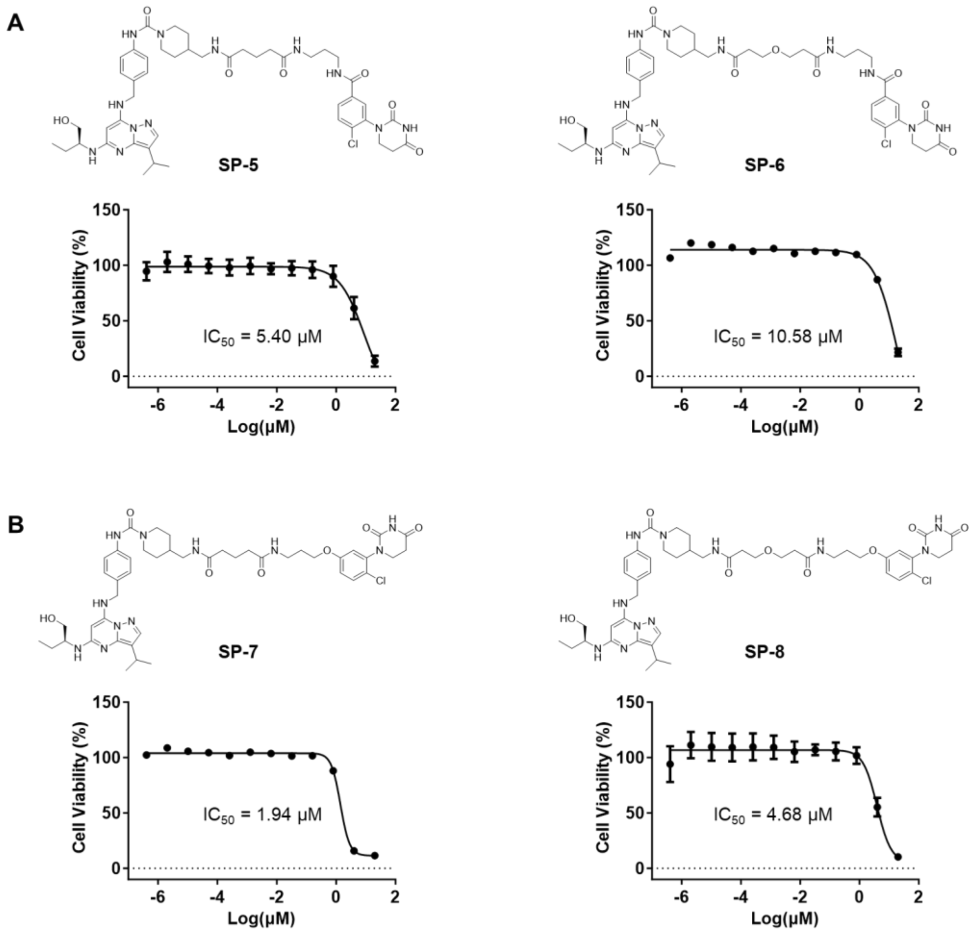

Figure S9. (A) Cell growth inhibitory activities of **SP-5 and SP-6** (carbon-chain-linker analogs of **P-4**) on MDA-MB-231 cells. (B) Cell growth inhibitory activities of **SP-7 and SP-8** (carbon-chain-liner analogs of **P-5**) on MDA-MB-231 cells.

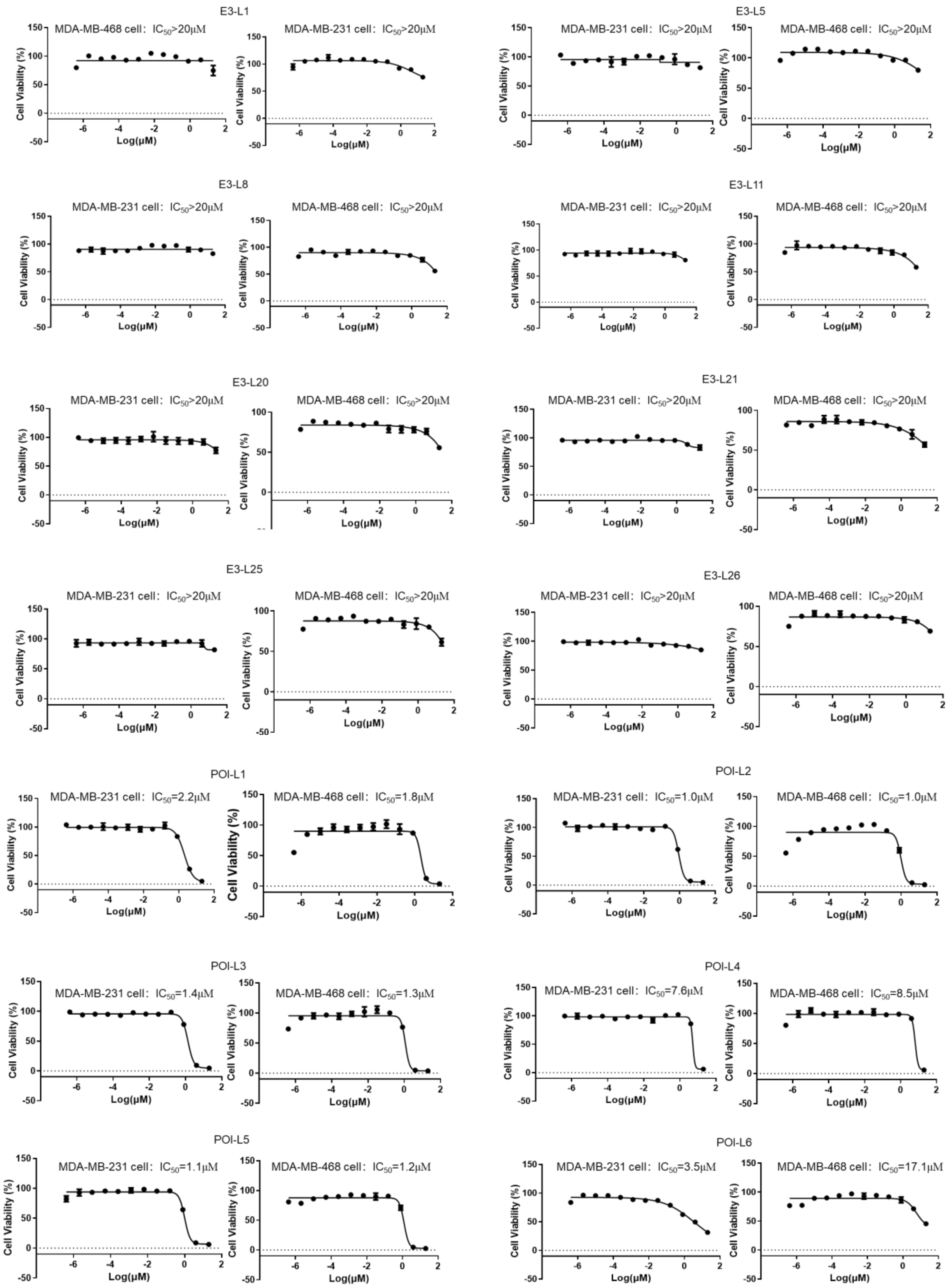

Figure S10. The growth inhibitory activities of all POI-L (POI-L1~L6) and a selection of structurally diversified E3-L (E3-L1, -L5, -L8, -L11, -L20, -L21, -L25, -L26).

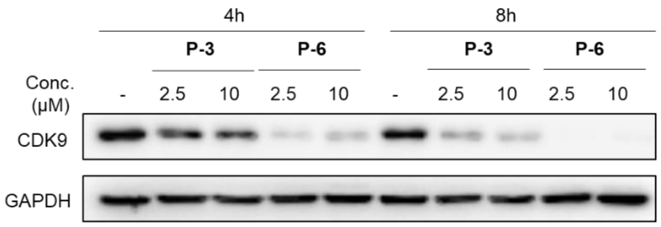

Figure S11. Immunoblot of CDK9 and GAPDH following 4 or 8 h of incubation with DMSO or the indicated concentrations of compound P-3 and P-6 in MDA-MB-231 cells.

Table S1. Conventions of the assembled PROTACs (Corresponding to Figure 3d)

Ligands	E3-L1	E3-L2	E3-L3	E3-L4	E3-L5	E3-L6	E3-L7	E3-L8	E3-L9	E3-L10	
POI-L1	95.0%	88.1%	95.0%	79.8%	93.7%	95.0%	75.0%	78.3%	86.6%	87.9%	Method A
POI-L2	95.0%	95.0%	95.0%	95.0%	92.9%	95.0%	80.3%	83.6%	83.5%	88.0%	
POI-L3	95.0%	95.0%	95.0%	77.6%	95.0%	95.0%	86.1%	83.4%	95.0%	77.9%	
POI-L4	95.0%	95.0%	95.0%	92.5%	95.0%	95.0%	85.9%	86.5%	88.4%	84.7%	
POI-L5	90.7%	95.0%	95.0%	85.5%	95.0%	95.0%	85.7%	85.9%	78.5%	95.0%	
POI-L6	91.5%	89.1%	91.4%	86.3%	88.8%	95.0%	82.2%	83.8%	77.3%	66.0%	
POI-L1	95.0%	73.5%	90.3%	80.2%	90.7%	92.9%	74.4%	77.6%	80.2%	72.1%	Method B
POI-L2	95.0%	95.0%	95.0%	95.0%	95.0%	95.0%	92.2%	79.9%	79.8%	75.5%	
POI-L3	71.1%	86.6%	80.7%	65.5%	70.6%	92.6%	69.4%	76.7%	95.0%	77.5%	
POI-L4	80.5%	81.2%	81.2%	69.2%	80.3%	84.6%	70.5%	74.3%	65.5%	61.8%	
POI-L5	78.2%	83.0%	85.7%	59.0%	55.3%	79.2%	79.4%	80.5%	82.8%	81.8%	
POI-L6	77.4%	78.0%	72.1%	72.4%	66.4%	84.4%	68.8%	63.8%	67.0%	77.7%	
POI-L1	93.7%	95.0%	87.0%	95.0%	95.0%	95.0%	95.0%	95.0%	95.0%	95.0%	Method C
POI-L2	82.9%	95.0%	95.0%	75.6%	81.6%	78.8%	94.6%	91.2%	95.0%	95.0%	
POI-L3	78.2%	84.6%	75.0%	71.3%	72.4%	95.0%	83.5%	84.9%	80.5%	95.0%	
POI-L4	83.6%	95.0%	86.7%	89.1%	77.8%	95.0%	95.0%	95.0%	89.9%	90.9%	
POI-L5	89.0%	89.0%	89.0%	68.6%	89.0%	89.0%	87.1%	84.9%	80.5%	80.4%	
POI-L6	64.8%	71.9%	74.0%	59.4%	55.8%	60.5%	68.2%	67.3%	95.0%	67.3%	
POI-L1	95.0%	83.1%	83.6%	76.5%	95.0%	95.0%	82.0%	71.6%	95.0%	95.0%	Method D
POI-L2	86.4%	87.0%	84.7%	89.3%	58.7%	94.9%	80.6%	84.7%	95.0%	95.0%	
POI-L3	76.7%	77.9%	75.6%	67.8%	74.8%	91.0%	80.3%	75.2%	77.9%	76.6%	
POI-L4	95.0%	95.0%	95.0%	88.8%	95.0%	95.0%	95.0%	95.0%	93.1%	88.6%	
POI-L5	84.1%	84.1%	79.3%	70.6%	84.1%	84.1%	75.3%	77.3%	77.1%	79.3%	
POI-L6	55.5%	60.5%	67.0%	63.5%	95.0%	61.9%	66.3%	56.5%	65.1%	69.3%	

Table S2. Cell growth inhibition rate profiles of assembled PROTACs (Corresponding to Figure 4a)

Ligands	E3-L1	E3-L2	E3-L3	E3-L4	E3-L5	E3-L6	E3-L7	E3-L8	E3-L9	E3-L10	
POI-L1	88.80%	88.14%	87.88%	87.06%	87.81%	89.45%	88.19%	88.96%	90.06%	86.96%	Method A
POI-L2	36.24%	30.25%	36.30%	35.06%	33.91%	40.20%	31.47%	39.05%	42.38%	42.02%	
POI-L3	56.45%	85.38%	63.00%	61.74%	67.20%	80.53%	80.20%	84.83%	87.81%	83.39%	
POI-L4	18.10%	27.89%	33.42%	20.67%	32.90%	27.04%	29.30%	33.51%	27.36%	33.49%	
POI-L5	75.02%	83.20%	74.86%	77.92%	73.18%	82.94%	75.76%	85.24%	85.88%	86.65%	
POI-L6	8.08%	16.02%	9.30%	10.90%	9.63%	11.35%	13.47%	9.44%	0.73%	4.99%	
POI-L1	87.80%	88.44%	89.12%	89.20%	88.96%	88.99%	89.18%	89.26%	89.62%	89.34%	Method B
POI-L2	37.36%	23.93%	41.69%	26.54%	31.27%	43.76%	33.23%	33.22%	58.72%	56.02%	
POI-L3	52.39%	83.21%	47.46%	74.70%	56.18%	75.43%	75.20%	79.32%	77.33%	68.91%	
POI-L4	18.95%	30.90%	33.17%	17.78%	21.62%	29.97%	25.62%	33.18%	32.09%	31.01%	
POI-L5	76.47%	84.60%	75.99%	77.83%	76.28%	85.63%	78.88%	85.87%	83.06%	85.04%	
POI-L6	8.01%	15.26%	9.97%	11.03%	7.31%	10.75%	16.41%	14.56%	6.20%	0.77%	
POI-L1	84.22%	87.14%	78.57%	84.34%	77.88%	86.33%	84.50%	87.60%	82.46%	76.70%	Method C
POI-L2	36.50%	30.11%	35.46%	27.40%	36.14%	40.17%	22.02%	35.53%	40.98%	34.88%	
POI-L3	85.45%	86.99%	81.93%	93.33%	77.18%	80.02%	86.79%	86.65%	86.03%	45.22%	
POI-L4	22.78%	26.67%	37.61%	19.41%	31.71%	29.09%	28.53%	29.00%	27.27%	29.95%	
POI-L5	34.34%	28.46%	35.31%	48.33%	38.84%	46.43%	35.62%	38.63%	46.38%	43.82%	
POI-L6	13.72%	20.00%	13.12%	11.48%	15.37%	14.59%	15.62%	14.00%	8.37%	9.65%	
POI-L1	81.47%	87.38%	76.31%	80.74%	78.44%	84.59%	86.72%	86.68%	76.08%	79.50%	Method D
POI-L2	32.42%	23.57%	33.65%	30.59%	31.97%	36.09%	26.87%	24.23%	46.06%	39.30%	
POI-L3	68.25%	85.19%	75.46%	84.59%	66.55%	72.01%	74.44%	84.89%	83.81%	83.38%	
POI-L4	18.50%	31.30%	33.36%	19.37%	22.25%	30.11%	28.65%	31.62%	29.96%	29.47%	
POI-L5	28.97%	29.44%	28.25%	34.81%	32.77%	38.97%	26.98%	38.82%	32.46%	38.31%	
POI-L6	10.11%	18.37%	8.03%	10.83%	10.67%	10.35%	15.55%	14.78%	6.08%	3.17%	

Table S3. Conventions of the assembled PROTACs (Corresponding to Figure 5b)

Ligands	E3-L11	E3-L12	E3-L13	E3-L14	E3-L15	E3-L16	E3-L17	E3-L18	E3-L19	E3-L20	E3-L21	E3-L22	E3-L23	E3-L24	E3-L25	E3-L26	E3-L27	
POI-L1	83.5%	72.7%	69.4%	95.0%	81.7%	62.5%	78.8%	68.5%	69.7%	75.5%	77.8%	73.0%	56.7%	78.2%	74.7%	95.0%	52.4%	
POI-L2	95.0%	72.4%	68.9%	95.0%	95.0%	60.0%	78.8%	70.5%	72.5%	95.0%	95.0%	95.0%	95.0%	65.0%	72.4%	95.0%	62.3%	
POI-L3	95.0%	91.4%	88.8%	95.0%	95.0%	87.7%	92.6%	91.8%	90.0%	95.0%	91.1%	74.3%	95.0%	93.0%	91.5%	95.0%	85.6%	Method A
POI-L4	95.0%	77.3%	71.9%	95.0%	95.0%	69.2%	85.5%	78.8%	79.6%	95.0%	95.0%	68.9%	95.0%	87.1%	81.8%	95.0%	70.9%	
POI-L5	95.0%	87.6%	90.2%	95.0%	95.0%	86.8%	91.5%	88.7%	90.1%	95.0%	95.0%	66.7%	69.7%	90.4%	90.8%	95.0%	92.7%	
POI-L1	87.7%	93.6%	92.3%	86.5%	88.2%	95.6%	94.7%	92.3%	91.4%	61.1%	86.8%	87.9%	61.7%	88.1%	92.5%	92.9%	87.1%	
POI-L2	81.3%	93.1%	95.0%	92.4%	90.7%	86.6%	86.9%	91.3%	84.3%	95.0%	95.0%	95.0%	87.8%	91.8%	87.9%	94.2%	71.8%	
POI-L3	69.8%	76.6%	81.0%	82.8%	81.5%	68.6%	77.4%	87.7%	73.5%	85.0%	87.7%	83.1%	94.2%	76.8%	70.6%	95.0%	45.9%	Method B
POI-L4	75.2%	83.6%	78.8%	69.5%	79.6%	83.6%	76.9%	84.5%	84.6%	66.2%	80.6%	65.4%	64.0%	79.1%	76.5%	81.3%	70.4%	
POI-L5	87.4%	88.2%	82.0%	89.5%	83.8%	73.7%	89.5%	88.5%	83.1%	68.1%	82.2%	64.4%	82.9%	88.9%	90.4%	95.0%	68.7%	
POI-L1	82.1%	95.0%	95.0%	95.0%	79.9%	95.0%	95.0%	95.0%	95.0%	88.8%	95.0%	84.4%	89.8%	93.1%	95.0%	95.0%	95.0%	
POI-L2	95.0%	94.2%	95.0%	95.0%	67.1%	94.0%	95.0%	95.0%	95.0%	72.5%	95.0%	83.0%	84.0%	93.9%	95.0%	68.2%	95.0%	
POI-L3	57.9%	69.5%	95.0%	95.0%	60.7%	80.7%	87.0%	73.4%	79.6%	89.0%	89.0%	73.7%	85.6%	81.2%	65.4%	65.9%	66.6%	Method C
POI-L4	70.1%	95.0%	95.0%	82.3%	70.0%	95.0%	91.5%	92.5%	84.8%	95.0%	95.0%	89.1%	95.0%	90.1%	94.1%	95.0%	77.6%	
POI-L5	95.0%	95.0%	83.6%	95.0%	68.2%	66.7%	85.4%	95.0%	81.4%	95.0%	95.0%	78.4%	95.0%	83.2%	92.8%	80.3%	82.4%	
POI-L1	83.0%	74.1%	95.0%	95.0%	95.0%	78.7%	86.1%	80.0%	95.0%	95.0%	86.5%	83.3%	86.3%	84.4%	81.2%	83.3%	85.9%	
POI-L2	90.7%	78.3%	95.0%	95.0%	95.0%	87.1%	87.2%	90.9%	87.2%	95.0%	94.9%	92.6%	95.0%	90.7%	84.6%	95.0%	81.7%	
POI-L3	80.4%	91.9%	95.0%	83.9%	95.0%	95.0%	75.8%	81.0%	81.1%	95.0%	95.0%	95.0%	80.4%	92.6%	91.4%	80.3%	78.5%	Method D
POI-L4	95.0%	95.0%	95.0%	95.0%	95.0%	85.6%	95.0%	84.1%	83.5%	95.0%	95.0%	95.0%	95.0%	95.0%	77.0%	87.6%	83.6%	
POI-L5	84.1%	84.1%	86.1%	86.5%	70.8%	84.1%	83.6%	79.3%	70.6%	76.9%	84.1%	68.4%	84.1%	86.3%	84.1%	78.2%	72.6%	

Table S4.Cell growth inhibition rate profiles of assembled PROTACs (Corresponding to Figure 5c)

Ligands	E3-L11	E3-L12	E3-L13	E3-L14	E3-L15	E3-L16	E3-L17	E3-L18	E3-L19	E3-L20	E3-L21	E3-L22	E3-L23	E3-L24	E3-L25	E3-L26	E3-L27	
POI-L1	46.82%	58.56%	38.06%	57.56%	51.32%	50.29%	47.26%	40.58%	37.47%	39.36%	68.31%	49.26%	57.21%	59.79%	41.25%	53.46%	40.17%	
POI-L2	88.56%	37.68%	16.71%	12.93%	14.25%	8.89%	7.43%	6.91%	10.58%	4.93%	12.34%	20.42%	22.89%	38.28%	23.74%	6.11%	34.69%	
POI-L3	23.40%	18.64%	15.50%	33.27%	43.32%	10.67%	8.03%	14.62%	11.18%	7.34%	14.30%	23.66%	46.11%	11.39%	26.82%	16.04%	16.83%	Method A
POI-L4	10.43%	9.18%	2.14%	1.18%	1.67%	2.98%	8.81%	4.63%	6.31%	4.63%	4.92%	4.65%	4.56%	1.94%	17.97%	6.03%	4.43%	
POI-L5	81.29%	79.54%	72.45%	72.93%	80.02%	82.58%	68.13%	68.99%	71.37%	65.24%	68.12%	61.44%	61.66%	70.85%	75.27%	57.13%	69.96%	
POI-L1	92.49%	91.07%	92.08%	91.61%	85.09%	84.59%	16.30%	59.92%	35.77%	50.75%	44.91%	69.88%	60.98%	61.46%	61.71%	32.08%	36.27%	
POI-L2	91.04%	90.24%	91.48%	91.24%	91.32%	91.23%	64.81%	61.51%	58.34%	75.36%	86.78%	89.79%	82.40%	82.16%	87.40%	85.79%	76.67%	
POI-L3	0.51%	11.61%	26.22%	54.98%	27.68%	25.56%	75.93%	66.27%	49.39%	86.58%	83.29%	87.53%	86.15%	90.41%	82.66%	74.27%	61.69%	Method B
POI-L4	14.00%	14.01%	13.27%	15.65%	15.76%	12.69%	20.28%	17.75%	2.29%	21.53%	32.13%	14.99%	7.82%	19.90%	21.75%	12.12%	13.83%	
POI-L5	72.94%	81.69%	83.22%	80.10%	84.29%	80.94%	87.51%	86.95%	63.94%	87.71%	89.14%	80.16%	79.08%	83.27%	92.73%	64.35%	46.14%	
POI-L1	40.22%	53.75%	36.34%	56.43%	51.75%	38.96%	56.44%	65.87%	36.26%	84.89%	45.70%	70.19%	31.76%	49.37%	33.69%	14.88%	30.72%	
POI-L2	15.66%	5.69%	12.29%	19.31%	1.87%	3.72%	14.30%	24.06%	7.93%	12.79%	9.53%	26.67%	10.97%	11.36%	14.05%	9.60%	19.19%	
POI-L3	29.98%	25.49%	24.50%	27.11%	55.95%	17.52%	10.24%	11.49%	19.71%	37.65%	16.82%	44.68%	15.11%	25.72%	16.07%	15.11%	16.97%	Method C
POI-L4	0.91%	3.19%	1.40%	13.07%	9.07%	5.08%	6.43%	4.13%	4.02%	18.05%	11.36%	2.89%	15.77%	3.02%	8.48%	12.08%	6.61%	
POI-L5	66.60%	71.01%	76.41%	81.60%	80.73%	73.81%	71.03%	71.36%	73.29%	67.44%	62.45%	76.16%	59.97%	66.47%	69.82%	54.46%	77.62%	
POI-L1	92.20%	91.77%	91.80%	91.91%	86.22%	85.41%	19.30%	68.86%	24.72%	48.31%	30.60%	45.78%	30.96%	44.86%	39.07%	28.80%	24.06%	
POI-L2	75.25%	77.43%	73.01%	71.01%	84.91%	73.02%	58.71%	58.15%	50.63%	59.28%	61.65%	76.91%	75.19%	79.73%	77.12%	53.59%	89.16%	
POI-L3	4.23%	6.86%	23.61%	30.52%	15.35%	14.43%	34.44%	17.51%	18.65%	29.96%	39.27%	78.16%	79.29%	79.09%	48.84%	14.45%	18.06%	Method D
POI-L4	14.90%	15.30%	17.08%	14.02%	15.55%	12.42%	19.87%	22.88%	0.36%	39.35%	21.82%	10.53%	11.56%	15.15%	21.80%	11.61%	13.07%	
POI-L5	58.00%	58.98%	67.12%	58.27%	61.79%	60.59%	41.70%	44.22%	20.56%	62.70%	71.59%	44.02%	36.51%	46.81%	57.10%	21.24%	16.86%	

Table S5. The IC_{50} (μM) values of P-4 ~ P-6 on BT549, MM.1S and MV-4-11 cells.

PROTACs	BT549[a]	MM.1S[a]	MV-4-11[a]
**P-4**	0.190±0.062	0.321±0.056	0.197±0.004
**P-5**	1.400±0.236	0.531±0.096	0.336±0.066
**P-6**	1.628±0.275	0.978±0.079	0.317±0.011

[a] mean ± S.D.

Methods

1.1 General information

O-(7-Azabenzotriazol-1-yl)-N,N,N′,N′-tetramethyluronium hexafluorophosphate (HATU), N-(3-dimethylaminopropyl)-N'-ethylcarbodiimide hydrochloride (EDCI) and 1-Hydroxybenzotriazole (HOBt) were purchased from Bidepharm. Diisopropylethylamine (DIEPA), N,N-dimethylformamide (DMF) were purchased from Sinopharm. HCl-dioxane solution was purchased from EnergyChemical. Formic acid (FA), acetonitrile (ACN) were purchased from Sigma. The human TNBC cells MDA-MB-231 and MDA-MB-468 used in this paper were all purchased from Cell Bank of Type Culture Collection, Chinese Academy of Sciences (Shanghai, China).0.25% Trypsin-EDTA (Cat.25200-072),penicillin-streptomycin (Cat. 15070063) and fetal bovine serum (Cat. 10099141) were purchased from Gibco.Leibovitz’s L-15 Medium (Cat.L620KJ) were purchased from BasalMedia.Cell Counting Kit-8 was purchased from DOJINDO. Phosphate buffered saline (Cat.MA0015) were purchased from meiilunbio.BCA protein assay kit (Cat.23227),SuperSignal West Pico PLUS Chemiluminescent Substrate (Cat.34577) and Prestained Protein Ladder (Cat.26616) were purchased from Thermo Scientific.Glycine (Cat.62011519),Albumin from bovine serum (Cat.69003435),Phenylmethanesulfonyl fluoride (PMSF),NaCl (Cat.10019318),Tween 20 (Cat.30189328),Sodium dodecyl sulfate (Cat.30166428),TEMED (Cat.80125336),Isopropanol (Cat.40064360),Methanol (Cat.10014159),Ethanol absoulte (Cat.10009218),Hydrochloric acid (Cat.10011018) and Tris (Cat.30188360) were purchased from Sinopharm.Skim Milk (Cat.36120ES76) and 5×SDS-PAGE Protein Loading Buffer (Cat.20315ES) were purchased from Yeasen Biotechnology (Shanghai) Co.,Ltd.Acryl/Bis 30% Soultion (Cat.B546017-0500) was purchased from Sangon Biotech.GAPDH Mouse Monoclonal antibody (Cat.60004-1),CDK1-Specific Polyclonal antibody (Cat.19532-1-AP) and HRP-conjugated Affinipure Goat Anti-Mouse IgG(H+L) (Cat.SA00001-1) were purchased from Proteintech.CDK2 (78B2) Rabbit mAb (Cat.2546S),CDK4 (D9G3E) Rabbit mAb (Cat.12790S),CDK5 (D1F7M) Rabbit mAb (Cat.14145S),CDK6 (D4S8S) Rabbit mAb (Cat.13331S),CDK7 (MO1) Mouse mAb (Cat.2916S),CDK9 (C12F7) Rabbit mAb (Cat.2316S),beta-Actin (13E5) Rabbit mAb(HRP Conjugate) (Cat.5125S) and Anti-rabbit IgG, HRP-linked Antibody (Cat.7074S) were purchased from Cell Signaling Technology.Protease Inhibitor Cocktail Tablets (Cat.04693159001) were purchased from Roche.Dimethyl

sulfoxide was purchased from Sigma-Aldrich. RIPA Lysis Buffer (Cat.P0013B) was purchased from Beyotime.LC-MS analysis was obtained on a Waters UPLC Acquity-H. ^{1}HNMR spectra and ^{13}CNMR spectra were obtained on a Bruker AVANCE III 500 (500 MHz) NMR spectrometer. Chemical shifts were reported in parts per million (ppm) on the δ scale from an internal standard (NMR descriptions: s, singlet; d, doublet; t, triplet; q, quartet; m, multiplet; br, broad). Coupling constants, J, are reported in Hertz. For HRMS analysis, samples were analyzed by flow-injection analysis into an Agilent 6520 UHPLC-QTOF. Light-induced reactions were conducted with ZF-7A 16 W 365 nm UV as light source.

1.2 Methods of light-induced reactions in plates and LC-MS analysis.

1.2.1 Wellplate-based methods for two-step PROTACs assembly

Reaction buffer: (20mM PBS)/DMSO=1:1, pH=10.5. **Method A**: 2μL stock of E3 ligand (100 mM) was mixed with equal volume of o-NBA-NHS-2 stock (100 mM) in 46μL buffer followed by shaking for 20min. Next, to the reaction mixture, 2μL stock of POI ligand (100 mM) and 48μL buffer was added (final concentration of all reactants, 2mM). Then mixture was then exposed to 365nm UV for 10min and further incubated for 5min. Repeat the UV-irradiation and incubation once. The reaction crude was directly used in inhibition rate test or diluted with ACN/H_2O=1:1 solution for LC-MS analysis.

Method B: 2μL stock of E3 ligand (100 mM) was mixed with equal volume of o-NBA-NHS-1 stock (100 mM) in 46μL buffer followed by shaking for 20min. Next, to the reaction mixture, 2μL stock of POI ligand (100 mM) and 48μL buffer was added (final concentration of all reactants, 2mM). Then mixture was then exposed to 365nm UV for 10min and further incubated for 5min. Repeat the UV-irradiation and incubation once. The reaction crude was directly used in inhibition rate test or diluted with ACN/H_2O=1:1 solution for LC-MS analysis.

Method C: 2μL stock of POI ligand (100 mM) was mixed with equal volume of o-NBA-NHS-2 stock (100 mM) in 46μL buffer followed by shaking for 20min. Next, to the reaction mixture, 2μL stock of E3 ligand (100 mM) and 48μL buffer was added (final concentration of all reactants, 2mM). Then mixture was then exposed to 365nm UV for 10min and further incubated for 5min. Repeat the UV-irradiation and incubation once. The reaction crude was directly used in inhibition rate test or diluted with ACN/H_2O=1:1

solution for LC-MS analysis.

Method D: 2μL stock of POI ligand (100 mM) was mixed with equal volume of o-NBA-NHS-1 stock (100 mM) in 46μL buffer followed by shaking for 20min. Next, to the reaction mixture, 2μL stock of E3 ligand (100 mM) and 48μL buffer was added (final concentration of all reactants, 2mM). Then mixture was then exposed to 365nm UV for 10min and further incubated for 5min. Repeat the UV-irradiation and incubation once. The reaction crude was directly used in inhibition rate test or diluted with ACN/H_2O=1:1 solution for LC-MS analysis.

1.2.2 Wellplate-based single-step reaction for validating the conditions in PROTACs assembly (seen in Figure 2a/b)

Method 1: 2μL stock of E3 or POI ligand (100 mM) was mixed with equal volume of o-NBA-NHS-1 or o-NBA-NHS-2 stock (100 mM) in 46μL buffer to obtain the concertation of 4mM. The reaction crude was stirred for 20min and diluted with ACN/H_2O=1:1 solution for LC-MS analysis.

Method 2: 2μL stock of oNBA linked E3 or POI ligand (100 mM) was mixed with 2μL of POI or E3 stock (100 mM) in 96μL buffer to obtain the concertation of 2mM. The mixture was exposed to 365nm UV for 10min and further incubated for 5min. Repeat the UV-irradiation and incubation once. The reaction crude was diluted with ACN/H_2O=1:1 solution for LC-MS analysis.

LC-MS methods and conversion rate calculation: LCMS analysis was performed on Waters UPLC-MS（ESI）with ACQUITY UPLC BEH C18 1.7 μm column (UPLC: Waters HPLC H-CLASS, MS: Waters SQ Detector 2), using Pump A: Water, 0.01% formic acid; Pump B: Acetonitrile, 0.01% formic acid.

Three methods were used in this work:

Time	Flow (mL/min)	Pump A	Pump B
0.00	0.50	95%	5%
3.00	0.50	5%	95%
4.00	0.50	5%	95%
4.10	0.50	95%	5%
5.00	0.50	95%	5%

Time	Flow (mL/min)	Pump A	Pump B
0.00	0.50	95%	5%
4.00	0.50	40%	60%
4.20	0.50	5%	95%
4.50	0.50	95%	5%
5.00	0.50	95%	5%

Time	Flow (mL/min)	Pump A	Pump B
0.00	0.50	95%	5%
0.50	0.50	70%	30%
3.50	0.50	40%	60%
3.80	0.50	5%	95%
4.20	0.50	95%	5%
5.00	0.50	95%	5%

Conversion rate was calculated as follows:

For **Method A and B**, calculate the difference between the peak area of POI ligand (POIL) standard ($S_{POIL\ standard}$) and the peak area of the rest of POIL in the mixture after the reaction ($S_{the\ rest\ of\ POIL}$), and then dividing this difference by $S_{POIL\ standard}$. (Eq 1)

Eq 1 $$Conversion\ rate\ (\%) \ = (\frac{S_{(POIL\ standard)} - S_{(the\ rest\ of\ POIL)}}{S_{(POIL\ standard)}}) \times 100\%$$

For **Method C and D**, calculate the difference between the peak area of the standard of the POIL-oNBA conjugate ($S_{POIL\text{-}oNBA\ standard}$) and the peak area of the rest of POIL in the mixture after the reaction ($S_{the\ rest\ of\ POIL\text{-}oNBA}$), and then dividing this difference by $S_{POIL\text{-}oNBA\ standard.}$ (Eq 2)

Eq 2 $$Conversion\ rate\ (\%) \ = (\frac{S_{(POIL-oNBA\ standard)} - S_{(the\ rest\ of\ POIL-oNBA)}}{S_{(POIL-oNBA\ standard)}}) \times 100\%$$

1.3 Methods of cellular experiment

Cellular Inhibition Rate Assay: The human TNBC cells MDA-MB-231 and MDA-MB-468 used in this work were all purchased from Cell Bank of Type Culture Collection, Chinese Academy of Sciences (Shanghai, China). MDA-MB-231 and MDA-MB-468 cells were cultured in Leibovitz's (L-15) media supplemented with 10% FBS and 1% penicillin–streptomycin antibiotics in an incubator without CO_2 at 37 ℃. For the cell viability assay, 3×10^4 MDA-MB-231 cells and 3×10^4 MDA-MB-468 cells of 80 μL cell suspension were plated in 96-well cell culture plates. The tested compounds were gradient-diluted with the corresponding media, 20 μL of the diluted compound was added to each well, and then cells were cultured at 37 ℃ for another 4 days. The cell viability was measured by the Cell Counting Kit-8 reagent (DOJINDO, Cat. CK04) according to the manufacturer's instructions. The Inhibition curve was generated by WPS Office software.

Cellular viability Assay: 3×10^4 MDA-MB-231 cells and 3×10^4 MDA-MB-468 cells of 90 μL cell suspension were plated in 96-well cell culture plates. The tested compounds were gradient-diluted with the corresponding media, 10 μL of the diluted compound was added to each well, and then cells were cultured at 37 ℃ for another 4 days. The cell viability was measured by the Cell Counting Kit-8 reagent (DOJINDO, Cat. CK04) according to the manufacturer's instructions. The viability curve was generated by GraphPad Prism 5.0 software.

Western Blotting Assay.: Cells were lysed with cold RIPA lysis buffer (Beyotime, P0013B) containing the complete protease inhibitor cocktail (ROCHE) and 1 mM PMSF for 30 min on ice before insoluble debris

was pelleted by centrifugation at 4 ℃ at 12,000g for 10 min. The protein concentration was determined by a BCA Protein Assay Kit (Thermo Fisher Scientific, 23225), and the absorbance at 562 nm was measured by spectrophotometry (Tecan Spark 10M). The samples were separated by 10% sodium dodecyl sulfate-PAGE using 10−20 μg of protein per well and transferred to 0.2 μm PVDF membrane (Bio-Rad). After blocking with TBS containing 0.1% Tween-20 (TBS-T) with 5% skimmed milk, the membrane was incubated with primary antibodies. The membranes were washed three times for 5 min with TBS-T before incubating with HRP-conjugated secondary antibodies in TBS-T with 5% milk for 1 h at room temperature. Then, the membranes were washed three times for 5 min with TBS-T. Immunodetection was performed using the SuperSignal West Pico PLUS Chemiluminescent Substrate (Thermo Fisher Scientific, 34577).

1.4 Synthesis methods and NMR data

1.4.1 E3 Ligands used in this work

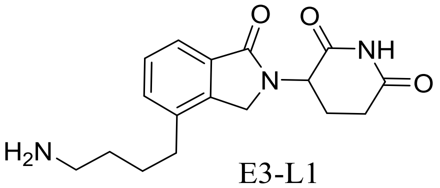

E3-L1

3-(4-(4-aminobutyl)-1-oxoisoindolin-2-yl)piperidine-2,6-dione was synthesized according to literature procedures[1]. ^{1}H NMR (500 MHz, DMSO-d6) δ 11.00 (s, 1H), 8.03 (s, 3H, HCl salt), 7.58 (dd, *J* = 6.3, 2.2 Hz, 1H), 7.50 – 7.44 (m, 2H), 5.14 (dd, *J* = 13.3, 5.1 Hz, 1H), 4.50 (d, *J* = 17.2 Hz, 1H), 4.32 (d, *J* = 17.1 Hz, 1H), 2.93 (ddd, *J* = 17.4, 13.7, 5.4 Hz, 1H), 2.80 (t, *J* = 7.2 Hz, 2H), 2.66 (t, *J* = 7.4 Hz, 2H), 2.63 – 2.57 (m, 1H), 2.43 (qd, *J* = 13.3, 4.4 Hz, 1H), 2.02 (ddd, *J* = 10.2, 5.2, 3.0 Hz, 1H), 1.70 – 1.58 (m, 4H). ^{13}C NMR (126 MHz, DMSO-d6) δ 172.95, 171.08, 140.60, 137.00, 131.58, 128.32, 125.31, 120.79, 51.55, 48.61, 46.27, 31.22, 30.74, 26.86, 26.07, 22.61. LRMS (ESI): m/z $[M+H]^+$ Calcd for $C_{17}H_{22}N_3O_3^+$: 316.16; found: 316.35.

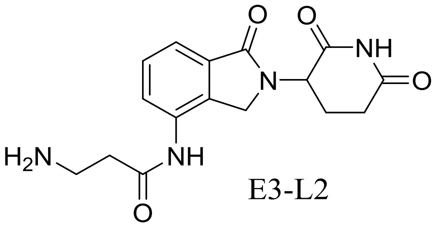

E3-L2

3-amino-N-(2-(2,6-dioxopiperidin-3-yl)-1-oxoisoindolin-4-yl)propanamide was synthesized according to literature procedures[2]. ^{1}H NMR (500 MHz, DMSO-d6) δ 11.04 (s, 1H), 10.12 (s, 1H), 7.85 (dd, *J* = 7.4, 1.3 Hz, 4H, HCl salt), 7.57 – 7.48 (m, 2H), 5.17 (dd, *J* = 13.3, 5.1 Hz, 1H), 4.42 (d, *J* = 17.5 Hz, 1H), 4.34 (d, *J* = 17.5 Hz, 1H), 3.16 – 3.05 (m, 2H), 2.97 – 2.89 (m, 1H), 2.76 (t, *J* = 6.8 Hz, 2H), 2.65 – 2.59 (m, 1H), 2.32 (qd, *J* = 13.2, 4.3 Hz, 1H), 2.04 (ddd, *J* = 10.1, 5.0, 2.9 Hz, 1H). ^{13}C NMR (126 MHz, DMSO-d6) δ 172.93, 171.13,

168.58, 167.83, 133.73, 133.40, 132.77, 132.51, 128.73, 125.30, 119.34, 51.52, 46.43, 34.96, 32.87, 31.25, 22.73. LRMS (ESI): m/z $[M+H]^+$ Calcd for $C_{16}H_{19}N_4O_4^+$: 331.13; found: 331.29.

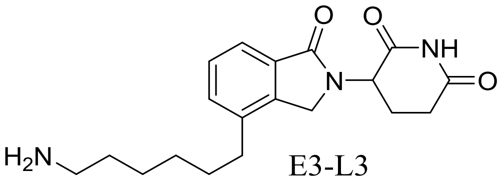

E3-L3

3-(4-(6-aminohexyl)-1-oxoisoindolin-2-yl)piperidine-2,6-dione was synthesized according to literature procedures[1]. ^{1}H NMR (500 MHz, DMSO-d6) δ 11.01 (s, 1H), 7.94 (s, 3H, HCl salt), 7.57 (p, *J* = 3.8 Hz, 1H), 7.51 – 7.40 (m, 2H), 5.14 (dd, *J* = 13.3, 5.1 Hz, 1H), 4.47 (d, *J* = 17.2 Hz, 1H), 4.30 (d, *J* = 17.1 Hz, 1H), 2.93 (ddd, *J* = 17.4, 13.7, 5.4 Hz, 1H), 2.78 – 2.69 (m, 2H), 2.62 (ddd, *J* = 17.6, 9.8, 4.8 Hz, 3H), 2.43 (tt, *J* = 13.4, 6.6 Hz, 1H), 2.01 (ddd, *J* = 10.2, 5.1, 3.0 Hz, 1H), 1.65 – 1.49 (m, 4H), 1.40 – 1.27 (m, 4H). ^{13}C NMR (126 MHz, DMSO-d6) δ 173.40, 171.57, 168.83, 140.98, 137.91, 132.03, 131.94, 128.76, 121.11, 52.01, 49.06, 46.71, 31.67, 31.55, 29.54, 28.80, 27.32, 26.06, 23.00. LRMS (ESI): m/z $[M+H]^+$ Calcd for $C_{19}H_{26}N_3O_3^+$: 344.19; found: 344.33.

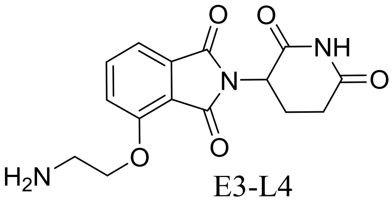

E3-L4

4-(2-aminoethoxy)-2-(2,6-dioxopiperidin-3-yl)isoindoline-1,3-dione was synthesized according to literature procedures[3]. ^{1}H NMR (500 MHz, CD_3OD) δ 7.85 (t, *J* = 7.9 Hz, 1H), 7.57 (d, *J* = 7.2 Hz, 1H), 7.52 (d, *J* = 8.4 Hz, 1H), 5.16 (dd, *J* = 12.7, 5.3 Hz, 1H), 4.53 – 4.43 (m, 2H), 3.46 (d, *J* = 4.1 Hz, 2H), 2.92 – 2.84 (m, 1H), 2.73 (ddd, *J* = 26.7, 15.5, 11.2 Hz, 2H), 2.18 – 2.11 (m, 1H). ^{13}C NMR (126 MHz, CD_3OD) δ 174.53, 174.00, 171.41, 168.57, 168.15, 156.70, 138.50, 135.10, 121.26, 117.95, 67.49, 50.60, 40.02, 32.12, 23.61. LRMS (ESI): m/z $[M+H]^+$ Calcd for $C_{15}H_{16}N_3O_5^+$: 318.10; found: 318.25.

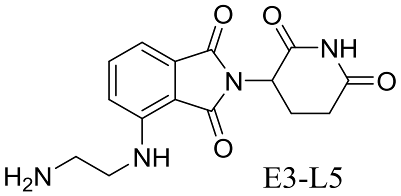

E3-L5

4-((2-aminoethyl)amino)-2-(2,6-dioxopiperidin-3-yl)isoindoline-1,3-dione was synthesized according to literature procedures[4]. ^{1}H NMR (500 MHz, DMSO-d6) δ 11.11 (s, 1H), 7.81 (s, 3H, HCl salt), 7.62 (dd, *J* = 8.4, 7.2 Hz, 1H), 7.18 (d, *J* = 8.6 Hz, 1H), 7.10 (d, *J* = 7.0 Hz, 1H), 6.84 (t, *J* = 6.3 Hz, 1H), 5.07 (dd, *J* = 12.8, 5.4 Hz, 1H), 3.57 (q, *J* = 6.1 Hz, 2H), 3.00 (dd, *J* = 11.8, 5.9 Hz, 2H), 2.90 (ddd, *J* = 16.9, 13.9, 5.4 Hz, 1H), 2.65 – 2.56 (m, 1H), 2.56 – 2.51 (m, 1H), 2.02 (ddd, *J* = 7.4, 5.3, 2.7 Hz, 1H). ^{13}C NMR (126 MHz, DMSO-d6) δ 172.84, 170.09, 168.63, 167.26, 145.79, 136.32, 132.31, 117.16, 111.08, 110.09, 48.57, 37.83, 30.98, 22.18. LRMS (ESI): m/z $[M+H]^+$ Calcd for $C_{15}H_{17}N_4O_4^+$: 317.12; found: 317.29.

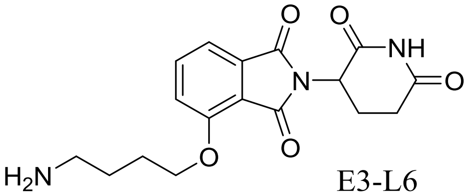

E3-L6

4-(4-aminobutoxy)-2-(2,6-dioxopiperidin-3-yl)isoindoline-1,3-dione was synthesized according to literature procedures[5]. ^{1}H NMR (500 MHz, DMSO-d6) δ 11.13 (s, 1H), 7.92 (s, 3H, HCl salt), 7.81 (dd, *J* = 8.4, 7.4 Hz, 1H), 7.51 (d, *J* = 8.6 Hz, 1H), 7.45 (d, *J* = 7.2 Hz, 1H), 5.08 (dd, *J* = 12.8, 5.4 Hz, 1H), 4.23 (t, *J* = 5.9 Hz, 2H), 2.89 (ddd, *J* = 17.1, 13.8, 5.2 Hz, 3H), 2.63 – 2.57 (m, 1H), 2.54 (dd, *J* = 13.0, 4.4 Hz, 1H), 2.03 (ddd, *J* = 10.3, 5.3, 3.0 Hz, 1H), 1.84 (dt, *J* = 12.5, 6.3 Hz, 2H), 1.80 – 1.72 (m, 2H). ^{13}C NMR (126 MHz, DMSO-d6) δ 172.98, 170.11, 166.95, 165.52, 155.91, 137.21, 133.29, 119.83, 116.28, 115.41, 68.45, 48.84, 38.67, 31.05, 25.49, 24.05, 22.12. LRMS (ESI): m/z $[M+H]^+$ Calcd for $C_{17}H_{20}N_3O_5^+$: 346.13; found: 346.31.

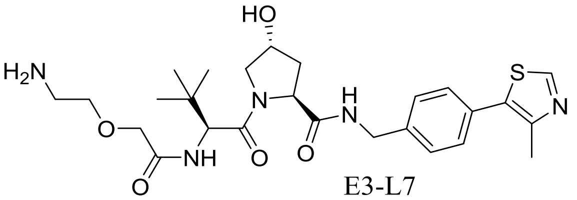

E3-L7

(2S,4R)-1-((S)-2-(2-(2-aminoethoxy)acetamido)-3,3-dimethylbutanoyl)-4-hydroxy-N-(4-(4-methylthiazol-5-yl)benzyl)pyrrolidine-2-carboxamide was synthesized according to literature procedures[6]. ^{1}H NMR (500 MHz, CD_3OD) δ 9.01 (s, 1H), 7.45 (dd, *J* = 22.4, 6.8 Hz, 4H), 4.73 (s, 1H), 4.59 – 4.50 (m, 3H), 4.35 (d, *J* = 15.5 Hz, 1H), 4.16 (d, *J* = 15.1 Hz, 1H), 4.09 (d, *J* = 15.2 Hz, 1H), 3.90 (d, *J* = 11.0 Hz, 1H), 3.84 – 3.73 (m, 3H), 3.19 (s, 2H), 2.49 (s, 3H), 2.25 (dd, *J* = 12.9, 7.6 Hz, 1H), 2.12 – 2.05 (m, 1H), 1.06 (s, 9H). ^{13}C NMR (126 MHz, CD_3OD) δ 174.27, 172.08, 171.52, 153.30, 148.32, 140.55, 133.82, 131.16, 130.58, 130.38, 129.54, 129.03, 71.04, 70.60, 68.45, 60.91, 58.53, 58.22, 43.67, 40.42, 39.05, 36.94, 26.91, 15.50. LRMS (ESI): m/z $[M+H]^+$ Calcd for $C_{26}H_{38}N_5O_5S^+$: 532.25; found: 532.46.

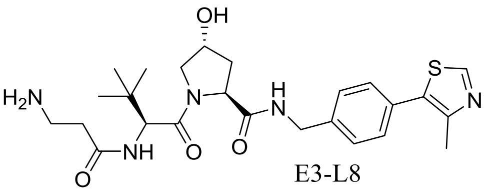

E3-L8

(2S,4R)-1-((S)-2-(3-aminopropanamido)-3,3-dimethylbutanoyl)-4-hydroxy-N-(4-(4-methylthiazol-5-yl)benzyl)pyrrolidine-2-carboxamide was synthesized according to literature procedures[6]. ^{1}H NMR (500 MHz, CD_3OD) δ 9.94 (s, 1H), 7.57 (d, *J* = 8.1 Hz, 2H), 7.52 (d, *J* = 8.2 Hz, 2H), 4.61 – 4.50 (m, 4H), 4.40 (d, *J* = 15.8 Hz, 1H), 3.99 (d, *J* = 11.0 Hz, 1H), 3.80 (dd, *J* = 10.9, 3.8 Hz, 1H), 3.18 (dt, *J* = 12.4, 6.3 Hz, 2H), 2.70 (t, *J* = 6.4 Hz, 2H), 2.60 (s, 3H), 2.25 (dd, *J* = 13.2, 7.5 Hz, 1H), 2.12 – 2.05 (m, 1H), 1.06 (s, 9H). ^{13}C NMR (126 MHz, CD_3OD) δ 174.55, 172.36, 172.16, 156.45, 142.53, 130.45, 129.43, 71.19, 68.13, 60.80, 59.45, 57.90, 43.59, 39.16, 37.00, 36.23, 32.33, 27.00, 13.17. LRMS (ESI): m/z $[M+H]^+$ Calcd for $C_{25}H_{36}N_5O_4S^+$: 502.24; found: 502.40.

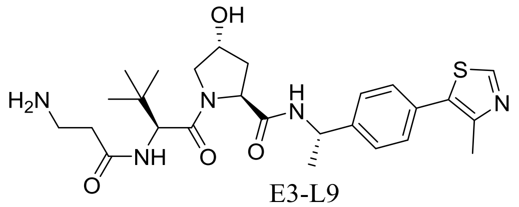

E3-L9

(2S,4R)-1-((S)-2-(3-aminopropanamido)-3,3-dimethylbutanoyl)-4-hydroxy-N-((S)-1-(4-(4-methylthiazol-5-yl)phenyl)ethyl)pyrrolidine-2-carboxamide was synthesized according to literature procedures[6]. ^{1}H NMR (500 MHz, CD_3OD) δ 8.89 (s, 1H), 7.56 (d, *J* = 8.2 Hz, 2H), 7.39 (d, *J* = 8.2 Hz, 2H), 5.02 – 4.96 (m, 1H), 4.62 (t, *J* = 8.3 Hz, 1H), 4.53 (d, *J* = 6.8 Hz, 2H), 3.97 (d, *J* = 11.0 Hz, 1H), 3.76 (dd, *J* = 10.9, 3.8 Hz, 1H), 3.22 – 3.13 (m, 2H), 2.69 (t, *J* = 6.4 Hz, 2H), 2.47 (s, 3H), 2.24 (dd, *J* = 13.1, 7.6 Hz, 1H), 2.11 (ddd, *J* = 13.3, 9.2, 4.4 Hz, 1H), 1.50 (d, *J* = 7.0 Hz, 3H), 0.99 (s, 9H). ^{13}C NMR (126 MHz, CD_3OD) δ 170.88, 170.78, 151.49, 147.41, 143.88, 132.19, 129.69, 128.82, 126.51, 69.88, 59.31, 58.14, 56.47, 37.59, 35.56, 34.83, 31.00, 25.59, 20.85, 14.42. LRMS (ESI): m/z $[M+H]^+$ Calcd for $C_{26}H_{38}N_5O_4S^+$: 516.26; found: 516.40.

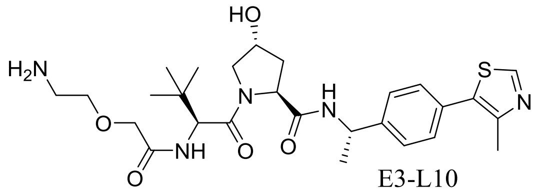

E3-L10

(2S,4R)-1-((S)-2-(2-(2-aminoethoxy)acetamido)-3,3-dimethylbutanoyl)-4-hydroxy-N-((S)-1-(4-(4-methylthiazol-5-yl)phenyl)ethyl)pyrrolidine-2-carboxamide was synthesized according to literature procedures[6]. ^{1}H NMR (500 MHz, CD_3OD) δ 8.89 (s, 1H), 7.55 (d, *J* = 8.2 Hz, 2H), 7.40 (d, *J* = 8.3 Hz, 2H), 5.00 (p, *J* = 6.8 Hz, 1H), 4.69 (s, 1H), 4.63 (t, *J* = 8.3 Hz, 1H), 4.52 (s, 1H), 4.12 (dd, *J* = 32.8, 15.1 Hz, 2H), 3.88 (d, *J* = 11.1 Hz, 1H), 3.82 – 3.73 (m, 3H), 3.19 (t, *J* = 5.0 Hz, 2H), 2.47 (s, 3H), 2.29 – 2.22 (m, 1H), 2.11 (ddd, *J* = 13.3, 9.1, 4.4 Hz, 1H), 1.50 (d, *J* = 7.0 Hz, 3H), 0.99 (s, 9H). ^{13}C NMR (126 MHz, CD_3OD) δ 173.58, 171.93, 171.47, 152.86, 148.84, 145.26, 131.13, 130.21, 127.86, 71.07, 70.63, 68.44, 60.75, 58.46, 58.15, 50.02, 49.85, 40.41, 38.94, 36.89, 26.89, 22.26, 15.81. LRMS (ESI): m/z $[M+H]^+$ Calcd for $C_{27}H_{40}N_5O_5S^+$: 546.27; found: 546.39.

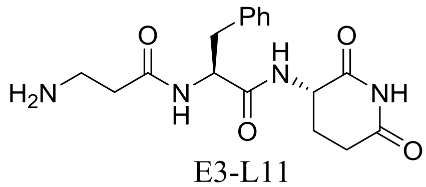

E3-L11

(S)-2-(3-aminopropanamido)-N-((S)-2,6-dioxopiperidin-3-yl)-3-phenylpropanamide was synthesized according to literature procedures[7]. ^{1}H NMR (500 MHz, CD_3OD) δ 7.32 – 7.21 (m, 5H), 4.69 (dd, J = 9.6, 5.1 Hz, 1H), 4.60 (dd, J = 11.8, 6.2 Hz, 1H), 3.24 (dd, J = 14.2, 4.8 Hz, 1H), 3.09 (t, J = 6.5 Hz, 2H), 2.93 (dd, J = 14.1, 9.7 Hz, 1H), 2.76 (ddd, J = 18.3, 12.2, 6.2 Hz, 1H), 2.70 – 2.59 (m, 2H), 2.51 (dt, J = 16.4, 6.3 Hz, 1H), 2.17 – 2.01 (m, 2H). ^{13}C NMR (126 MHz, CD_3OD) δ 174.79, 173.93, 173.23, 172.16, 138.41, 130.39, 130.33, 129.47, 127.84, 56.10, 51.23, 38.97, 36.98, 32.64, 32.01, 25.46. LRMS (ESI): m/z $[M+H]^+$ Calcd for $C_{17}H_{23}N_4O_4^+$: 347.16; found: 347.45.

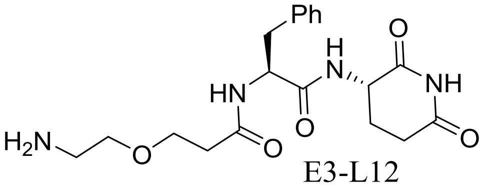

E3-L12

(S)-2-(3-(2-aminoethoxy)propanamido)-N-((S)-2,6-dioxopiperidin-3-yl)-3-phenylpropanamide was synthesized according to literature procedures[7]. ^{1}H NMR (500 MHz, CD_3OD) δ 7.34 – 7.19 (m, 5H), 4.70 (dd, *J* = 8.9, 4.5 Hz, 1H), 4.57 (dd, *J* = 12.0, 5.6 Hz, 1H), 3.71 – 3.64 (m, 2H), 3.59 (ddd, *J* = 15.8, 10.8, 5.7 Hz, 2H), 3.23 (dd, *J* = 14.0, 4.7 Hz, 1H), 3.07 (d, *J* = 4.4 Hz, 2H), 2.94 (dd, *J* = 14.0, 9.5 Hz, 1H), 2.80 – 2.64 (m, 2H), 2.53 (dt, *J* = 22.0, 7.2 Hz, 1H), 2.38 (dt, *J* = 15.0, 5.0 Hz, 1H), 2.17 – 1.98 (m, 2H). ^{13}C NMR (126 MHz, CD_3OD) δ 174.72, 173.97, 173.17, 138.38, 130.38, 129.46, 127.84, 67.85, 67.33, 55.89, 51.24, 49.85, 49.57, 40.46, 39.08, 36.97, 32.00, 25.44. LRMS (ESI): m/z $[M+H]^+$ Calcd for $C_{19}H_{27}N_4O_5^+$: 391.19; found: 391.39.

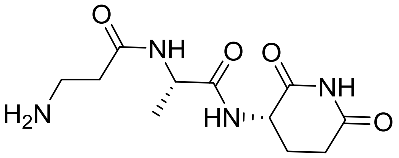

E3-L13

tert-butyl (3-(((S)-1-(((S)-2,6-dioxopiperidin-3-yl)amino)-1-oxopropan-2-yl)amino)-3-oxopropyl)carbamate was synthesized according to literature procedures[7]. The Boc-protected product was dissoloved in HCl -dioxane solution for 0.5h and the solvent was evaporated in vacuo to get E3-L13 without further purification.^{1}H NMR (500 MHz, DMSO-d6) δ 10.80 (s, 1H), 8.15 (d, *J* = 8.3 Hz, 1H), 8.06 (d, *J* = 7.7 Hz, 1H), 6.71 (t, *J* = 5.4 Hz, 1H), 4.59 – 4.50 (m, 1H), 4.29 (p, *J* = 7.2 Hz, 1H), 3.11 (dd, *J* = 13.3, 7.0 Hz, 2H), 2.77 – 2.68 (m, 1H), 2.47 (t, *J* = 3.4 Hz, 1H), 2.26 (t, *J* = 7.3 Hz, 2H), 1.92 (ddd, *J* = 10.6, 9.8, 5.9 Hz, 2H), 1.37 (s, 9H), 1.22 (d, *J* = 7.1 Hz, 3H).^{13}C NMR (126 MHz, DMSO-d6) δ 172.94, 172.45, 172.07, 170.08, 155.44, 77.57, 48.93, 47.88, 36.63, 35.54, 30.90, 28.24, 24.18, 18.26. LRMS (ESI): m/z $[M+H]^+$ Calcd for $C_{11}H_{19}N_4O_4^+$: 271.13; found: 271.39.

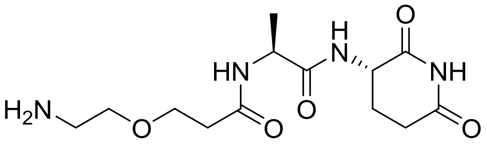

E3-L14

tert-butyl (2-(3-(((S)-1-(((S)-2,6-dioxopiperidin-3-yl)amino)-1-oxopropan-2-yl)amino)-3-oxopropoxy)ethyl)carbamate was synthesized according to literature procedures[7]. The Boc-protected product was dissoloved in HCl-dioxane solution for 0.5h and the solvent was evaporated in vacuo to get E3-L14 without further purification. ^{1}H NMR (500 MHz, DMSO-d6) δ 10.80 (s, 1H), 8.17 (d, *J* = 8.3 Hz, 1H), 8.05 (d, *J* = 7.6 Hz, 1H), 6.72 (t, *J* = 5.5 Hz, 1H), 4.55 (dd, *J* = 17.7, 8.3 Hz, 1H), 4.33 (dd, *J* = 14.5, 7.2 Hz, 1H), 3.57 (qd, *J* = 7.0, 2.9 Hz, 2H), 3.38 – 3.33 (m, 2H), 3.04 (q, *J* = 6.0 Hz, 2H), 2.77 – 2.69 (m, 1H), 2.47 (dd, *J* = 8.7, 5.0 Hz, 1H), 2.35 (tt, *J* = 14.7, 7.3 Hz, 2H), 1.92 (ddd, *J* = 11.8, 7.9, 4.1 Hz, 2H), 1.37 (s, 9H), 1.23 (d, *J* = 7.1 Hz, 3H).^{13}C NMR (126 MHz, DMSO-d6) δ 172.93, 172.44, 172.04, 169.76, 155.56, 77.59, 68.85, 66.42, 48.93, 47.88, 35.79, 30.90, 28.22, 24.17, 18.46. LRMS (ESI): m/z $[M+H]^+$ Calcd for $C_{13}H_{23}N_4O_5^+$: 315.16; found: 315.42.

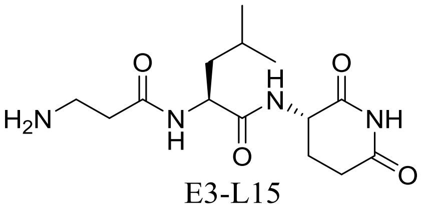

E3-L15

tert-butyl (3-(((S)-1-(((S)-2,6-dioxopiperidin-3-yl)amino)-4-methyl-1-oxopentan-2-yl)amino)-3-oxopropyl)carbamate was synthesized according to literature procedures[7].The Boc-protected product was dissoloved in HCl-dioxane solution for 0.5h and the solvent was evaporated in vacuo to get E3-L15 without further purification.^{1}H NMR (500 MHz, DMSO-d6) δ 10.78 (s, 1H), 8.18 (d, *J* = 8.3 Hz, 1H), 8.00 (d, *J* = 8.4 Hz, 1H), 6.68 (t, *J* = 5.4 Hz, 1H), 4.57 – 4.50 (m, 1H), 4.31 (td, *J* = 9.3, 5.5 Hz, 1H), 3.11 (dd, *J* = 13.7, 7.3 Hz, 2H), 2.77 – 2.69 (m, 1H), 2.47 (dd, *J* = 7.4, 4.0 Hz, 1H), 2.31 – 2.25 (m, 2H), 2.00 – 1.92 (m, 1H), 1.91 – 1.87 (m, 1H), 1.63 (dt, *J* = 13.1, 6.5 Hz, 1H), 1.46 (ddd, *J* = 19.0, 11.0, 5.4 Hz, 2H), 1.37 (s, 9H), 0.89 (d, *J* = 6.6 Hz, 3H), 0.84 (d, *J* = 6.5 Hz, 3H).^{13}C NMR (126 MHz, DMSO-d6) δ 172.97, 172.27, 172.01, 170.26, 155.41, 77.56, 50.74, 48.90, 40.97, 36.74, 35.55, 30.91, 28.23, 24.11, 23.10, 21.58.LRMS (ESI): m/z $[M+H]^+$ Calcd for $C_{14}H_{25}N_4O_4^+$: 313.18; found: 313.34.

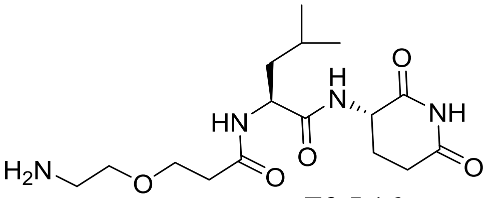

E3-L16

(S)-2-(3-(2-aminoethoxy)propanamido)-N-((S)-2,6-dioxopiperidin-3-yl)-4-methylpentanamide was synthesized according to literature procedures[7]. ^{1}H NMR (500 MHz, DMSO-d6) δ 10.79 (s, 1H), 8.35 (dd, *J* = 49.1, 7.6 Hz, 1H), 8.11 (d, *J* = 7.5 Hz, 1H), 7.88 (s, 3H, HCl salt), 4.54 (t, *J* = 15.3 Hz, 1H), 4.43 – 4.27 (m, 1H), 3.64 (s, 2H), 3.55 (s, 2H), 2.96 (s, 2H), 2.77 – 2.64 (m, 1H), 2.43 (d, *J* = 5.4 Hz, 2H), 2.01 – 1.83 (m, 2H), 1.70 – 1.56 (m, 1H), 1.47 (d, *J* = 19.1 Hz, 2H), 1.23 (s, 1H), 0.87 (dd, *J* = 20.4, 5.7 Hz, 6H). ^{13}C NMR (126 MHz, DMSO-d6) δ 173.00, 172.47, 172.00, 170.18, 66.47, 66.03, 50.93, 48.97, 41.12, 38.51, 35.50, 30.94, 24.14, 24.10, 23.10, 21.58. LRMS (ESI): m/z $[M+H]^+$ Calcd for $C_{16}H_{29}N_4O_5^+$: 357.21; found: 357.37.

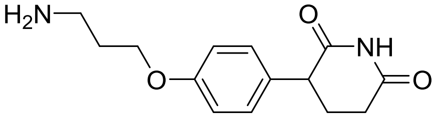

E3-L17

3-(4-(3-aminopropoxy)phenyl)piperidine-2,6-dione was synthesized according to literature procedures[8]. ^{1}H NMR (500 MHz, CD_3OD) δ 7.28 (t, *J* = 7.9 Hz, 1H), 6.93 – 6.82 (m, 3H), 4.12 (t, *J* = 5.6 Hz, 2H), 3.85 (dd, *J* = 10.3, 5.3 Hz, 1H), 3.15 (t, *J* = 7.2 Hz, 2H), 2.71 (ddd, *J* = 16.3, 10.5, 5.6 Hz, 1H), 2.66 – 2.59 (m, 1H), 2.29 – 2.18 (m, 2H), 2.14 (dd, *J* = 12.4, 6.2 Hz, 2H). ^{13}C NMR (126 MHz, CD_3OD) δ 176.42, 175.63, 160.11, 141.41, 130.78, 122.29, 116.10, 114.33, 66.19, 38.66, 32.13, 28.33, 27.70. LRMS (ESI): m/z $[M+H]^+$ Calcd for $C_{14}H_{19}N_2O_3^+$: 263.13; found: 263.34.

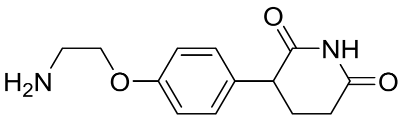

E3-L18

3-(4-(2-aminoethoxy)phenyl)piperidine-2,6-dione was synthesized according to literature procedures[8].
^{1}H NMR (500 MHz, CD_3OD) δ 7.31 (t, *J* = 8.1 Hz, 1H), 6.99 – 6.87 (m, 3H), 4.27 – 4.19 (m, 2H), 3.86 (dd, *J* = 10.5, 5.3 Hz, 1H), 3.41 – 3.33 (m, 2H), 2.71 (ddd, *J* = 16.4, 10.6, 5.6 Hz, 1H), 2.63 (dt, *J* = 17.5, 4.6 Hz, 1H), 2.30 – 2.17 (m, 2H). ^{13}C NMR (126 MHz, CD_3OD) δ 176.32, 175.60, 159.62, 141.59, 130.88, 122.88, 116.25, 114.42, 65.26, 40.29, 32.15, 27.67. LRMS (ESI): m/z $[M+H]^+$ Calcd for $C_{13}H_{17}N_2O_3^+$: 249.12; found: 249.22.

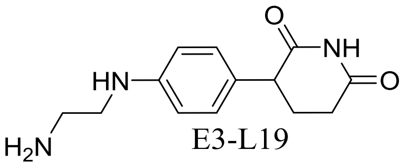

E3-L19

3-(4-((2-aminoethyl)amino)phenyl)piperidine-2,6-dione was synthesized according to literature procedures[8].
^{1}H NMR (500 MHz, CD_3OD) δ 7.04 (d, *J* = 8.3 Hz, 2H), 6.68 (d, *J* = 8.3 Hz, 2H), 3.76 – 3.71 (m, 1H), 3.42 (t, *J* = 5.9 Hz, 2H), 3.13 (t, *J* = 5.8 Hz, 2H), 2.66 (dd, *J* = 16.7, 9.2 Hz, 1H), 2.59 (dt, *J* = 17.5, 4.7 Hz, 1H), 2.17 (dd, *J* = 11.2, 6.2 Hz, 2H). ^{13}C NMR (126 MHz, CD_3OD) δ 177.18, 175.84, 148.52, 130.25, 128.65, 114.23, 48.35, 42.16, 39.96, 31.97, 27.83. LRMS (ESI): m/z $[M+H]^+$ Calcd for $C_{13}H_{18}N_3O_2^+$: 248.13; found: 248.21.

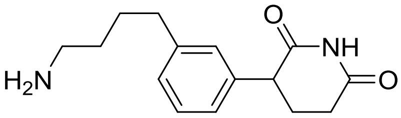

E3-L20

3-(3-(4-aminobutyl)phenyl)piperidine-2,6-dione was synthesized according to literature procedures[8].
^{1}H NMR (500 MHz, DMSO-d6) δ 10.84 (s, 1H), 7.81 (s, 3H, HCl salt), 7.25 (t, *J* = 7.4 Hz, 1H), 7.11 (d, *J* = 7.2 Hz, 1H), 7.04 (d, *J* = 13.8 Hz, 2H), 3.87 – 3.77 (m, 1H), 2.78 (t, *J* = 6.3 Hz, 2H), 2.69 – 2.63 (m, 1H), 2.57 (d, *J* = 6.9 Hz, 2H), 2.46 (s, 1H), 2.22 – 2.13 (m, 1H), 2.03 (dd, *J* = 10.6, 6.4 Hz, 1H), 1.63 – 1.53 (m, 4H). ^{13}C NMR (126 MHz, DMSO-d6) δ 174.27, 173.43, 141.71, 139.21, 128.56, 128.26, 126.85, 126.08, 47.32, 38.64, 34.53, 31.30, 27.65, 26.71, 26.07. LRMS (ESI): m/z $[M+H]^+$ Calcd for $C_{15}H_{21}N_2O_2^+$: 261.15; found: 261.25.

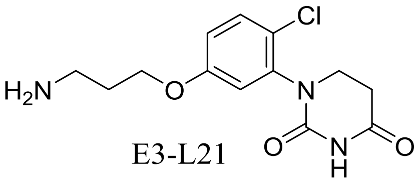

E3-L21

1-(5-(3-aminopropoxy)-2-chlorophenyl)dihydropyrimidine-2,4(1H,3H)-dione was synthesized according to literature procedures[9]. ^{1}H NMR (500 MHz, CD_3OD) δ 7.45 (d, *J* = 8.9 Hz, 1H), 7.08 (d, *J* = 2.7 Hz, 1H), 7.00 (dd, *J* = 8.9, 2.8 Hz, 1H), 4.13 (t, *J* = 5.6 Hz, 2H), 3.76 (t, *J* = 6.7 Hz, 2H), 3.15 (t, *J* = 7.2 Hz, 2H), 2.85 (qd, *J* = 16.7, 10.0 Hz, 2H), 2.19 – 2.11 (m, 2H). ^{13}C NMR (126 MHz, CD_3OD) δ 172.73, 159.49, 154.27, 140.49,

131.78, 125.26, 117.19, 117.02, 66.81, 46.00, 38.49, 32.03, 28.18. LRMS (ESI): m/z $[M+H]^+$ Calcd for $C_{13}H_{17}ClN_3O_3^+$: 298.09; found: 298.25.

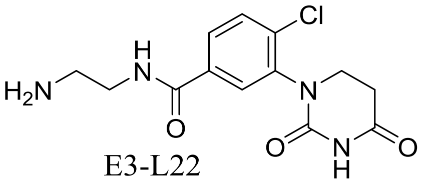

E3-L22

N-(2-aminoethyl)-4-chloro-3-(2,4-dioxotetrahydropyrimidin-1(2H)-yl)benzamide was synthesized according to literature procedures[9]. ^{1}H NMR (500 MHz, DMSO-d6) δ 10.55 (s, 1H), 8.76 (s, 1H), 7.96 (s, 1H), 7.92 – 7.77 (m, 4H, HCl salt), 7.72 (d, *J* = 8.2 Hz, 1H), 3.76 – 3.72 (m, 1H), 3.64 (d, *J* = 6.2 Hz, 1H), 3.51 (d, *J* = 5.7 Hz, 2H), 2.99 (d, *J* = 5.3 Hz, 2H), 2.76 (t, *J* = 6.5 Hz, 2H). ^{13}C NMR (126 MHz, DMSO-d6) δ 170.55, 165.30, 151.85, 138.98, 135.01, 134.06, 129.88, 129.20, 127.97, 44.26, 38.59, 37.18, 31.04. LRMS (ESI): m/z $[M+H]^+$ Calcd for $C_{13}H_{16}ClN_4O_3^+$: 311.08; found: 311.21.

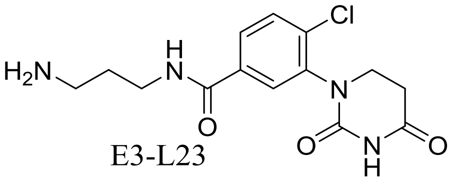

E3-L23

N-(3-aminopropyl)-4-chloro-3-(2,4-dioxotetrahydropyrimidin-1(2H)-yl)benzamide was synthesized according to literature procedures[9]. ^{1}H NMR (500 MHz, DMSO-d6) δ 10.54 (s, 1H), 8.78 (t, *J* = 5.6 Hz, 1H), 7.96 (d, *J* = 2.4 Hz, 1H), 7.85 (dd, *J* = 8.4, 2.3 Hz, 1H), 7.81 (s, 3H, HCl salt), 7.70 (d, *J* = 8.5 Hz, 1H), 3.75 (dt, *J* = 13.6, 7.0 Hz, 1H), 3.63 (dt, J = 12.3, 6.3 Hz, 1H), 3.34 (d, J = 7.3 Hz, 2H), 2.84 (d, J = 9.3 Hz, 2H), 2.75 (dt, J = 6.9, 4.4 Hz, 2H), 1.90 – 1.73 (m, 2H). ^{13}C NMR (126 MHz, DMSO-d6) δ 170.61, 164.89, 151.88, 139.02, 134.88, 134.23, 129.93, 129.07, 127.82, 44.26, 36.94, 36.50, 31.06, 27.40. LRMS (ESI): m/z $[M+H]^+$ Calcd for $C_{14}H_{18}ClN_4O_3^+$: 325.10; found: 325.26.

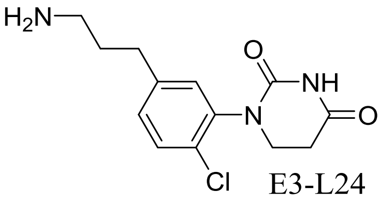

E3-L24

1-(5-(3-aminopropyl)-2-chlorophenyl)dihydropyrimidine-2,4(1H,3H)-dione was synthesized according to literature procedures[9]. ^{1}H NMR (500 MHz, CD_3OD) δ 7.48 (d, *J* = 8.2 Hz, 1H), 7.35 (d, *J* = 1.4 Hz, 1H), 7.27 (d, *J* = 8.2 Hz, 1H), 3.76 (t, *J* = 6.6 Hz, 2H), 2.97 – 2.92 (m, 2H), 2.90 – 2.80 (m, 2H), 2.74 (t, *J* = 7.6 Hz, 2H), 2.01 – 1.94 (m, 2H). ^{13}C NMR (126 MHz, CD_3OD) δ 172.77, 154.29, 142.59, 139.81, 131.36, 130.94, 130.79, 46.04, 40.06, 32.57, 32.03, 29.86. LRMS (ESI): m/z $[M+H]^+$ Calcd for $C_{13}H_{17}ClN_3O_2^+$: 282.09; found: 282.23.

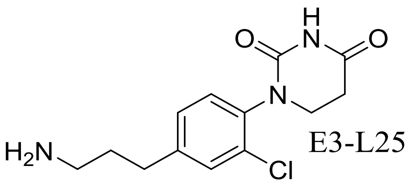

1-(4-(3-aminopropyl)-2-chlorophenyl)dihydropyrimidine-2,4(1H,3H)-dione was synthesized according to literature procedures[9]. ^{1}H NMR (500 MHz, CD_3OD) δ 7.45 (s, 1H), 7.39 (d, *J* = 8.0 Hz, 1H), 7.29 – 7.26 (m, 1H), 3.75 (dd, *J* = 7.7, 5.9 Hz, 2H), 2.97 – 2.93 (m, 2H), 2.88 – 2.79 (m, 2H), 2.74 (t, *J* = 7.6 Hz, 2H), 2.00 – 1.94 (m, 2H). ^{13}C NMR (126 MHz, CD_3OD) δ 172.81, 154.33, 144.22, 138.04, 133.63, 131.11, 131.01, 129.35, 46.07, 40.08, 32.74, 32.02, 29.95. LRMS (ESI): m/z $[M+H]^+$ Calcd for $C_{13}H_{17}ClN_3O_2^+$: 282.09; found: 282.27.

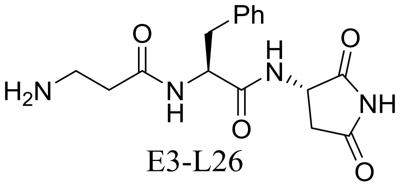

(S)-2-(3-aminopropanamido)-N-((S)-2,5-dioxopyrrolidin-3-yl)-3-phenylpropanamide was synthesized according to literature procedures[7]. ^{1}H NMR (500 MHz, CD_3OD) δ 7.31 – 7.21 (m, 5H), 4.67 – 4.61 (m, 1H), 4.40 (dd, *J* = 9.0, 5.7 Hz, 1H), 3.12 (dt, *J* = 12.1, 6.1 Hz, 3H), 2.94 – 2.86 (m, 2H), 2.58 (ddd, *J* = 36.9, 24.0, 13.4 Hz, 3H). ^{13}C NMR (126 MHz, CD_3OD) δ 178.87, 177.95, 173.77, 172.03, 138.16, 130.34, 129.54, 127.95, 55.61, 51.64, 38.84, 36.92, 32.53. LRMS (ESI): m/z $[M+H]^+$ Calcd for $C_{16}H_{21}N_4O_4^+$: 333.15; found: 333.34.

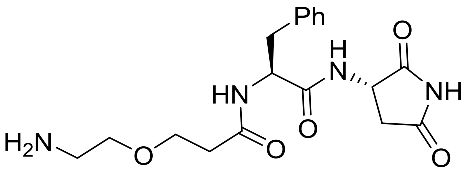

(S)-2-(3-(2-aminoethoxy)propanamido)-N-((S)-2,5-dioxopyrrolidin-3-yl)-3-phenylpropanamide was synthesized according to literature procedures[7]. ^{1}H NMR (500 MHz, CD_3OD) δ 7.31 – 7.23 (m, 5H), 4.64 (dd, *J* = 8.3, 6.5 Hz, 1H), 4.42 (dd, *J* = 9.1, 5.7 Hz, 1H), 3.68 (t, *J* = 4.8 Hz, 2H), 3.59 (ddd, *J* = 15.9, 10.5, 5.0 Hz, 2H), 3.11 (d, *J* = 7.4 Hz, 1H), 3.07 (s, 2H), 2.91 (ddd, *J* = 22.5, 15.9, 8.9 Hz, 2H), 2.56 – 2.50 (m, 2H), 2.40 (dt, *J* = 15.1, 5.0 Hz, 1H).^{13}C NMR (126 MHz, CD_3OD) δ 178.79, 177.91, 173.95, 173.90, 138.13, 130.39, 129.52, 127.94, 67.74, 67.32, 55.62, 51.54, 40.42, 38.90, 36.93. LRMS (ESI): m/z $[M+H]^+$ Calcd for $C_{18}H_{25}N_4O_5^+$: 377.17; found: 377.34.

1.4.2. POI Ligands used in this work

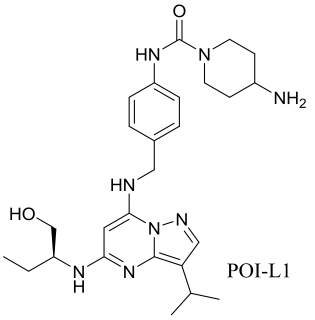

POI-L1

(S)-4-amino-N-(4-(((5-((1-hydroxybutan-2-yl)amino)-3-isopropylpyrazolo[1,5-a]pyrimidin-7-yl)amino)methyl)phenyl)piperidine-1-carboxamide was synthesized according to literature procedures[10]. ^{1}H NMR (500 MHz, CD_3OD) δ 7.87 (s, 1H), 7.38 (d, *J* = 8.6 Hz, 2H), 7.33 (d, *J* = 8.6 Hz, 2H), 5.40 (s, 1H), 4.63 (s, 2H), 4.26 (d, *J* = 13.9 Hz, 2H), 3.77 – 3.54 (m, 3H), 3.39 – 3.33 (m, 1H), 3.06 (dt, *J* = 13.8, 6.9 Hz, 1H), 2.98 (t, *J* = 12.1 Hz, 2H), 2.05 (dd, *J* = 12.1, 2.1 Hz, 2H), 1.68 (dt, *J* = 13.3, 7.4 Hz, 1H), 1.57 (qd, *J* = 12.4, 4.2 Hz, 3H), 1.30 (dd, *J* = 6.9, 2.5 Hz, 6H), 0.97 (t, *J* = 7.2 Hz, 3H). ^{13}C NMR (126 MHz, CD_3OD) δ 157.55, 143.54, 140.65, 135.10, 132.32, 128.68, 122.41, 46.27, 43.61, 31.05, 24.96, 23.98, 23.43, 23.39, 10.77. LRMS (ESI): m/z $[M+H]^+$ Calcd for $C_{26}H_{39}N_8O_2^+$: 495.31; found: 495.43.

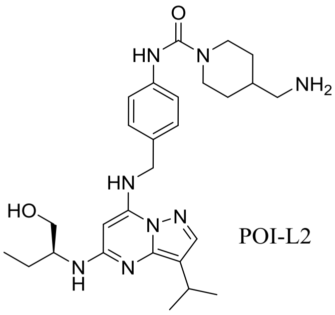

POI-L2

(S)-4-(aminomethyl)-N-(4-(((5-((1-hydroxybutan-2-yl)amino)-3-isopropylpyrazolo[1,5-a]pyrimidin-7-yl)amino)methyl)phenyl)piperidine-1-carboxamide was synthesized according to literature procedures[10].

^{1}H NMR (500 MHz, CD_3OD) δ 7.89 (s, 1H), 7.39 (d, *J* = 8.6 Hz, 2H), 7.34 (d, *J* = 8.6 Hz, 2H), 5.42 (s, 1H), 4.64 (s, 2H), 4.25 (d, *J* = 13.6 Hz, 2H), 3.80 – 3.57 (m, 3H), 3.11 – 3.03 (m, 1H), 2.96 – 2.87 (m, 4H), 1.96 – 1.88 (m, 1H), 1.85 (d, *J* = 13.1 Hz, 2H), 1.74 – 1.67 (m, 1H), 1.60 (dt, *J* = 12.8, 5.5 Hz, 1H), 1.32 (dd, *J* = 6.9, 2.8 Hz, 6H), 1.30 – 1.24 (m, 2H), 0.99 (t, *J* = 7.2 Hz, 3H). ^{13}C NMR (126 MHz, CD_3OD) δ 157.66, 143.57, 140.80, 132.14, 128.66, 122.41, 46.29, 45.51, 44.88, 35.80, 30.38, 24.95, 23.98, 23.43, 23.38, 10.77. LRMS (ESI): m/z $[M+H]^+$ Calcd for $C_{27}H_{41}N_8O_2^+$: 509.33; found: 509.48.

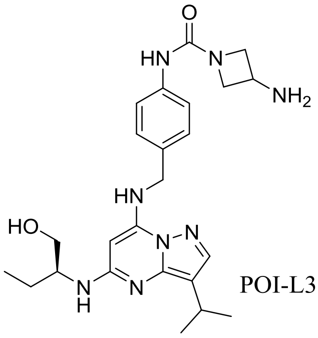

POI-L3

(S)-3-amino-N-(4-(((5-((1-hydroxybutan-2-yl)amino)-3-isopropylpyrazolo[1,5-a]pyrimidin-7-yl)amino)methyl)phenyl)azetidine-1-carboxamide was synthesized according to literature procedures[10].

^{1}H NMR (500 MHz, CD_3OD) δ 7.87 (s, 1H), 7.46 (d, *J* = 8.6 Hz, 2H), 7.33 (d, *J* = 8.6 Hz, 2H), 5.39 (s, 1H), 4.67 – 4.58 (m, 2H), 4.40 (dd, *J* = 9.6, 7.5 Hz, 2H), 4.15 – 4.10 (m, 1H), 4.05 (dd, *J* = 9.6, 4.6 Hz, 2H), 3.79 – 3.62 (m, 2H), 3.61 – 3.57 (m, 1H), 3.05 (dt, *J* = 13.9, 6.9 Hz, 1H), 1.71 – 1.65 (m, 1H), 1.58 (dt, *J* = 14.3, 7.4 Hz, 1H), 1.31 (dd, *J* = 6.9, 3.2 Hz, 6H), 0.97 (t, *J* = 7.2 Hz, 3H). ^{13}C NMR (126 MHz, CD_3OD) δ 158.89, 143.54, 140.07, 135.12, 132.32, 128.75, 121.50, 54.89, 46.25, 41.43, 24.94, 23.96, 23.43, 23.38, 10.76. LRMS (ESI): m/z $[M+H]^+$ Calcd for $C_{24}H_{35}N_8O_2^+$: 467.28; found: 467.42.

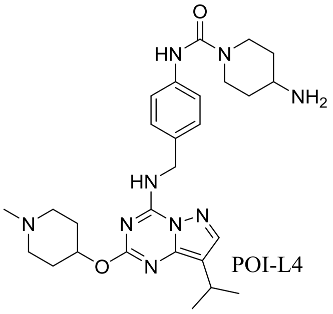

POI-L4

4-amino-N-(4-(((8-isopropyl-2-((1-methylpiperidin-4-yl)oxy)pyrazolo[1,5-a][1,3,5]triazin-4-yl)amino)methyl)phenyl)piperidine-1-carboxamide was synthesized according to literature procedures[10].

^{1}H NMR (500 MHz, CD_3OD) δ 7.85 (s, 1H), 7.32 (q, *J* = 8.7 Hz, 4H), 4.72 (s, 2H), 4.26 (d, *J* = 13.8 Hz, 2H), 3.56 (dd, *J* = 34.4, 12.5 Hz, 1H), 3.44 – 3.34 (m, 3H), 3.17 (dd, *J* = 21.5, 9.8 Hz, 1H), 3.08 – 3.03 (m, 1H), 2.97 (t, *J* = 12.5 Hz, 2H), 2.90 (d, *J* = 5.8 Hz, 3H), 2.31 (dd, *J* = 39.0, 14.0 Hz, 2H), 2.21 – 1.95 (m, 4H), 1.89 (dd, *J* = 22.5, 11.2 Hz, 1H), 1.57 (qd, *J* = 12.3, 4.1 Hz, 2H), 1.29 (t, *J* = 6.4 Hz, 6H).

^{13}C NMR (126 MHz, CD_3OD) δ 160.54, 157.64, 151.93, 145.45, 140.21, 134.03, 128.80, 128.66, 122.41, 115.47, 71.03, 67.37, 53.68, 50.98, 44.68, 44.05, 43.61, 40.42, 31.08, 29.63, 28.52, 24.65, 23.59. LRMS (ESI): m/z $[M+H]^+$ Calcd for $C_{27}H_{40}N_9O_2^+$: 522.32; found: 522.63.

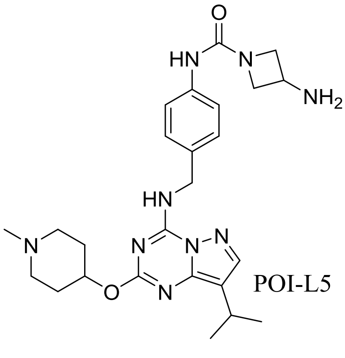

POI-L5

3-amino-N-(4-(((8-isopropyl-2-((1-methylpiperidin-4-yl)oxy)pyrazolo[1,5-a][1,3,5]triazin-4-yl)amino)methyl) phenyl)azetidine-1-carboxamide was synthesized according to literature procedures[10].

^{1}H NMR (500 MHz, CD_3OD) δ 7.86 (s, 1H), 7.41 (d, *J* = 8.5 Hz, 2H), 7.31 (d, *J* = 8.4 Hz, 2H), 4.72 (d, *J* = 4.0 Hz, 2H), 4.39 (dd, *J* = 9.5, 7.5 Hz, 2H), 4.12 (dt, *J* = 14.8, 5.9 Hz, 1H), 4.06 (dd, *J* = 9.5, 4.5 Hz, 2H), 3.59 (d, *J* = 12.9 Hz, 1H), 3.45 – 3.35 (m, 2H), 3.19 (dd, *J* = 19.3, 6.1 Hz, 1H), 3.06 (dd, *J* = 13.7, 6.8 Hz, 1H), 2.91 (s, 3H), 2.33 (dd, *J* = 34.9, 14.7 Hz, 2H), 2.21 – 2.01 (m, 2H), 1.98 – 1.84 (m, 1H), 1.30 (t, *J* = 6.5 Hz, 6H). ^{13}C NMR (126 MHz, CD_3OD) δ 160.56, 159.01, 151.92, 146.94, 145.42, 139.63, 134.03, 128.86, 128.75, 121.48, 115.47, 70.96, 67.33, 54.88, 53.67, 50.96, 44.65, 44.04, 41.46, 29.62, 28.50, 24.64, 23.58. LRMS (ESI): m/z $[M+H]^+$ Calcd for $C_{25}H_{36}N_9O_2^+$: 494.29; found: 494.43.

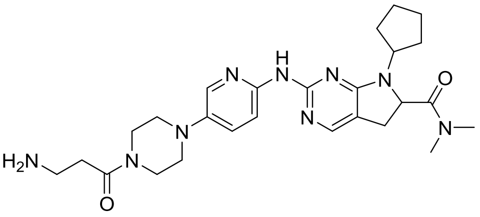

POI-L6

2-((5-(4-(3-aminopropanoyl)piperazin-1-yl)pyridin-2-yl)amino)-7-cyclopentyl-N,N-dimethyl-6,7-dihydro-5H-pyrrolo[2,3-d]pyrimidine-6-carboxamide was synthesized according to literature procedures[11]. ^{1}H NMR (500 MHz, DMSO-d6) δ 10.54 (s, 1H), 8.89 (s, 1H), 7.97 (s, 1H), 7.83 (s, 2H), 7.73 (s, 3H, HCl salt), 6.73 (s, 1H), 4.80 – 4.75 (m, 1H), 3.68 – 3.65 (m, 2H), 3.62 – 3.59 (m, 2H), 3.22 – 3.18 (m, 2H), 3.16 – 3.13 (m, 2H), 3.09 – 3.00 (m, 9H), 2.74 (t, *J* = 6.4 Hz, 2H), 2.40 – 2.33 (m, 2H), 2.03 – 1.95 (m, 4H), 1.68 – 1.62 (m, 2H).^{13}C NMR (126 MHz, DMSO-d6) δ 168.29, 162.50, 158.04, 157.79, 152.91, 151.45, 145.08, 141.85, 118.30, 115.92, 114.20, 113.08, 100.87, 56.97, 54.92, 48.58, 48.28, 44.28, 40.72, 35.20, 29.93, 29.78, 24.20. LRMS (ESI): m/z $[M+H]^+$ Calcd for $C_{26}H_{38}N_9O_2^+$: 508.31; found:508.54.

1.4.3 Standard Products obtained *via* organic synthesis for UPLC-MS analysis

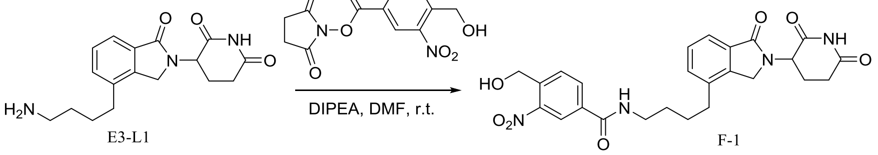

E3-L1 (50.0 mg, 0.14mmol, 1.0 equiv.) was dissolved in 2mL DMF and 2,5-dioxopyrrolidin-1-yl 4-(hydroxymethyl)-3-nitrobenzoate (46.0 mg, 0.16mmol, 1.1 equiv.) was added. DIPEA (30 µl, 0.16mmol,

1.1equiv.) was added and the reation was stired under room temperature for 1h. The solvent was evaporated and the residue was purified by column chromatography to afford N-(4-(2-(2,6-dioxopiperidin-3-yl)-1-oxoisoindolin-4-yl)butyl)-4-(hydroxymethyl)-3-nitrobenzamide (F-1) (56.1mg, 81.0% yield). ^{1}H NMR (500 MHz, DMSO-d6) δ 10.98 (s, 1H), 8.77 (t, *J* = 5.6 Hz, 1H), 8.49 (d, *J* = 1.7 Hz, 1H), 8.20 (dd, *J* = 8.1, 1.7 Hz, 1H), 7.92 (d, *J* = 8.2 Hz, 1H), 7.56 (dd, *J* = 6.8, 1.4 Hz, 1H), 7.47 (d, *J* = 2.4 Hz, 2H), 5.12 (d, *J* = 8.2 Hz, 1H), 4.87 (s, 2H), 4.47 (d, *J* = 17.1 Hz, 1H), 4.31 (d, *J* = 17.1 Hz, 1H), 3.34 (dd, *J* = 5.6, 3.5 Hz, 2H), 2.95 – 2.89 (m, 1H), 2.69 (t, *J* = 7.5 Hz, 2H), 2.59 (d, *J* = 17.2 Hz, 1H), 2.39 (dd, *J* = 13.1, 4.3 Hz, 1H), 2.01 – 1.98 (m, 1H), 1.67 (dd, *J* = 10.4, 5.2 Hz, 2H), 1.62 – 1.58 (m, 2H).^{13}C NMR (126 MHz, DMSO-d6) δ 172.89, 171.05, 168.35, 163.93, 146.57, 141.22, 140.54, 137.35, 133.99, 132.07, 131.57, 128.46, 128.30, 123.04, 120.67, 59.89, 51.52, 46.23, 31.19, 30.85, 28.80, 26.71, 22.51. LRMS (ESI): m/z $[M+H]^+$ Calcd for $C_{25}H_{27}N_4O_7^+$: 495.18; found: 495.47.

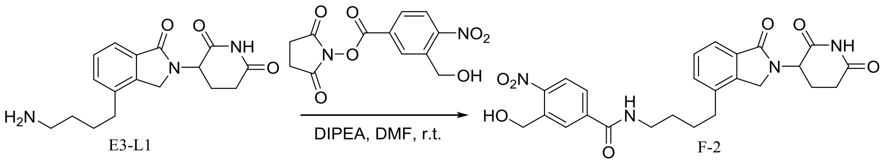

N-(4-(2-(2,6-dioxopiperidin-3-yl)-1-oxoisoindolin-4-yl)butyl)-3-(hydroxymethyl)-4-nitrobenzamide (S-F-2) was synthesized following the similar procedures as F-1 (50.2mg, 72.6% yield). ^{1}H NMR (500 MHz, DMSO-d6) δ 11.00 (s, 1H), 8.81 (s, 1H), 8.25 (s, 1H), 8.11 (d, *J* = 8.4 Hz, 1H), 7.89 (d, *J* = 8.2 Hz, 1H), 7.57 (d, *J* = 6.4 Hz, 1H), 7.49 – 7.43 (m, 2H), 5.13 (dd, *J* = 13.1, 4.4 Hz, 1H), 4.83 (s, 2H), 4.47 (d, *J* = 17.0 Hz, 1H), 4.31 (d, *J* = 17.1 Hz, 1H), 3.33 (s, 2H), 2.96 – 2.88 (m, 1H), 2.68 (d, *J* = 7.0 Hz, 2H), 2.59 (d, *J* = 16.9 Hz, 1H), 2.39 (dd, *J* = 12.9, 3.1 Hz, 1H), 2.02 – 1.97 (m, 1H), 1.68 – 1.57 (m, 4H). ^{13}C NMR (126 MHz, DMSO-d6) δ 172.90, 171.06, 168.37, 164.79, 148.13, 140.54, 138.90, 138.32, 137.37, 131.58, 128.31, 127.54, 126.46, 124.48, 120.68, 59.81, 51.53, 46.23, 31.19, 30.85, 28.81, 26.72, 22.52. LRMS (ESI): m/z $[M+H]^+$ Calcd for $C_{25}H_{27}N_4O_7^+$: 495.18; found: 495.28.

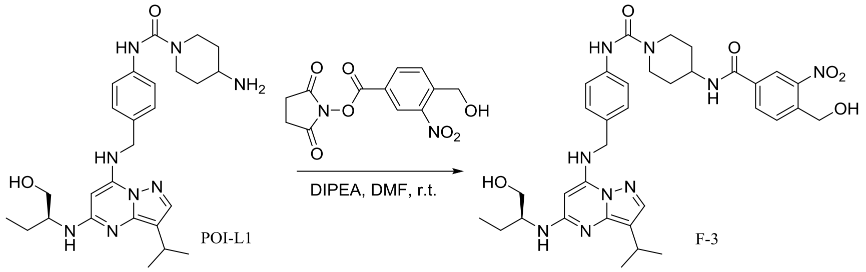

(S)-N-(4-(((5-((1-hydroxybutan-2-yl)amino)-3-isopropylpyrazolo[1,5-a]pyrimidin-7-yl)amino)methyl)phenyl)-4-(4-(hydroxymethyl)-3-nitrobenzamido)piperidine-1-carboxamide (S-F-3) was synthesized following the similar procedures as F-1 (72.0mg, 76.4% yield). ^{1}H NMR (500 MHz, CD_3OD) δ 8.53 (d, *J* = 1.8 Hz, 1H), 8.17 (dd, *J* = 8.1, 1.8 Hz, 1H), 7.98 (d, *J* = 8.2 Hz, 1H), 7.87 (s, 1H), 7.39 (d, *J* = 8.6 Hz, 2H), 7.32 (d, *J* = 8.6 Hz, 2H), 5.40 (s, 1H), 4.99 (s, 2H), 4.63 (s, 2H), 4.22 (d, *J* = 13.6 Hz, 2H), 4.14 (ddd, *J* = 11.3, 7.1, 4.3 Hz, 1H), 3.75 – 3.58 (m, 3H), 3.05 (t, *J* = 11.8 Hz, 3H), 2.03 (dd, *J* = 12.9, 3.0 Hz, 2H), 1.72 – 1.58 (m, 4H), 1.31 (dd, *J* = 6.9, 3.3 Hz, 6H), 0.98 (t, *J* = 7.4 Hz, 3H). ^{13}C NMR (126 MHz, CD_3OD) δ 167.08, 157.72, 148.43, 143.63, 142.60, 140.87, 135.59, 134.89, 133.12, 132.13, 129.77, 128.64, 124.61, 122.43, 61.80, 46.31, 44.53, 40.42, 32.60, 24.98, 24.10, 23.43, 23.35, 10.80. LRMS (ESI): m/z $[M+H]^+$ Calcd for $C_{34}H_{44}N_9O_6^+$: 674.33; found: 674.53.

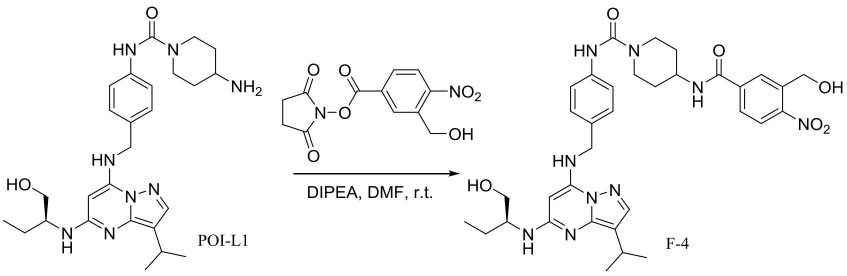

(S)-N-(4-(((5-((1-hydroxybutan-2-yl)amino)-3-isopropylpyrazolo[1,5-a]pyrimidin-7-yl)amino)methyl)phenyl)-4-(3-(hydroxymethyl)-4-nitrobenzamido)piperidine-1-carboxamide (S-F-4) was synthesized following the similar procedures as F-1 (68.8mg, 73.0% yield). ^{1}H NMR (500 MHz, CD_3OD) δ 8.30 – 8.26 (m, 1H), 8.11 (d, *J* = 8.4 Hz, 1H), 7.90 – 7.85 (m, 2H), 7.39 (d, *J* = 8.6 Hz, 2H), 7.32 (d, *J* = 8.6 Hz, 2H), 5.40 (s, 1H), 4.96 (s, 2H), 4.63 (s, 2H), 4.22 (d, *J* = 13.6 Hz, 2H), 4.17 – 4.12 (m, 1H), 3.76 – 3.58 (m, 3H), 3.06 (t, *J* = 11.7 Hz, 3H), 2.03 (dd, *J* = 12.4, 2.5 Hz, 2H), 1.71 – 1.57 (m, 4H), 1.31 (dd, *J* = 6.9, 3.4 Hz, 6H), 0.98 (t, *J* = 7.4 Hz, 3H). ^{13}C NMR (126 MHz, CD_3OD) δ 168.02, 158.79, 157.72, 154.01, 150.15, 143.64, 140.86, 140.42, 139.44, 134.90, 132.14, 128.89, 128.63, 127.84, 125.75, 122.43, 61.68, 46.31, 44.50, 40.42, 32.58, 24.97, 24.11, 23.42, 23.35, 10.80. LRMS (ESI): m/z $[M+H]^+$ Calcd for $C_{34}H_{44}N_9O_6^+$: 674.33; found: 674.50.

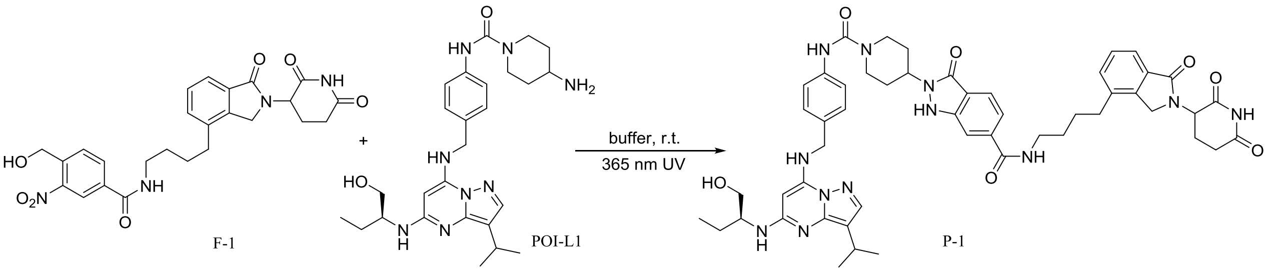

200μL of F-1 stock (100mM in DMSO) was mixxed with 800μL of POI-L1 stock (100mM in DMSO) in 9mL of reaction buffer (20mM PBS/ DMSO=1:1, pH=10.5). The reaction was exposed to 365 nm UV for 30 min at room temperature. Water was evaporated and the residue was purified by HPLC to afford P-1 as white powder. ^{1}H NMR (500 MHz, CD_3OD) δ 7.88 (s, 1H), 7.83 (d, *J* = 8.3 Hz, 1H), 7.71 (s, 1H), 7.64 (d, *J* = 7.5 Hz, 1H), 7.55 (t, *J* = 7.0 Hz, 1H), 7.50 (d, *J* = 7.3 Hz, 1H), 7.45 (d, *J* = 7.5 Hz, 1H), 7.41 (d, *J* = 8.5 Hz, 2H), 7.34 (d, *J* = 8.3 Hz, 2H), 5.41 (s, 1H), 5.13 (dd, *J* = 13.4, 5.1 Hz, 1H), 4.71 – 4.62 (m, 3H), 4.50 (d, *J* = 16.8 Hz, 1H), 4.44 (d, *J* = 16.9 Hz, 1H), 4.36 (d, *J* = 13.8 Hz, 2H), 3.78 – 3.69 (m, 1H), 3.69 – 3.62 (m, 1H), 3.60 (dd, *J* = 11.0, 6.1 Hz, 1H), 3.47 (t, *J* = 6.5 Hz, 2H), 3.09 (t, *J* = 12.6 Hz, 2H), 2.87 (ddd, *J* = 13.4, 12.3, 5.4 Hz, 1H), 2.79 (t, *J* = 7.1 Hz, 2H), 2.73 (d, *J* = 17.8 Hz, 1H), 2.43 (tt, *J* = 13.4, 6.8 Hz, 1H), 2.15 – 2.08 (m, 1H), 2.06 – 1.97 (m, 4H), 1.82 – 1.65 (m, 6H), 1.60 (ddd, *J* = 12.4, 6.5, 2.5 Hz, 1H), 1.31 (dd, *J* = 6.8, 4.2 Hz, 6H), 0.99 (t, *J* = 7.2 Hz, 3H). ^{13}C NMR (126 MHz, CD_3OD) δ 174.56, 172.22, 171.83, 169.48, 162.19, 157.63, 147.18, 143.61, 141.99, 140.79, 139.58, 138.89, 134.94, 133.36, 132.60, 132.23, 129.68, 128.67, 124.39, 122.44, 122.16, 121.10, 120.25, 112.75, 53.68, 53.60, 48.18, 46.31, 44.74, 40.56, 32.41, 32.35, 31.05, 30.09, 28.27, 24.98, 24.06, 23.43, 23.37, 10.81. HRMS (ESI-Q-TOF): m/z $[M+H]^+$ Calcd for $C_{51}H_{61}N_{12}O_7^+$: 953.4786; found: 953.4782.

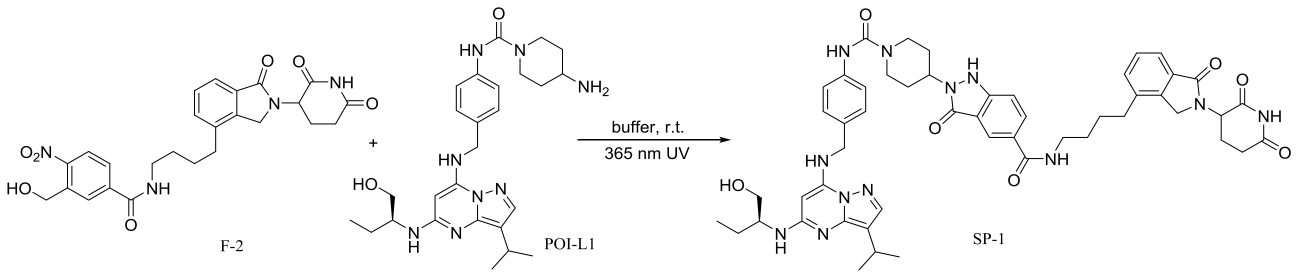

SP-1 was synthesized following the similar procedures as P-1 by using POI-L1 and F-2 to afford white powder. ^{1}H NMR (500 MHz, CD_3OD) δ 8.23 (d, *J* = 1.0 Hz, 1H), 8.01 (dd, *J* = 8.7, 1.6 Hz, 1H), 7.87 (s, 1H), 7.64 (dd, *J* = 7.3, 0.9 Hz, 1H), 7.50 (d, *J* = 6.7 Hz, 1H), 7.46 (t, *J* = 7.4 Hz, 1H), 7.41 (d, *J* = 8.6 Hz, 2H), 7.36 – 7.31 (m, 3H), 5.40 (s, 1H), 5.13 (dd, *J* = 13.4, 5.2 Hz, 1H), 4.70 – 4.62 (m, 3H), 4.50 (d, *J* = 16.9 Hz, 1H), 4.45 (d, *J* = 16.9 Hz, 1H), 4.36 (d, *J* = 13.4 Hz, 2H), 3.73 (dd, *J* = 11.7, 5.4 Hz, 1H), 3.68 – 3.64 (m, 1H), 3.60 (d, *J* = 4.7 Hz, 1H), 3.49 – 3.45 (m, 2H), 3.12 – 3.06 (m, 2H), 2.91 – 2.86 (m, 1H), 2.79 (t, *J* = 7.5 Hz, 2H), 2.75 – 2.71 (m, 1H), 2.43 (dd, *J* = 13.1, 4.6 Hz, 1H), 2.14 – 2.10 (m, 1H), 2.05 – 2.02 (m, 1H), 1.99 (dd, *J* = 8.1, 3.5 Hz, 3H), 1.80 – 1.67 (m, 6H), 1.59 (d, *J* = 7.7 Hz, 1H), 1.31 (dd, *J* = 6.9, 3.5 Hz, 6H), 0.99 (t, *J* = 7.4 Hz, 3H). ^{13}C NMR (126 MHz, CD_3OD) δ 174.65, 172.20, 171.86, 169.39, 162.40, 157.65, 148.53, 143.55, 142.00, 140.79, 138.98, 133.43, 132.60, 132.32, 129.69, 129.21, 128.66, 123.75, 122.45, 122.15, 117.53, 112.95, 54.80, 53.59, 48.19, 46.31, 44.75, 40.33, 32.50, 32.38, 31.08, 30.23, 28.27, 25.00, 24.13, 24.07, 23.44, 23.37, 10.82. LRMS (ESI): m/z $[M+H]^+$ Calcd for $C_{51}H_{61}N_{12}O_7^+$: 953.47; found: 953.59. HRMS (ESI-Q-TOF): m/z $[M+H]^+$ Calcd for $C_{51}H_{61}N_{12}O_7^+$: 953.4786; found:953.4785

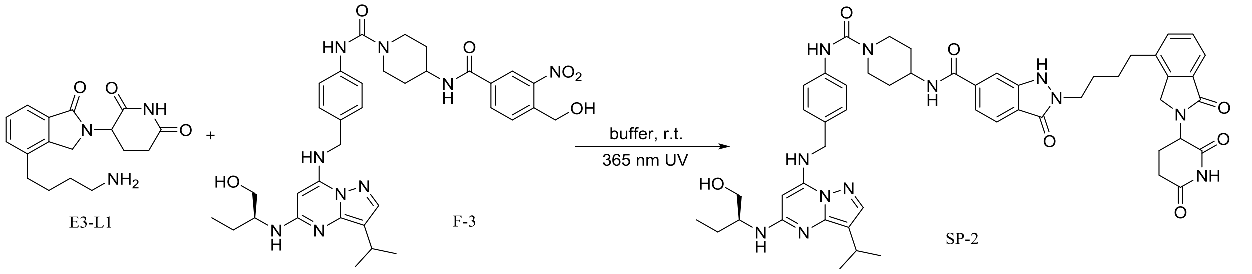

SP-2 was synthesized following the similar procedures as P-1 by using E3-L1 and F-3 to afford white powder. ^{1}H NMR (500 MHz, CD_3OD) δ 7.88 (s, 1H), 7.81 (d, *J* = 8.3 Hz, 1H), 7.72 (s, 1H), 7.62 (dd, *J* = 6.5, 2.1 Hz, 1H), 7.55 (dd, *J* = 8.3, 1.1 Hz, 1H), 7.44 (t, *J* = 6.5 Hz, 2H), 7.39 (d, *J* = 8.6 Hz, 2H), 7.32 (d, *J* = 8.6 Hz, 2H), 5.40 (s, 1H), 5.13 (dd, *J* = 13.4, 5.1 Hz, 1H), 4.63 (s, 2H), 4.43 (d, *J* = 16.9 Hz, 1H), 4.36 (d, *J* = 16.9 Hz, 1H), 4.22 (d, *J* = 13.7 Hz, 2H), 4.15 (dd, *J* = 10.5, 5.0 Hz, 1H), 4.05 (t, *J* = 6.7 Hz, 2H), 3.76 – 3.71 (m, 1H), 3.68 – 3.63 (m, 1H), 3.60 (d, *J* = 4.8 Hz, 1H), 3.08 (d, *J* = 12.3 Hz, 2H), 2.93 – 2.88 (m, 1H), 2.80 – 2.75 (m, 3H), 2.43 (dd, *J* = 13.2, 4.7 Hz, 1H), 2.15 – 2.11 (m, 1H), 2.07 – 2.00 (m, 3H), 1.94 – 1.89 (m, 2H), 1.71 – 1.59 (m, 6H), 1.31 (dd, *J* = 6.9, 3.6 Hz, 6H), 0.99 (t, *J* = 7.4 Hz, 3H).^{13}C NMR (126 MHz, CD_3OD) δ 174.69, 172.17, 171.78, 169.10, 162.03, 157.70, 146.27, 143.63, 141.95, 140.86, 139.41, 138.58, 134.91, 133.37, 132.61, 132.13, 130.85, 129.68, 128.64, 124.27, 122.41, 122.22, 121.11, 119.78, 112.55, 53.56, 48.07, 46.31, 44.52, 44.45, 32.61, 32.38, 32.12, 30.83, 30.74, 30.64, 30.33, 28.79, 28.10, 27.66, 24.97, 24.08, 23.43, 23.35, 10.80. LRMS (ESI): m/z $[M+H]^+$ Calcd for $C_{51}H_{61}N_{12}O_7^+$: 953.47; found: 953.63. HRMS (ESI-Q-TOF): m/z $[M+H]^+$ Calcd for $C_{51}H_{61}N_{12}O_7^+$: 953.4786; found:953.4784

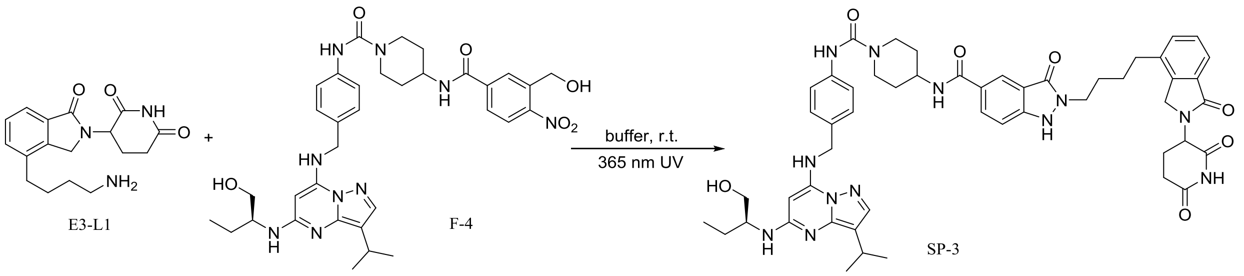

SP-3 was synthesized following the similar procedures as P-1 by using E3-L1 and S-F-4 to afford white powder. ^{1}H NMR (500 MHz, CD_3OD) δ 8.22 (s, 1H), 8.02 (dd, *J* = 8.7, 1.6 Hz, 1H), 7.87 (s, 1H), 7.62 – 7.60 (m, 1H), 7.44 (t, *J* = 6.6 Hz, 2H), 7.39 (d, *J* = 8.5 Hz, 2H), 7.35 – 7.30 (m, 3H), 5.40 (s, 1H), 5.12 (dd, *J* = 13.4, 5.2 Hz, 1H), 4.63 (s, 2H), 4.44 (d, *J* = 17.0 Hz, 1H), 4.38 (d, *J* = 17.0 Hz, 1H), 4.22 (d, *J* = 13.5 Hz, 2H), 4.17 – 4.11 (m, 1H), 4.05 (t, *J* = 6.7 Hz, 2H), 3.73 (td, *J* = 9.8, 5.0 Hz, 1H), 3.69 – 3.62 (m, 1H), 3.62 – 3.58 (m, 1H), 3.07 (d, *J* = 12.2 Hz, 2H), 2.90 (ddd, *J* = 18.6, 13.5, 5.4 Hz, 1H), 2.82 – 2.75 (m, 3H), 2.47 (tt, *J* = 13.3, 6.6 Hz, 1H), 2.16 – 2.10 (m, 1H), 2.05 – 2.01 (m, 3H), 1.90 (dd, *J* = 14.0, 6.9 Hz, 2H), 1.70 – 1.59 (m, 6H), 1.31 (d, *J* = 3.5 Hz, 6H), 0.99 (t, *J* = 7.4 Hz, 3H). ^{13}C NMR (126 MHz, CD_3OD) δ 174.67, 172.19, 171.79, 168.98, 162.29, 157.73, 147.63, 143.63, 141.97, 140.89, 138.56, 133.35, 132.62, 132.46, 132.30, 132.11, 130.85, 130.22, 129.92, 129.68, 128.95, 128.62, 123.93, 122.43, 122.23, 116.92, 112.54, 53.61, 53.15, 48.14, 46.31, 44.57, 44.38, 40.42, 32.73, 32.38, 32.06, 30.84, 30.74, 30.63, 30.61, 30.46, 30.33, 28.79, 28.11, 27.70, 26.93, 24.98, 24.12, 24.08, 23.74, 23.43, 23.35, 14.45, 10.81. LRMS (ESI): m/z $[M+H]^+$ Calcd for $C_{51}H_{61}N_{12}O_7^+$: 953.47; found: 953.56. HRMS (ESI-Q-TOF): m/z $[M+H]^+$ Calcd for $C_{51}H_{61}N_{12}O_7^+$: 953.4786; found:953.4783

1.4.4. Purified compound in this work

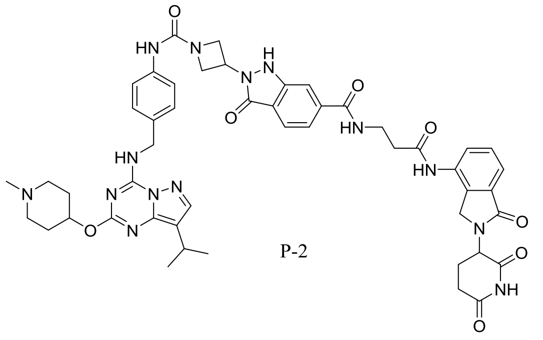

P-2

N-(3-((2-(2,6-dioxopiperidin-3-yl)-1-oxoisoindolin-4-yl)amino)-3-oxopropyl)-2-(1-((4-(((8-isopropyl-2-((1-methylpiperidin-4-yl)oxy)pyrazolo[1,5-a][1,3,5]triazin-4-yl)amino)methyl)phenyl)carbamoyl)azetidin-3-yl)-3-oxo-2,3-dihydro-1H-indazole-6-carboxamide(P-2): E3-L2 (270mg, 0.817mmol, 1.0 equiv.) was dissolved in dry DMF and 2,5-dioxopyrrolidin-1-yl 4-(hydroxymethyl)-3-nitrobenzoate (265 mg, 0.899mmol, 1.1 equiv.) was added. Followed with adding of DIPEA (355μL, 2.04mmol, 2.5 equiv.), the reaction was stirred under room temperature for 1h. The solvant was evaporated and the residue was directly dissolved in 8.17 mL DMSO to acquire 100mM stock. 200μL stock (100mM in DMSO) was mixxed with 800μL of POI-L5 stock (100mM in DMSO) in 9mL of reaction buffer (20mM PBS/ DMSO=1:1, pH=10.5). The reaction was exposed to 365 nm UV for 30 min at room temperature. Water was evaporated and the residue was purified by HPLC to afford the final compound as white powder. ^{1}H NMR (500 MHz, CD_3OD) δ 7.87 (d, *J* = 3.1 Hz, 1H), 7.81 (d, *J* = 8.3 Hz, 1H), 7.75 (s, 1H), 7.67 (d, *J* = 7.9 Hz, 1H), 7.62 (d, *J* = 7.1 Hz, 1H), 7.58 (dd, *J* = 8.3, 1.2 Hz,

1H), 7.49 (t, *J* = 7.7 Hz, 1H), 7.43 (d, *J* = 8.5 Hz, 2H), 7.31 (d, *J* = 8.4 Hz, 2H), 5.44 (t, *J* = 6.9 Hz, 1H), 5.07 (dd, *J* = 13.3, 5.2 Hz, 1H), 4.72 (d, *J* = 3.3 Hz, 2H), 4.47 (dd, *J* = 16.8, 8.8 Hz, 4H), 4.37 (d, *J* = 3.8 Hz, 2H), 3.83 – 3.75 (m, 2H), 3.60 (d, *J* = 12.9 Hz, 1H), 3.48 – 3.38 (m, 2H), 3.38 – 3.32 (m, 2H), 3.23 – 3.17 (m, 1H), 3.08 – 3.04 (m, 1H), 2.92 (d, *J* = 7.2 Hz, 3H), 2.84 – 2.77 (m, 3H), 2.66 – 2.63 (m, 1H), 2.33 (d, *J* = 18.8 Hz, 2H), 2.18 (dd, *J* = 13.7, 8.7 Hz, 1H), 2.06 (dd, *J* = 6.4, 4.2 Hz, 1H), 1.93 – 1.85 (m, 1H), 1.30 (s, 6H). ^{13}C NMR (126 MHz, CD_3OD) δ 174.36, 172.25, 171.96, 171.02, 169.41, 163.41, 160.46, 159.15, 151.91, 151.81, 147.94, 146.47, 145.50, 139.88, 139.83, 136.61, 134.43, 133.90, 133.85, 130.07, 128.87, 128.72, 127.91, 124.71, 121.59, 121.51, 120.44, 115.42, 113.07, 71.25, 67.54, 55.74, 53.69, 53.41, 50.98, 44.83, 44.73, 44.58, 44.05, 43.16, 37.80, 37.12, 32.27, 30.33, 29.63, 28.52, 24.62, 24.51, 24.07, 23.62, 23.59. HRMS (ESI-Q-TOF): m/z $[M+H]^+$ Calcd for $C_{49}H_{55}N_{14}O_8^+$: 967.4327; found: 967.4321.

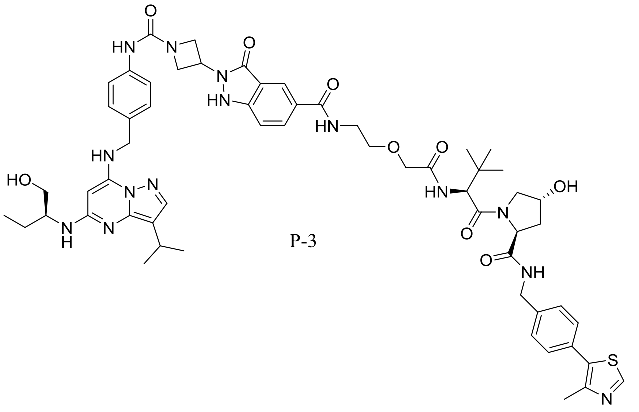

P-3

N-(2-(2-(((S)-1-((2S,4R)-4-hydroxy-2-((4-(4-methylthiazol-5-yl)benzyl)carbamoyl)pyrrolidin-1-yl)-3,3-dimethyl-1-oxobutan-2-yl)amino)-2-oxoethoxy)ethyl)-2-(1-((4-(((5-(((S)-1-hydroxybutan-2-yl)amino)-3-isopropylpyrazolo[1,5-a]pyrimidin-7-yl)amino)methyl)phenyl)carbamoyl)azetidin-3-yl)-3-oxo-2,3-dihydro-1H-indazole-5-carboxamide (P-3): E3-L7 (100mg, 0.188mmol, 1.0 equiv.), 3-(hydroxymethyl)-4-nitrobenzoic acid (41.0mg, 0.207mmol, 1.1 equiv.) and HOBt (28.0mg, 0.207mmol, 1.1 equiv.) was dissolved in dry DMF. After EDCI (40 mg, 0.207mmol, 1.1 equiv.) and DIPEA(82μL, 0.470mmol, 2.5 equiv.) were added, the reaction was stirred under room temperature for 1h. The solvant was evaporated and the residue was directly dissolved in 1.88 mL DMSO to acquire 100mM stock. 200μL stock was mixxed with 800μL of POI-L3 stock (100mM in DMSO) in 9mL of reaction buffer (20mM PBS/ DMSO=1:1, pH=10.5). The reaction was exposed to 365 nm UV for 30 min at room temperature. Water was evaporated and the residue was purified by HPLC to afford the final compound as white powder. ^{1}H NMR (500 MHz, CD_3OD) δ 8.98 (s, 1H), 8.29 (d, *J* = 1.1 Hz, 1H), 8.09 (dd, *J* = 8.7, 1.7 Hz, 1H), 7.87 (s, 1H), 7.47 (d, *J* = 8.6 Hz, 2H), 7.44 (d, *J* = 8.2 Hz, 2H), 7.38 – 7.35 (m, 2H), 7.32 (dd, *J* = 8.7, 2.6 Hz, 3H), 5.40 – 5.36 (m, 1H), 4.71 (s, 1H), 4.63 (dd, *J* = 8.8, 7.8 Hz, 3H), 4.55 (d, *J* = 15.5 Hz, 1H), 4.50 – 4.48 (m, 1H), 4.44 (s, 1H), 4.43 – 4.39 (m, 2H), 4.34 (d, *J* = 15.6 Hz, 1H), 4.10 (d, *J* = 15.2 Hz, 1H), 4.03 (d, *J* = 15.2 Hz, 1H), 3.88 (d, *J* = 11.2 Hz, 1H), 3.82 – 3.68 (m, 5H), 3.66 – 3.58 (m, 4H), 3.04 (dd, *J* = 12.7, 6.0 Hz, 1H), 2.52 – 2.37 (m, 4H), 2.23 (dd, *J* = 13.2, 7.6 Hz, 1H), 2.11 – 2.06 (m, 1H), 1.68 (dd, *J* = 13.7, 7.4 Hz, 1H), 1.58 (dd, *J* = 13.9, 6.8 Hz, 1H), 1.30 (dd, *J* = 6.9, 3.4 Hz, 6H), 1.00 (s, 9H), 0.98 – 0.96 (m, 3H). ^{13}C NMR (126 MHz, CD_3OD) δ 174.40, 171.86, 171.79, 169.40, 163.66, 159.00, 153.29, 149.54, 143.64, 140.43, 140.27, 134.91, 133.07, 132.20, 131.11, 130.42, 130.35, 129.51, 129.39, 128.95, 128.75, 124.21, 121.50, 117.73, 113.21, 71.26, 71.08, 70.71, 60.89, 58.24, 58.16, 55.74, 46.28, 44.45, 43.67, 40.93, 38.93, 37.12, 26.92, 24.97, 24.08, 23.43, 23.36, 15.55, 10.81. HRMS (ESI-Q-TOF): m/z $[M+H]^+$ Calcd for $C_{58}H_{72}N_{14}O_9S^+$: 1141.5406; found: 1141.5399.

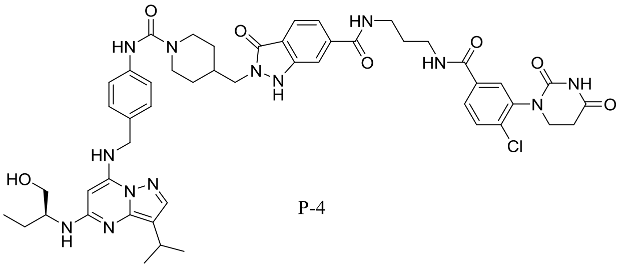

P-4

(S)-N-(3-(4-chloro-3-(2,4-dioxotetrahydropyrimidin-1(2H)-yl)benzamido)propyl)-2-((1-((4-(((5-((1-hydroxybutan-2-yl)amino)-3-isopropylpyrazolo[1,5-a]pyrimidin-7-yl)amino)methyl)phenyl)carbamoyl)piperidin-4-yl)methyl)-3-oxo-2,3-dihydro-1H-indazole-6-carboxamide (P-4) was synthesized following the similar procedures by using POI-L2 and E3-L23 to afford white powder. ^{1}H NMR (500 MHz, CD_3OD) δ 7.93 (s, 1H), 7.86 (s, 1H), 7.82 (d, *J* = 7.5 Hz, 2H), 7.72 (s, 1H), 7.60 (dd, *J* = 18.6, 8.3 Hz, 2H), 7.35 (d, *J* = 8.2 Hz, 2H), 7.30 (d, *J* = 8.2 Hz, 2H), 5.38 (s, 1H), 4.61 (s, 2H), 4.16 (d, *J* = 13.1 Hz, 2H), 3.91 (d, *J* = 7.0 Hz, 2H), 3.79 – 3.57 (m, 5H), 3.50 (s, 4H), 3.06 – 3.00 (m, 1H), 2.91 – 2.82 (m, 4H), 2.23 – 2.17 (m, 1H), 1.94 (dd, *J* = 12.4, 6.4 Hz, 2H), 1.69 (d, *J* = 12.9 Hz, 3H), 1.60 – 1.54 (m, 1H), 1.32 – 1.28 (m, 8H), 0.97 (s, 3H). ^{13}CNMR (126 MHz, CD_3OD) δ 172.64, 169.55, 168.13, 162.28, 157.69, 154.26, 146.31, 143.61, 140.88, 140.08, 139.31, 137.10, 135.87, 134.90, 132.04, 131.54, 130.04, 129.32, 128.61, 124.39, 122.40, 121.04, 119.70, 116.50, 112.39, 50.20, 46.31, 45.99, 45.08, 38.66, 37.02, 32.06, 30.74, 30.09, 24.96, 24.07, 23.42, 23.35, 10.80. HRMS (ESI-Q-TOF): m/z $[M+H]^+$ Calcd for $C_{49}H_{59}ClN_{13}O_7^+$: 976.4349; found: 976.4341.

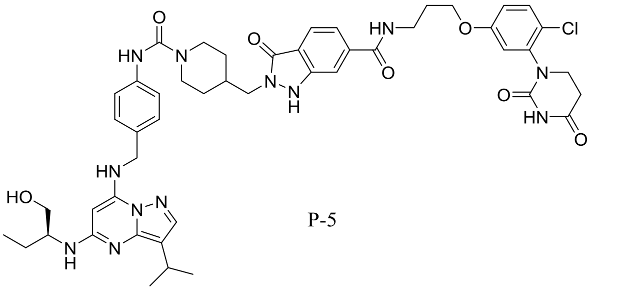

P-5

(S)-N-(3-(4-chloro-3-(2,4-dioxotetrahydropyrimidin-1(2H)-yl)phenoxy)propyl)-2-((1-((4-(((5-((1-hydroxybutan-2-yl)amino)-3-isopropylpyrazolo[1,5-a]pyrimidin-7-yl)amino)methyl)phenyl)carbamoyl)piperidin-4-yl)methyl)-3-oxo-2,3-dihydro-1H-indazole-6-carboxamide (P-5) was synthesized following the similar procedures by using POI-L2 and E3-L21 to afford white powder. ^{1}H NMR (500 MHz, CD_3OD) δ 7.87 (s, 1H), 7.82 (d, *J* = 8.2 Hz, 1H), 7.72 (s, 1H), 7.56 (d, *J* = 8.2 Hz, 1H), 7.41 – 7.28 (m, 5H), 7.03 (d, *J* = 2.2 Hz, 1H), 6.95 (dd, *J* = 8.8, 2.3 Hz, 1H), 5.38 (s, 1H), 4.61 (s, 2H), 4.16 (d, *J* = 13.1 Hz, 2H), 4.10 (t, *J* = 5.6 Hz, 2H), 3.91 (d, *J* = 7.1 Hz, 2H), 3.72 (t, *J* = 6.6 Hz, 3H), 3.59 (d, *J* = 6.5 Hz, 3H), 3.07 – 3.00 (m, 1H), 2.91 – 2.78 (m, 4H), 2.24 – 2.17 (m, 1H), 2.15 – 2.08 (m, 2H), 1.69 (d, *J* = 11.8 Hz, 3H), 1.61 – 1.54 (m, 1H), 1.35 – 1.27 (m, 9H), 0.97 (s, 3H). ^{13}C NMR (126 MHz, CD_3OD) δ 172.75, 169.59, 162.27, 160.01, 157.68, 154.19, 146.30, 143.60, 140.87, 140.42, 139.38, 134.89, 132.04, 131.66, 128.61, 124.73, 124.39, 122.38, 121.02, 119.67, 117.18, 116.89, 112.42, 67.48, 50.18, 46.29, 46.01, 45.06, 38.35, 37.02, 32.02, 30.74, 29.98, 24.96, 24.07, 23.43, 23.35, 10.80. HRMS (ESI-Q-TOF): m/z $[M+H]^+$ Calcd for $C_{48}H_{58}ClN_{12}O_7^+$: 949.4240; found: 949.4235.

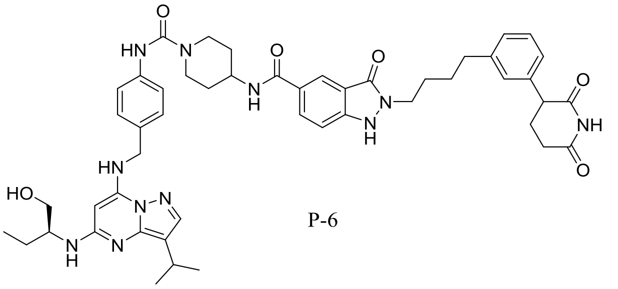

P-6

2-(4-(3-(2,6-dioxopiperidin-3-yl)phenyl)butyl)-N-(1-((4-(((5-(((S)-1-hydroxybutan-2-yl)amino)-3-isopropylpyrazolo[1,5-a]pyrimidin-7-yl)amino)methyl)phenyl)carbamoyl)piperidin-4-yl)-3-oxo-2,3-dihydro-1H-indazole-5-carboxamide (P-6) was synthesized following the similar procedures by using POI-L1 and E3-L20 to afford white powder. ^{1}H NMR (500 MHz, CD_3OD) δ 8.22 (s, 1H), 8.02 (dd, J = 8.7, 1.4 Hz, 1H), 7.88 (s, 1H), 7.39 (d, J = 8.5 Hz, 2H), 7.36 – 7.28 (m, 3H), 7.23 (t, J = 7.5 Hz, 1H), 7.10 (d, J = 7.6 Hz, 1H), 7.04 (d, J = 8.7 Hz, 2H), 5.40 (s, 1H), 4.63 (s, 2H), 4.22 (d, J = 13.4 Hz, 2H), 4.16 – 4.11 (m, 1H), 4.01 (t, J = 6.9 Hz, 2H), 3.79 (dd, J = 10.3, 5.4 Hz, 1H), 3.76 – 3.71 (m, 1H), 3.69 – 3.62 (m, 1H), 3.62 – 3.59 (m, 1H), 3.06 (t, J = 12.0 Hz, 3H), 2.67 (d, J = 7.0 Hz, 4H), 2.58 (dt, J = 17.5, 4.7 Hz, 1H), 2.20 – 2.13 (m, 2H), 2.04 – 2.00 (m, 2H), 1.83 (d, J = 7.5 Hz, 1H), 1.72 – 1.58 (m, 6H), 1.31 (dd, J = 6.9, 4.3 Hz, 6H), 0.99 (t, J = 7.0 Hz, 3H). ^{13}C NMR (126 MHz, CD_3OD) δ 176.62, 175.71, 169.02, 162.12, 157.72, 147.51, 143.72, 140.89, 139.89, 132.46, 132.28, 132.11, 130.85, 129.69, 128.85, 128.63, 128.50, 127.05, 123.84, 122.41, 116.88, 112.51, 46.30, 44.70, 44.57, 40.41, 35.97, 32.73, 32.10, 30.84, 30.75, 30.66, 30.61, 30.47, 30.34, 29.40, 28.74, 28.11, 27.86, 24.98, 24.11, 23.43, 23.35, 10.81. HRMS (ESI-Q-TOF): m/z $[M+H]^+$ Calcd for $C_{49}H_{60}N_{11}O_6^+$: 898.4728; found: 898.4724.

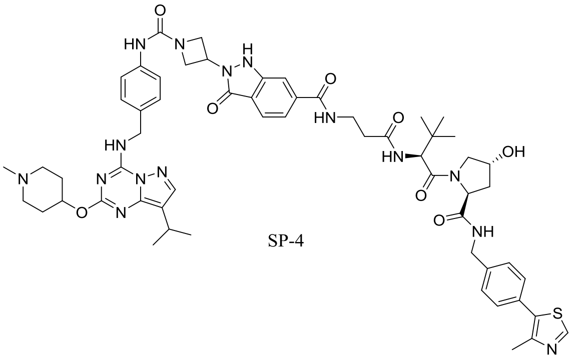

SP-4

N-(3-(((S)-1-((2S,4R)-4-hydroxy-2-((4-(4-methylthiazol-5-yl)benzyl)carbamoyl)pyrrolidin-1-yl)-3,3-dimethyl-1-oxobutan-2-yl)amino)-3-oxopropyl)-2-(1-((4-(((8-isopropyl-2-((1-methylpiperidin-4-yl)oxy)pyrazolo[1,5-a][1,3,5]triazin-4-yl)amino)methyl)phenyl)carbamoyl)azetidin-3-yl)-3-oxo-2,3-dihydro-1H-indazole-6-carboxamide (SP-4)was synthesized following the similar procedures by using POI-L5 and E3-L8 to afford white powder. ^{1}H NMR (500 MHz, CD_3OD) δ 8.93 (s, 1H), 7.86 (d, J = 2.0 Hz, 1H), 7.81 (d, J = 8.3 Hz, 1H), 7.77 (s, 1H), 7.57 (dd, J = 8.2, 1.2 Hz, 1H), 7.43 (d, J = 8.2 Hz, 4H), 7.37 (d, J = 8.2 Hz, 2H), 7.31 (d, J = 8.4 Hz, 2H), 5.50 – 5.41 (m, 1H), 5.39 – 5.33 (m, 1H), 4.72 (d, J = 3.2 Hz, 2H), 4.62 (s, 1H), 4.58 – 4.54 (m, 1H), 4.51 – 4.47 (m, 4H), 4.38 (d, J = 15.4 Hz, 1H), 3.96 (d, J = 10.9 Hz, 1H), 3.80 (dd, J = 11.0, 3.8 Hz, 1H), 3.71 (dd, J = 13.3, 6.6 Hz, 1H), 3.62 (dd, J = 13.5, 6.9 Hz, 2H), 3.43 (dd, J = 8.3, 6.7 Hz, 1H), 3.35 (dd, J = 13.5, 2.2 Hz, 1H), 3.23 – 3.16 (m, 1H), 3.09 – 3.04 (m, 1H), 2.92 (d, J = 7.5 Hz, 3H), 2.61 (t, J = 6.6 Hz, 2H), 2.45 (s, 3H), 2.37 (d, J = 14.6 Hz, 1H), 2.28 (t, J = 14.9 Hz, 2H), 2.21 (dd, J = 9.7, 7.7 Hz, 1H), 2.07 (ddd, J = 21.5, 10.9, 6.3 Hz, 3H), 1.94 – 1.84 (m, 1H), 1.30 (s, 6H), 1.01 (s, 9H). ^{13}CNMR (126 MHz, CD_3OD) δ 174.54, 172.51, 163.55, 160.50, 159.11, 153.09, 151.93, 148.57, 148.00, 146.65, 145.48, 140.39, 140.00, 133.85,

132.46, 131.25, 130.39, 130.35, 129.04, 128.87, 128.74, 124.54, 121.76, 121.47, 120.28, 115.46, 113.14, 71.18, 71.11, 67.43, 61.08, 59.39, 57.98, 55.75, 55.68, 53.69, 51.00, 44.72, 44.50, 44.06, 43.69, 39.00, 37.87, 36.37, 36.17, 28.54, 27.03, 26.94, 24.64, 23.59, 15.62. LRMS (ESI): m/z $[M+H]^+$ Calcd for $C_{58}H_{72}N_{15}O_8S^+$: 1138.53; found: 1138.41. HRMS (ESI-Q-TOF): m/z $[M+H]^+$ Calcd for $C_{58}H_{72}N_{15}O_8S^+$: 1138.5409; found:1138.5404

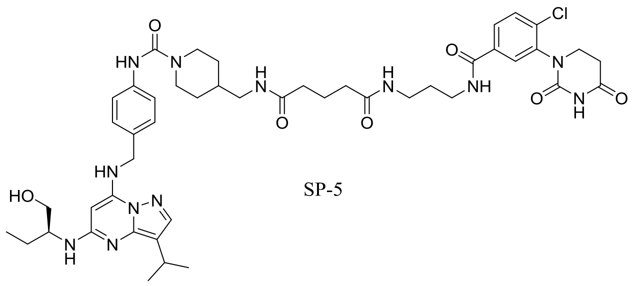

(S)-N^1-(3-(4-chloro-3-(2,4-dioxotetrahydropyrimidin-1(2H)-yl)benzamido)propyl)-N^5-((1-((4-(((5-((1-hydroxybutan-2-yl)amino)-3-isopropylpyrazolo[1,5-a]pyrimidin-7-yl)amino)methyl)phenyl)carbamoyl)piperidin-4-yl)methyl)glutaramide (SP-5) was synthesized following the similar procedures by using POI-L2 and E3-L23 to afford white powder. ^{1}H NMR (500 MHz, CD_3OD) δ 7.90 (d, J = 2.0 Hz, 1H), 7.85 (s, 1H), 7.81 (dd, J = 8.4, 2.0 Hz, 1H), 7.62 (d, J = 8.4 Hz, 1H), 7.35 (d, J = 8.5 Hz, 2H), 7.30 (d, J = 8.5 Hz, 2H), 5.40 (s, 1H), 4.61 (s, 2H), 4.14 (d, J = 13.2 Hz, 2H), 3.81 – 3.55 (m, 5H), 3.49 (t, J = 5.6 Hz, 2H), 3.39 (t, J = 5.6 Hz, 2H), 3.06 (t, J = 8.6 Hz, 3H), 2.89 – 2.80 (m, 4H), 2.20 (dt, J = 11.0, 7.5 Hz, 4H), 1.88 (dd, J = 14.8, 7.4 Hz, 2H), 1.74 – 1.64 (m, 4H), 1.61 – 1.52 (m, 1H), 1.48 – 1.17 (m, 8H), 1.14 (dd, J = 12.1, 10.1 Hz, 2H), 0.96 (t, J = 7.2 Hz, 3H). ^{13}C NMR (126 MHz, CD_3OD) δ 175.87, 175.49, 172.66, 168.29, 157.69, 154.18, 143.55, 140.89, 140.04, 137.09, 135.87, 135.06, 132.03, 131.54, 130.09, 129.41, 128.64, 122.37, 46.29, 45.97, 45.61, 45.28, 40.98, 39.94, 37.56, 36.25, 36.21, 32.06, 30.95, 24.96, 24.01, 23.43, 23.38, 23.22, 10.79. LRMS (ESI): m/z $[M+H]^+$ Calcd for $C_{46}H_{62}ClN_{12}O_7^+$: 929.45; found: 929.58. HRMS (ESI-Q-TOF): m/z $[M+H]^+$ Calcd for $C_{46}H_{62}ClN_{12}O_7^+$: 929.4547; found:929.4549

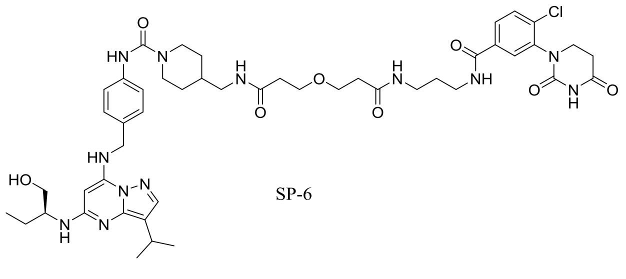

(S)-4-(1-(4-chloro-3-(2,4-dioxotetrahydropyrimidin-1(2H)-yl)phenyl)-1,7,13-trioxo-10-oxa-2,6,14-triazapentadecan-15-yl)-N-(4-(((5-((1-hydroxybutan-2-yl)amino)-3-isopropylpyrazolo[1,5-a]pyrimidin-7-yl)amino)methyl)phenyl)piperidine-1-carboxamide (SP-6) was synthesized following the similar procedures by using POI-L2, E3-L23 and 3-(3-(tert-butoxy)-3-oxopropoxy)propanoic acid (CAS.2086689-08-7) to afford white powder. ^{1}H NMR (500 MHz, CD_3OD) δ 7.90 (d, J = 2.0 Hz, 1H), 7.86 (s, 1H), 7.81 (dd, J = 8.4, 2.1 Hz, 1H), 7.61 (d, J = 8.4 Hz, 1H), 7.35 (d, J = 8.6 Hz, 2H), 7.29 (d, J = 8.5 Hz, 2H), 5.40 (s, 1H), 4.61 (s, 2H), 4.14 (d, J = 13.2 Hz, 2H), 3.67 (qdd, J = 17.1, 12.5, 6.9 Hz, 9H), 3.47 (t, J = 5.7 Hz, 2H), 3.40 (t, J = 5.5 Hz, 2H), 3.09 (d, J = 6.1 Hz, 2H), 3.04 (dd, J = 13.5, 6.8 Hz, 1H), 2.89 – 2.78 (m, 4H), 2.42 (t, J = 6.0 Hz, 2H), 2.38 (t, J = 5.9 Hz, 2H), 1.74 – 1.64 (m, 4H), 1.56 (dd, J = 13.7, 6.9 Hz, 1H), 1.24 (ddd, J = 14.9, 9.5, 2.6 Hz, 8H), 1.16 (dt, J = 22.0, 11.0 Hz, 2H), 0.97 (t, J = 7.2 Hz, 3H). ^{13}C NMR (126 MHz, CD_3OD) δ 174.42, 174.10, 172.63,

168.21, 157.64, 154.15, 143.60, 140.90, 140.06, 137.10, 135.86, 132.02, 131.52, 130.13, 129.44, 128.66, 122.37, 67.92, 67.82, 46.29, 45.97, 45.54, 45.29, 41.05, 39.91, 37.64, 37.44, 32.06, 30.89, 24.97, 24.04, 23.43, 23.36, 10.80. LRMS (ESI): m/z $[M+H]^+$ Calcd for $C_{47}H_{63}ClN_{12}O_8^+$: 959.46; found: 959.63. HRMS (ESI-Q-TOF): m/z $[M+H]^+$ Calcd for $C_{47}H_{64}ClN_{12}O_8^+$: 959.4653; found: 959.4652

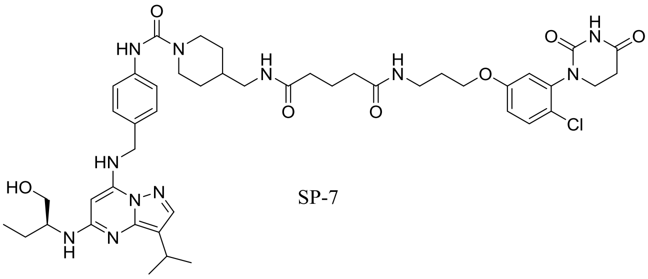

SP-7

(S)-N^1-(3-(4-chloro-3-(2,4-dioxotetrahydropyrimidin-1(2H)-yl)phenoxy)propyl)-N^5-((1-((4-(((5-((1-hydroxybutan-2-yl)amino)-3-isopropylpyrazolo[1,5-a]pyrimidin-7-yl)amino)methyl)phenyl)carbamoyl)piperidin-4-yl)methyl)glutaramide(SP-7): E3-L21 (180mg, 0.605mmol, 1.0 equiv.), 5-(tert-butoxy)-5-oxopentanoic acid (cas.63128-51-8, 125mg, 0.666mmol, 1.1 equiv.) and HOBt (90mg, 0.666mmol, 1.1equiv.) were added into 5mL of dry DMF. After adding of EDCI (127mg, 0.666mmol, 1.1equiv.) and DIPEA (263μL, 1.51mmol, 2.5 equiv.), the reaction was stirred under room-temperature overnight. The solvent was evaporated and the residue purified by column chromatography. 60mg of the intermediate was dissolved in 2mL of 1,4-dioxane. 2mL HCl-1,4-dioxane was added and the reaction was stirred for 1h. The solvent was removed. 50mg POI-L2 (0.121mmol, 1.0equiv.) and 93mg HATU (0.242mmol, 2.0equiv.) were mixxed into dry DMF following DIPEA (53μL, 0.303mmol, 2.5equiv.) was added. The reaction was stirred under room-temperature for 2h. The solvent was evaporated and the residue was purified by HPLC to afford white powder. ^{1}H NMR (500 MHz, CD_3OD) δ 7.87 (s, 1H), 7.39 (d, *J* = 8.9 Hz, 1H), 7.36 (d, *J* = 8.6 Hz, 2H), 7.30 (d, *J* = 8.6 Hz, 2H), 7.04 (d, *J* = 2.9 Hz, 1H), 6.94 (dd, *J* = 8.9, 2.9 Hz, 1H), 5.39 (s, 1H), 4.61 (s, 2H), 4.15 (d, *J* = 13.3 Hz, 2H), 4.01 (t, *J* = 6.1 Hz, 2H), 3.79 – 3.70 (m, 3H), 3.59 (dd, *J* = 11.0, 6.1 Hz, 2H), 3.34 (t, *J* = 6.8 Hz, 2H), 3.08 (d, *J* = 6.2 Hz, 2H), 3.04 (dd, *J* = 13.7, 6.8 Hz, 1H), 2.84 (dt, *J* = 13.3, 9.5 Hz, 4H), 2.21 (t, *J* = 7.0 Hz, 4H), 1.98 – 1.93 (m, 2H), 1.87 (dd, *J* = 14.9, 7.4 Hz, 2H), 1.75 – 1.65 (m, 4H), 1.57 (dt, *J* = 13.9, 6.9 Hz, 1H), 1.31 (d, *J* = 3.1 Hz, 3H), 1.29 (d, *J* = 3.1 Hz, 3H), 1.21 – 1.13 (m, 2H), 0.97 (t, *J* = 7.3 Hz, 3H). ^{13}C NMR (126 MHz, CD_3OD) δ 175.49, 175.43, 172.79, 160.00, 157.70, 154.16, 143.58, 140.92, 140.45, 135.03, 132.02, 131.65, 128.63, 124.74, 122.37, 117.16, 116.95, 67.26, 46.30, 46.02, 45.62, 45.29, 37.61, 37.33, 36.27, 32.04, 30.98, 30.03, 24.97, 24.07, 23.43, 23.36, 10.80. LRMS (ESI): m/z $[M+H]^+$ Calcd for $C_{45}H_{61}ClN_{11}O_7^+$: 902.44; found: 902.59. HRMS (ESI-Q-TOF): m/z $[M+H]^+$ Calcd for $C_{45}H_{61}ClN_{11}O_7^+$: 902.4433; found: 902.4438

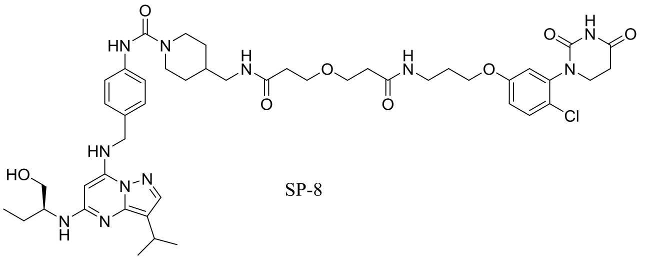

SP-8

(S)-4-((3-(3-((3-(4-chloro-3-(2,4-dioxotetrahydropyrimidin-1(2H)-yl)phenoxy)propyl)amino)-3-oxopropoxy)propanamido)methyl)-N-(4-(((5-((1-hydroxybutan-2-yl)amino)-3-isopropylpyrazolo[1,5-a]pyrimidin-7-yl)amino)methyl)phenyl)piperidine-1-carboxamide (SP-8) was synthesized following the similar procedures by using

POI-L2, E3-L21 and 3-(3-(tert-butoxy)-3-oxopropoxy)propanoic acid (CAS.2086689-08-7) to afford white powder. ^{1}H NMR (500 MHz, CD_3OD) δ 7.87 (s, 1H), 7.36 (dd, *J* = 8.4, 6.3 Hz, 3H), 7.30 (d, *J* = 8.4 Hz, 2H), 7.04 (d, *J* = 2.4 Hz, 1H), 6.93 (dd, *J* = 8.9, 2.8 Hz, 1H), 5.40 (s, 1H), 4.61 (s, 2H), 4.14 (d, *J* = 13.1 Hz, 2H), 4.00 (t, *J* = 6.1 Hz, 2H), 3.76 – 3.65 (m, 8H), 3.59 (dd, *J* = 10.9, 6.0 Hz, 1H), 3.34 (t, *J* = 6.8 Hz, 2H), 3.10 (d, *J* = 5.6 Hz, 2H), 3.03 (dd, *J* = 13.0, 6.5 Hz, 1H), 2.88 – 2.80 (m, 4H), 2.41 (dd, *J* = 10.7, 5.6 Hz, 4H), 1.96 (dd, *J* = 12.6, 6.2 Hz, 2H), 1.70 (dd, *J* = 21.5, 8.6 Hz, 4H), 1.61 – 1.54 (m, 1H), 1.31 (d, *J* = 3.1 Hz, 3H), 1.29 (d, *J* = 3.0 Hz, 3H), 1.19 – 1.13 (m, 2H), 0.97 (t, *J* = 7.1 Hz, 3H). ^{13}C NMR (126 MHz, CD_3OD) δ 174.08, 174.00, 172.77, 160.00, 157.64, 154.16, 143.61, 140.94, 140.44, 132.01, 131.65, 128.66, 124.73, 122.33, 117.20, 116.95, 67.97, 67.90, 67.26, 46.30, 46.01, 45.56, 45.29, 37.64, 37.49, 37.35, 32.02, 30.92, 30.06, 24.97, 24.06, 23.43, 23.36, 10.81. LRMS (ESI): m/z $[M+H]^+$ Calcd for $C_{46}H_{63}ClN_{11}O_8^+$: 932.45; found: 932.56. HRMS (ESI-Q-TOF): m/z $[M+H]^+$ Calcd for $C_{46}H_{63}ClN_{11}O_8^+$: 932.4550; found:932.4544

^{1}H NMR and ^{13}C NMR Spectra

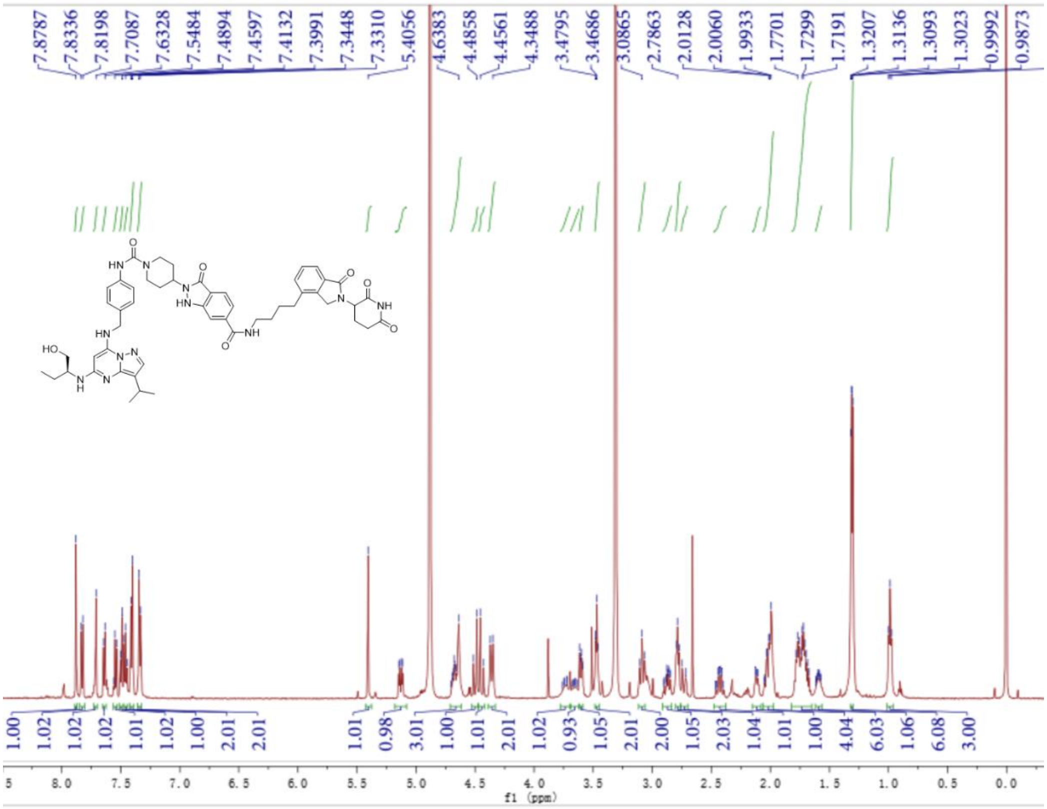

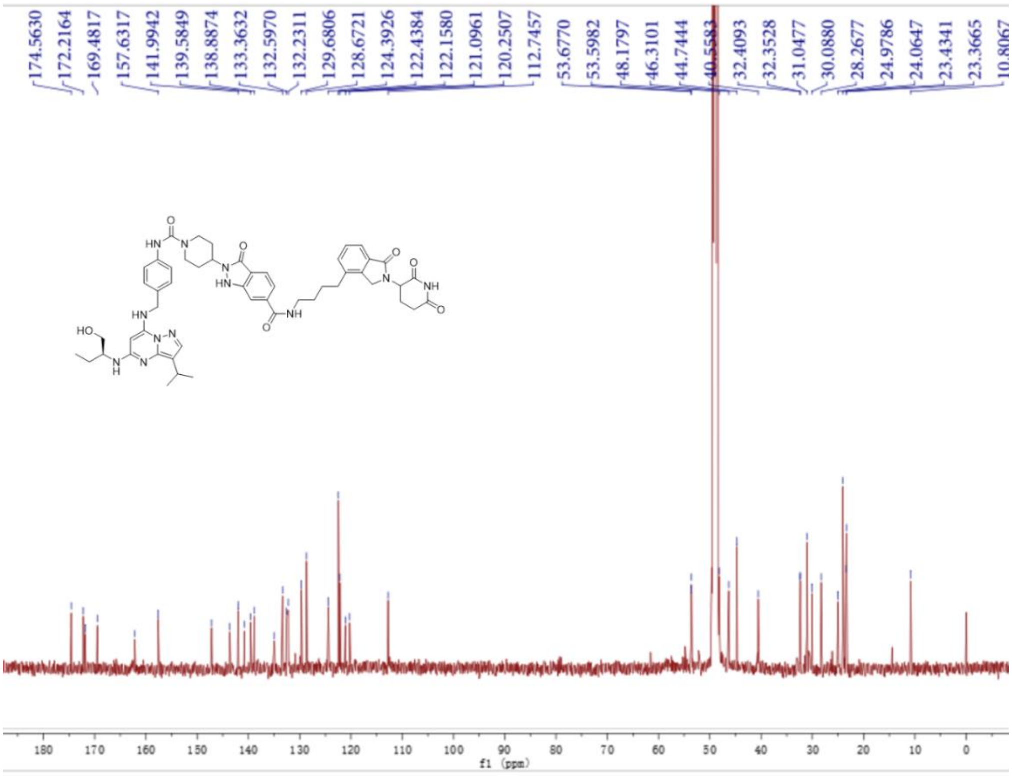

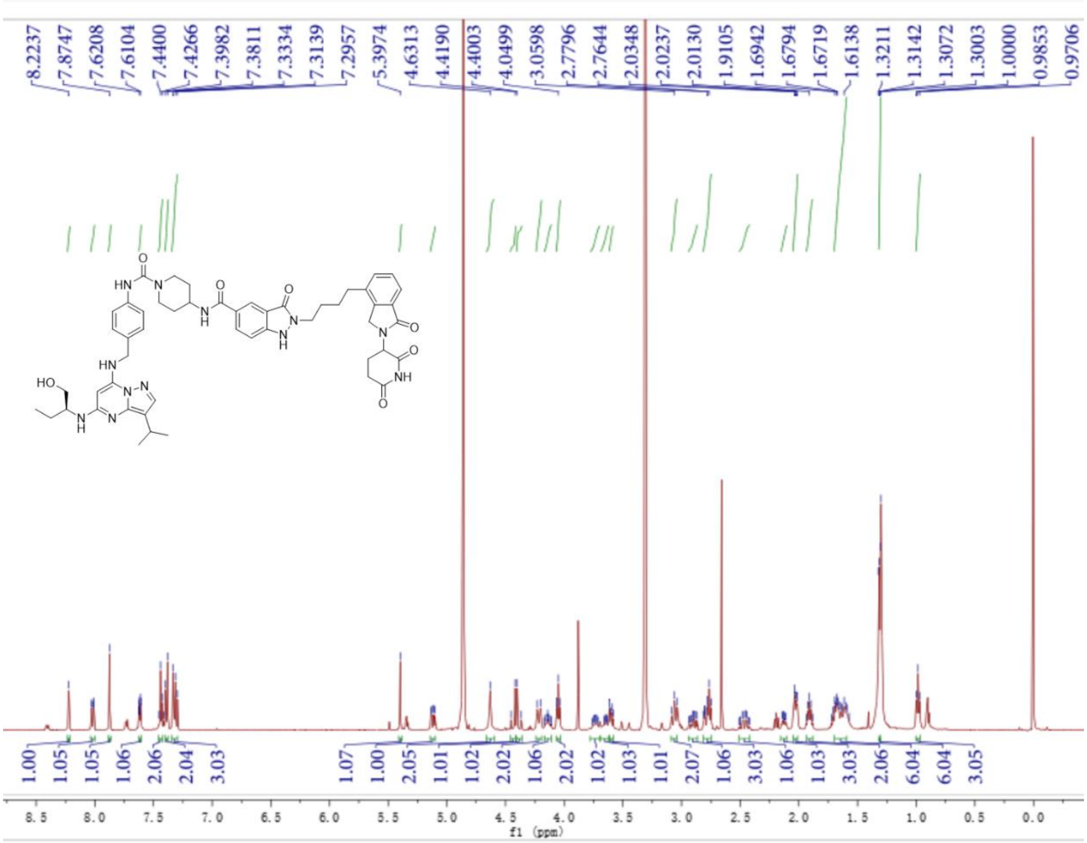
8.2237 7.8747 7.6208 7.6104 7.4400 7.4266 7.3982 7.3811 7.3334 7.3139 7.2957 5.3974 4.6313 4.4190 4.4003 4.0499 3.0598 2.7796 2.7644 2.0348 2.0237 2.0130 1.9105 1.6942 1.6794 1.6719 1.6138 1.3211 1.3142 1.3072 1.3003 1.0000 0.9853 0.9706
HN O N N H O N O N H N
HN N N
HO N H N
O N O NH O
1.00 1.05 1.05 1.06 2.06 2.04 3.03 1.07 1.00 2.05 1.01 1.02 2.02 1.06 2.02 1.02 1.03 1.01 2.07 1.06 3.03 1.06 1.03 3.03 2.06 6.04 6.04 3.05
8.5 8.0 7.5 7.0 6.5 6.0 5.5 5.0 4.5 4.0 3.5 3.0 2.5 2.0 1.5 1.0 0.5 0.0
f1 (ppm)

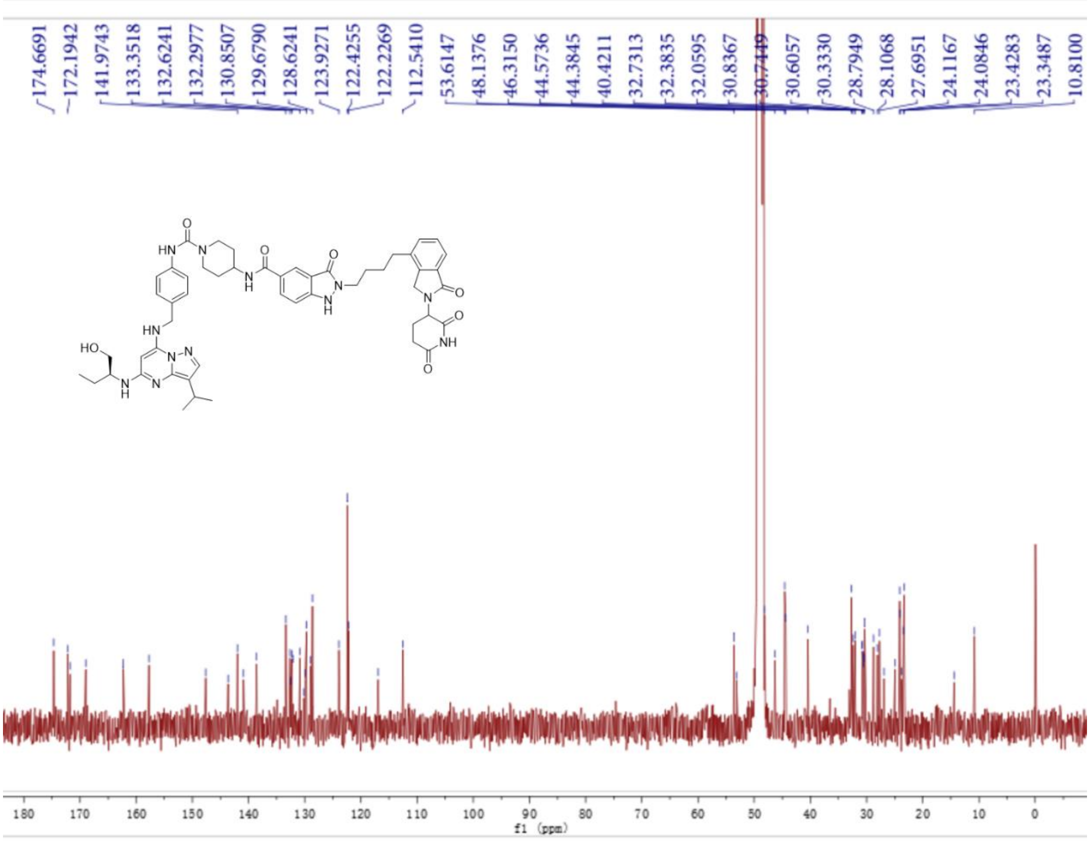
174.6691 172.1942 141.9743 133.3518 132.6241 132.2977 130.8507 129.6790 128.6241 123.9271 122.4255 122.2269 112.5410 53.6147 48.1376 46.3150 44.5736 44.3845 40.4211 32.7313 32.3835 32.0595 30.8367 30.7449 30.6057 30.3330 28.7949 28.1068 27.6951 24.1167 24.0846 23.4283 23.3487 10.8100
HN O N N H O N O N H N
HN N N
HO N H N
O N O NH O
180 170 160 150 140 130 120 110 100 90 80 70 60 50 40 30 20 10 0
f1 (ppm)

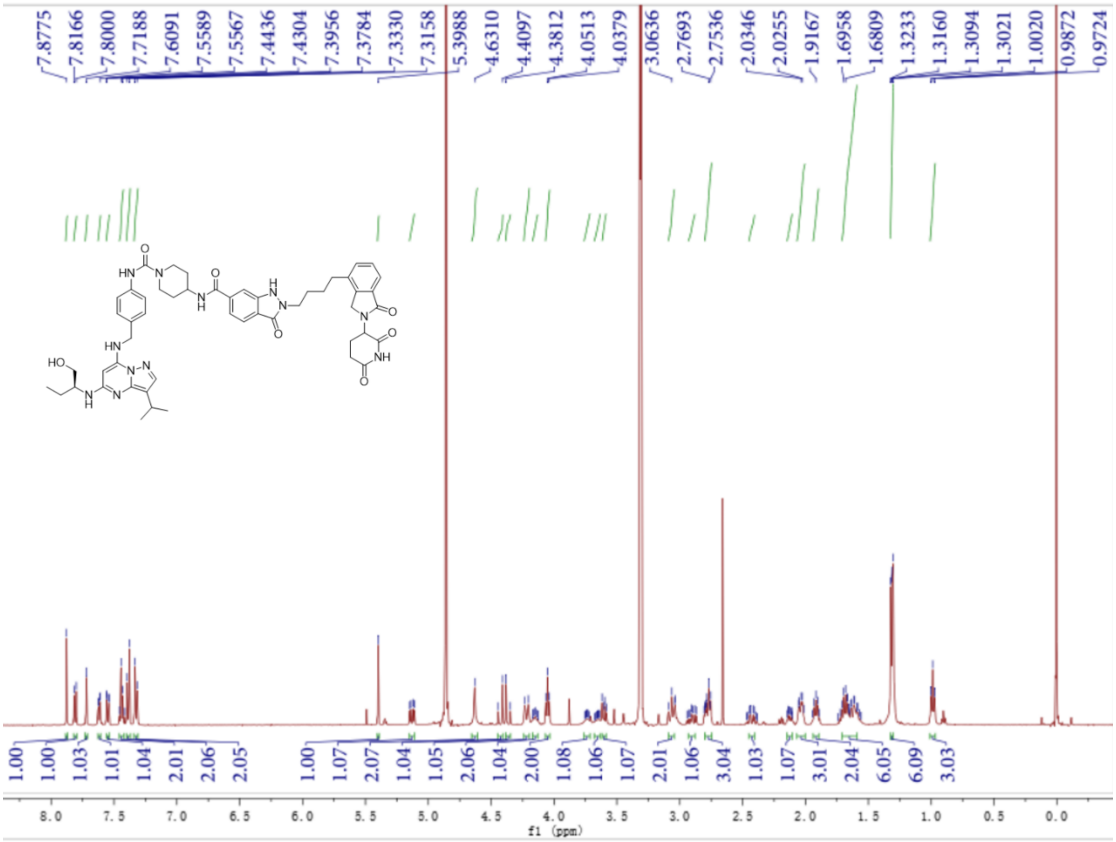

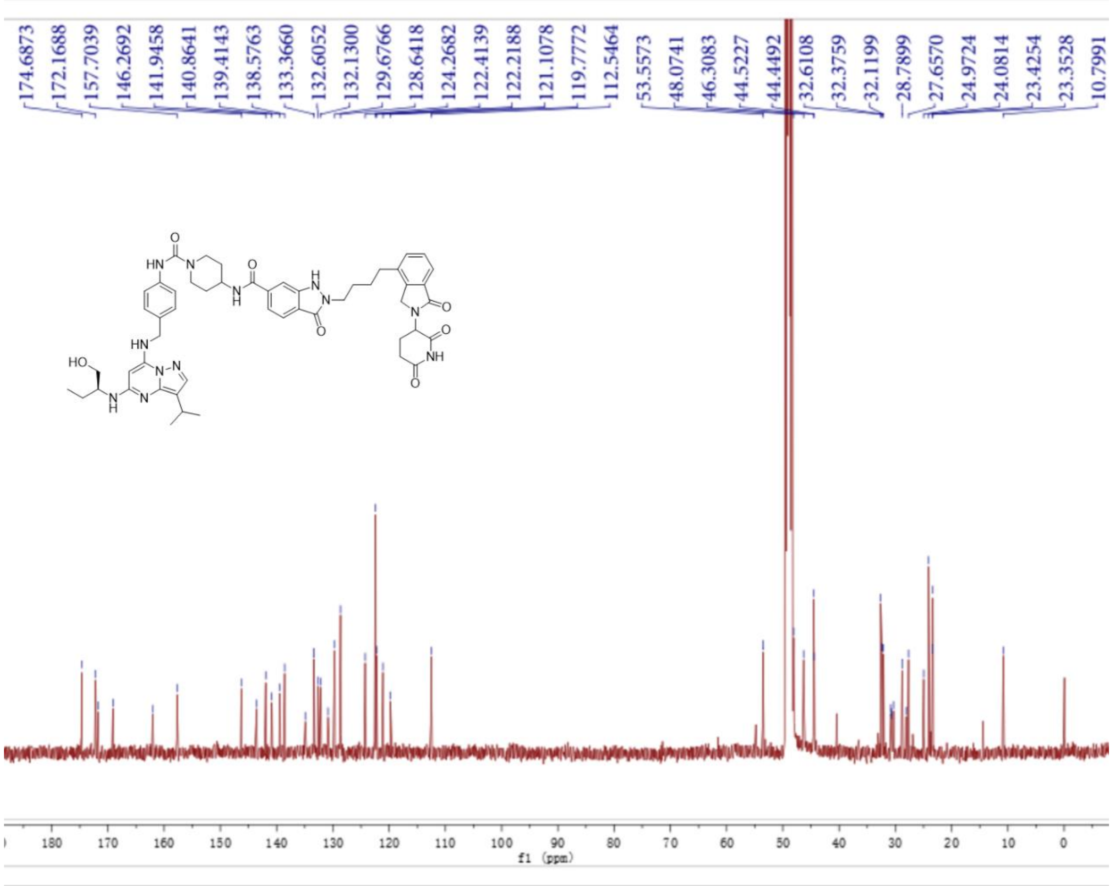

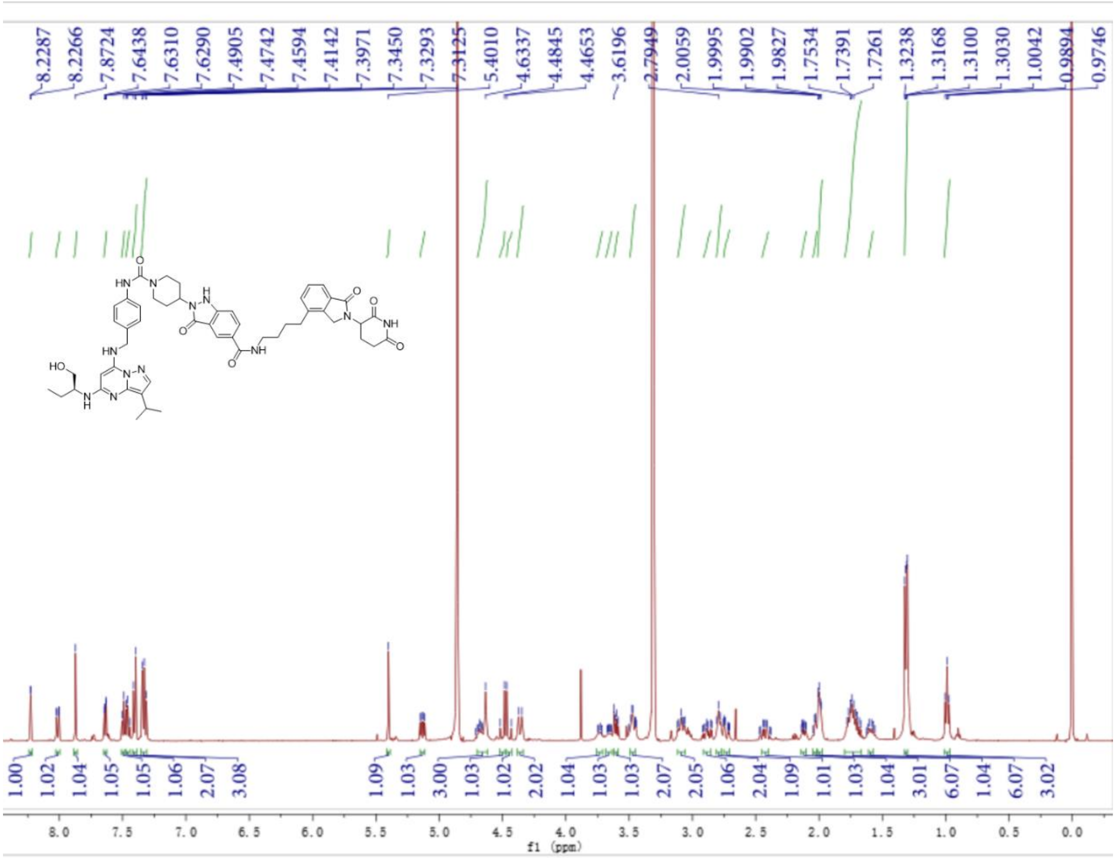
8.2287
8.2266
7.8724
7.6438
7.6310
7.6290
7.4905
7.4742
7.4594
7.4142
7.3971
7.3450
7.3293
7.3125
5.4010
4.6337
4.4845
4.4653
3.6196
2.7949
2.0059
1.9995
1.9902
1.9827
1.7534
1.7391
1.7261
1.3238
1.3168
1.3100
1.3030
1.0042
0.9894
0.9746
f1 (ppm)

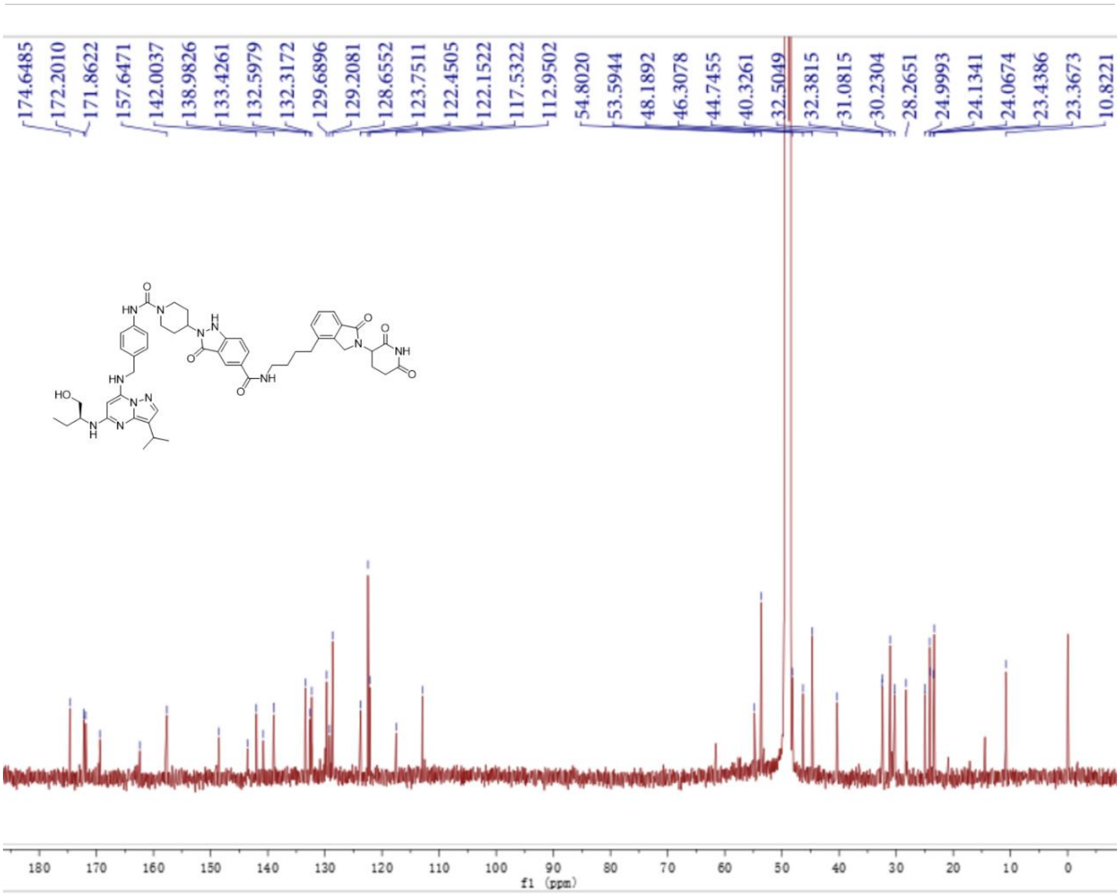
174.6485
172.2010
171.8622
157.6471
142.0037
138.9826
133.4261
132.5979
132.3172
129.6896
129.2081
128.6552
123.7511
122.4505
122.1522
117.5322
112.9502
54.8020
53.5944
48.1892
46.3078
44.7455
40.3261
32.5049
32.3815
31.0815
30.2304
28.2651
24.9993
24.1341
24.0674
23.4386
23.3673
10.8221
f1 (ppm)

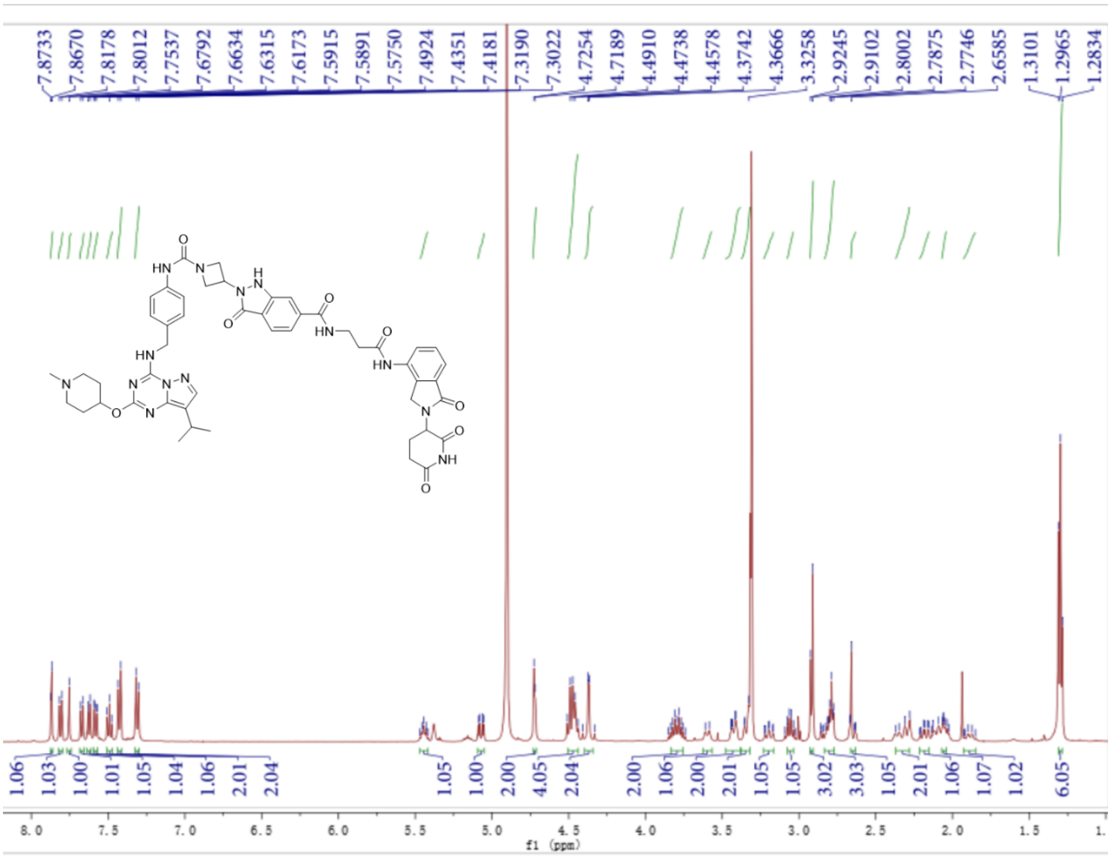
7.8733
7.8670
7.8178
7.8012
7.7537
7.6792
7.6634
7.6315
7.6173
7.5915
7.5891
7.5750
7.4924
7.4351
7.4181
7.3190
7.3022
4.7254
4.7189
4.4910
4.4738
4.4578
4.3742
4.3666
3.3258
2.9245
2.9102
2.8002
2.7875
2.7746
2.6585
1.3101
1.2965
1.2834
1.06
1.03
1.00
1.01
1.05
1.04
1.06
2.01
2.04
1.05
1.00
2.00
4.05
2.04
2.00
1.06
2.00
2.01
1.05
1.05
3.02
3.03
1.05
2.01
1.06
1.07
1.02
6.05
8.0 7.5 7.0 6.5 6.0 5.5 5.0 4.5 4.0 3.5 3.0 2.5 2.0 1.5 1.
f1 (ppm)

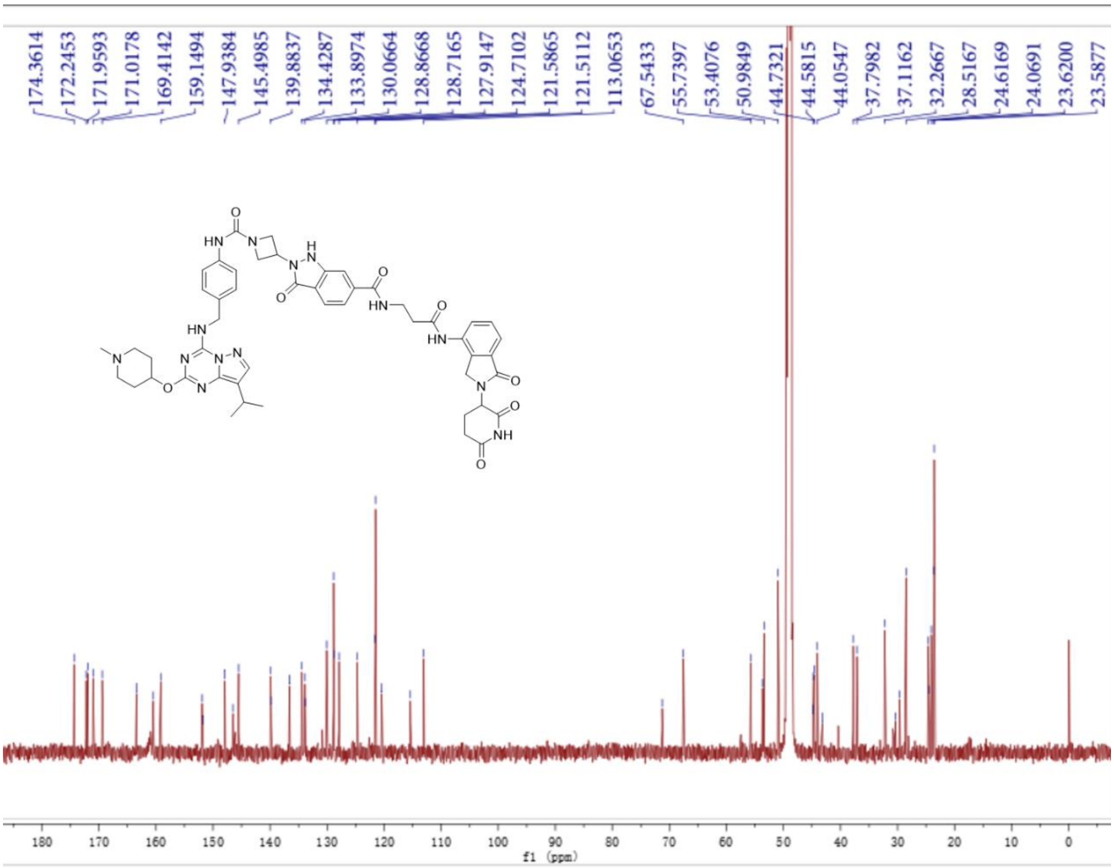
174.3614
172.2453
171.9593
171.0178
169.4142
159.1494
147.9384
145.4985
139.8837
134.4287
133.8974
130.0664
128.8668
128.7165
127.9147
124.7102
121.5865
121.5112
113.0653
67.5433
55.7397
53.4076
50.9849
44.7321
44.5815
44.0547
37.7982
37.1162
32.2667
28.5167
24.6169
24.0691
23.6200
23.5877
180 170 160 150 140 130 120 110 100 90 80 70 60 50 40 30 20 10 0
f1 (ppm)

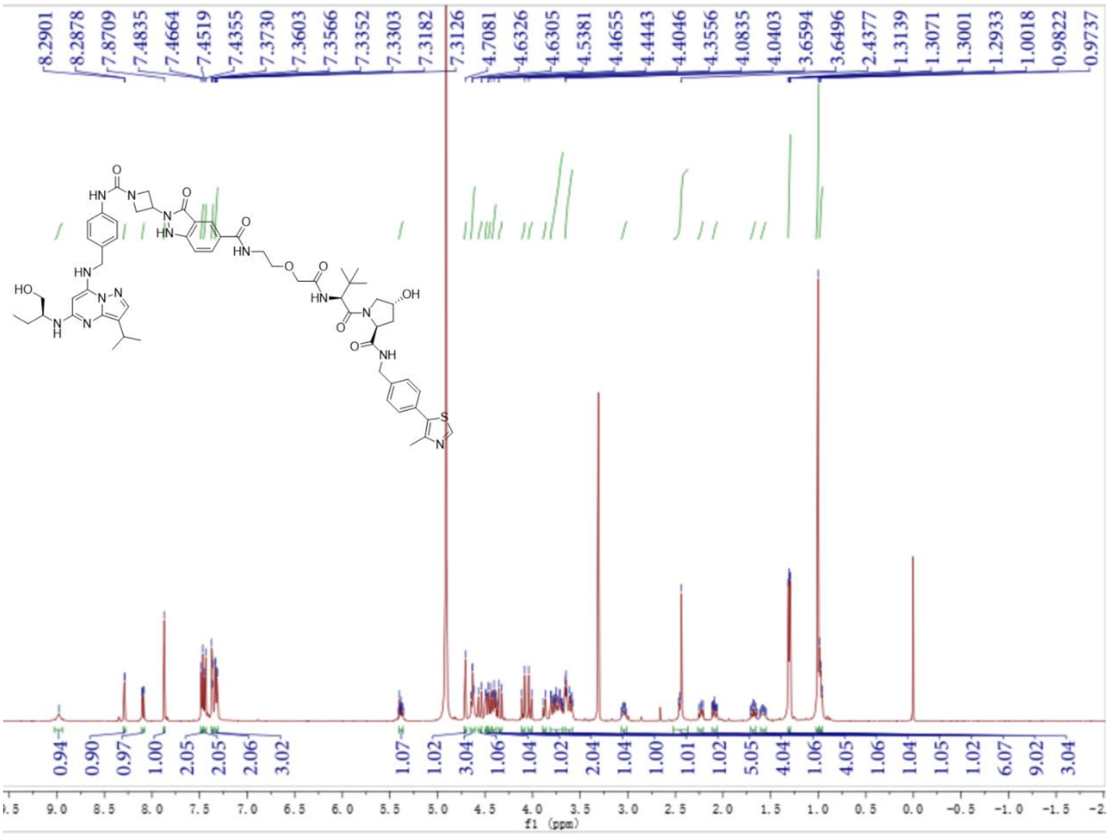
8.2901
8.2878
7.8709
7.4835
7.4664
7.4519
7.4355
7.3730
7.3603
7.3566
7.3352
7.3303
7.3182
7.3126
4.7081
4.6326
4.6305
4.5381
4.4655
4.4443
4.4046
4.3556
4.0835
4.0403
3.6594
3.6496
2.4377
1.3139
1.3071
1.3001
1.2933
1.0018
0.9822
0.9737
0.94
0.90
0.97
1.00
2.05
2.05
2.06
3.02
1.07
1.02
3.04
1.06
1.04
1.02
2.04
1.04
1.00
1.01
1.02
5.05
4.04
1.06
4.05
1.06
1.04
1.05
1.02
6.07
9.02
3.04
f1 (ppm)

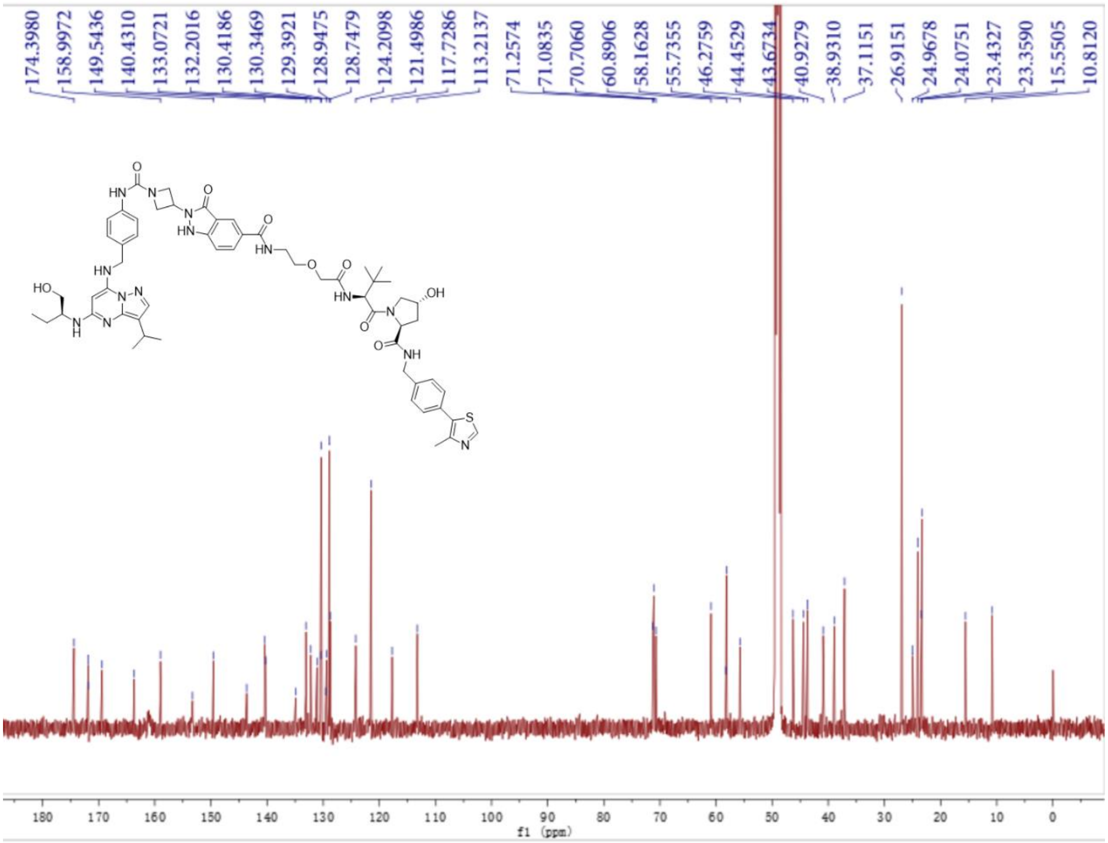
174.3980
158.9972
149.5436
140.4310
133.0721
132.2016
130.4186
130.3469
129.3921
128.9475
128.7479
124.2098
121.4986
117.7286
113.2137
71.2574
71.0835
70.7060
60.8906
58.1628
55.7355
46.2759
44.4529
43.6734
40.9279
38.9310
37.1151
26.9151
24.9678
24.0751
23.4327
23.3590
15.5505
10.8120
f1 (ppm)

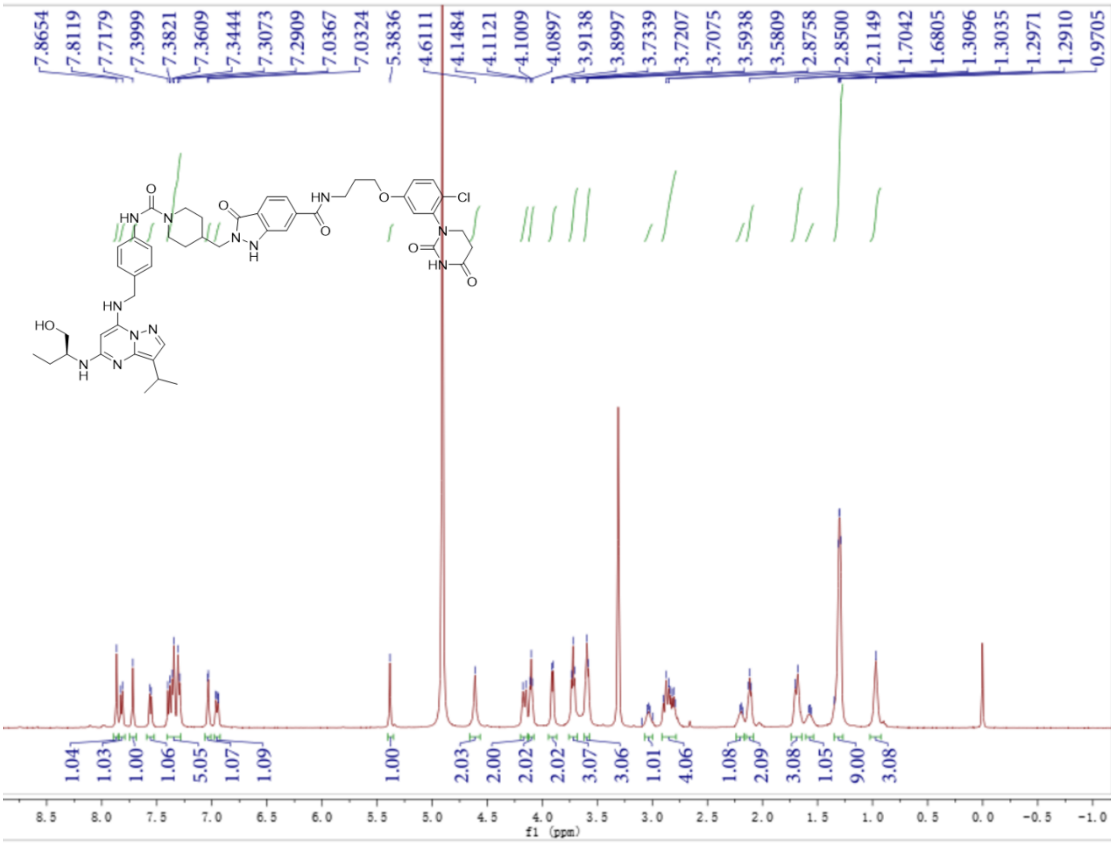

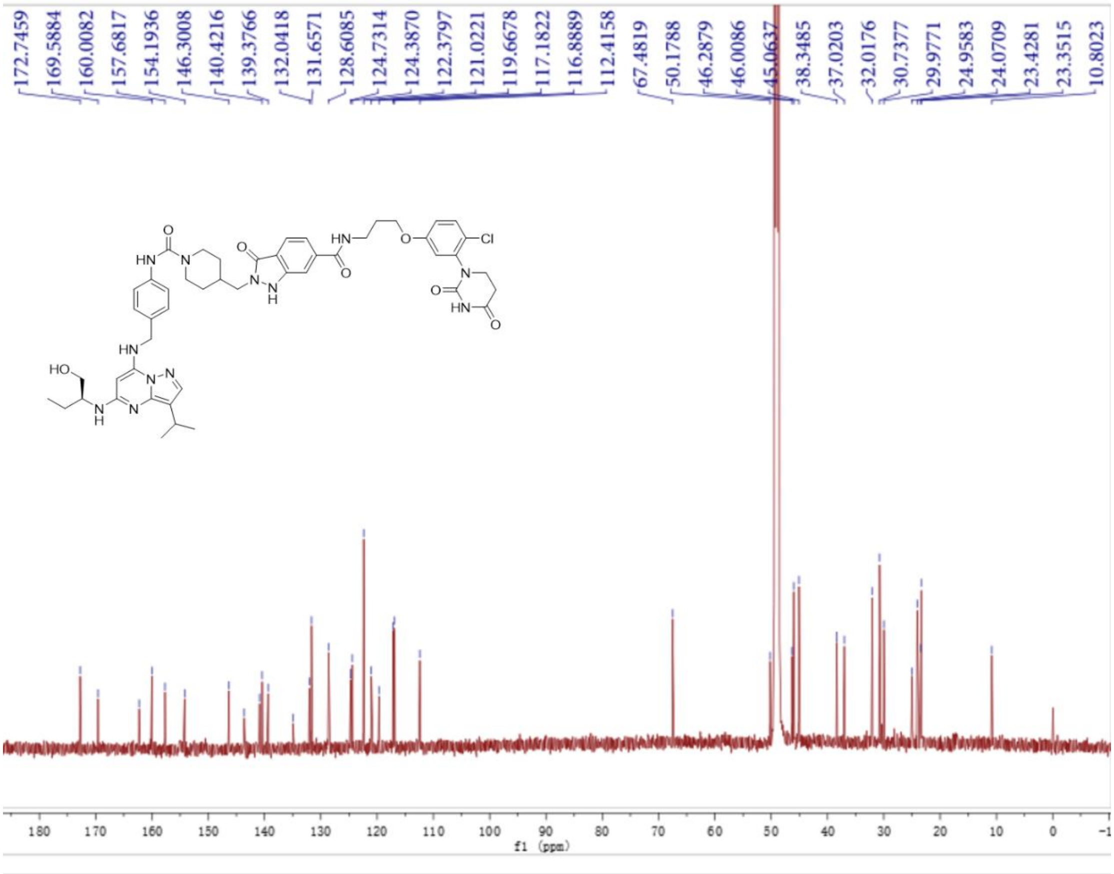

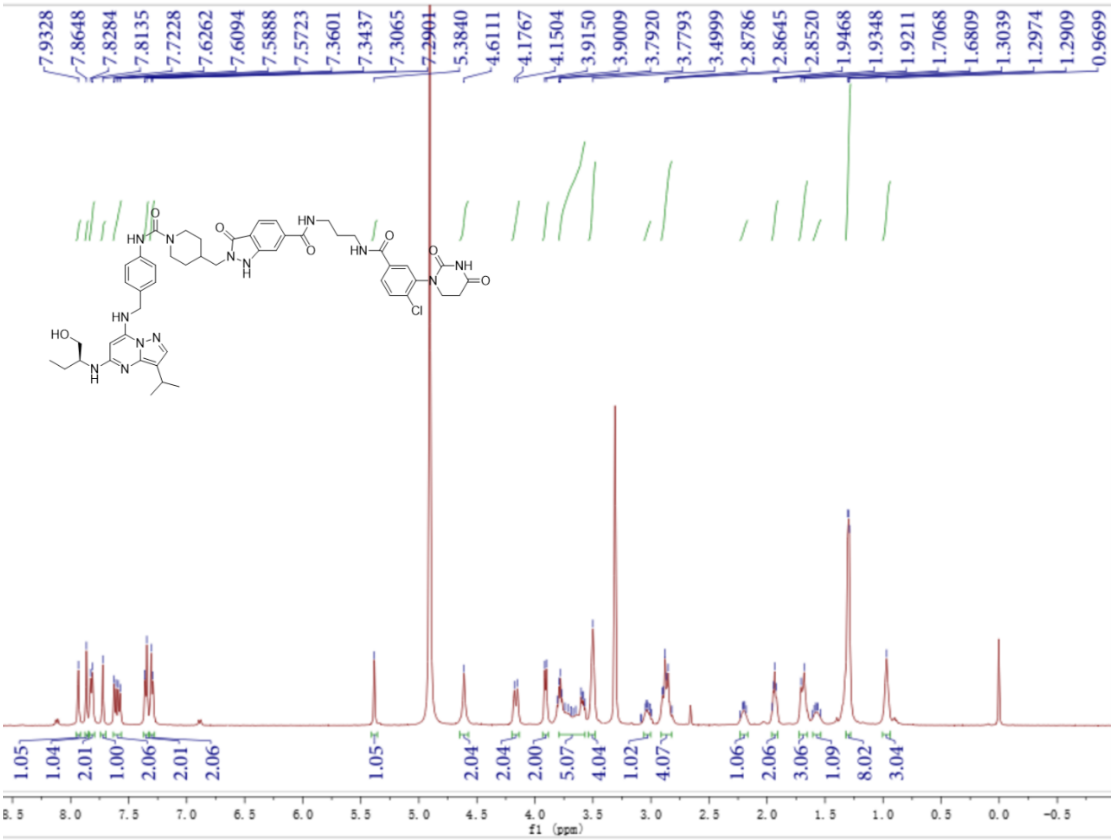

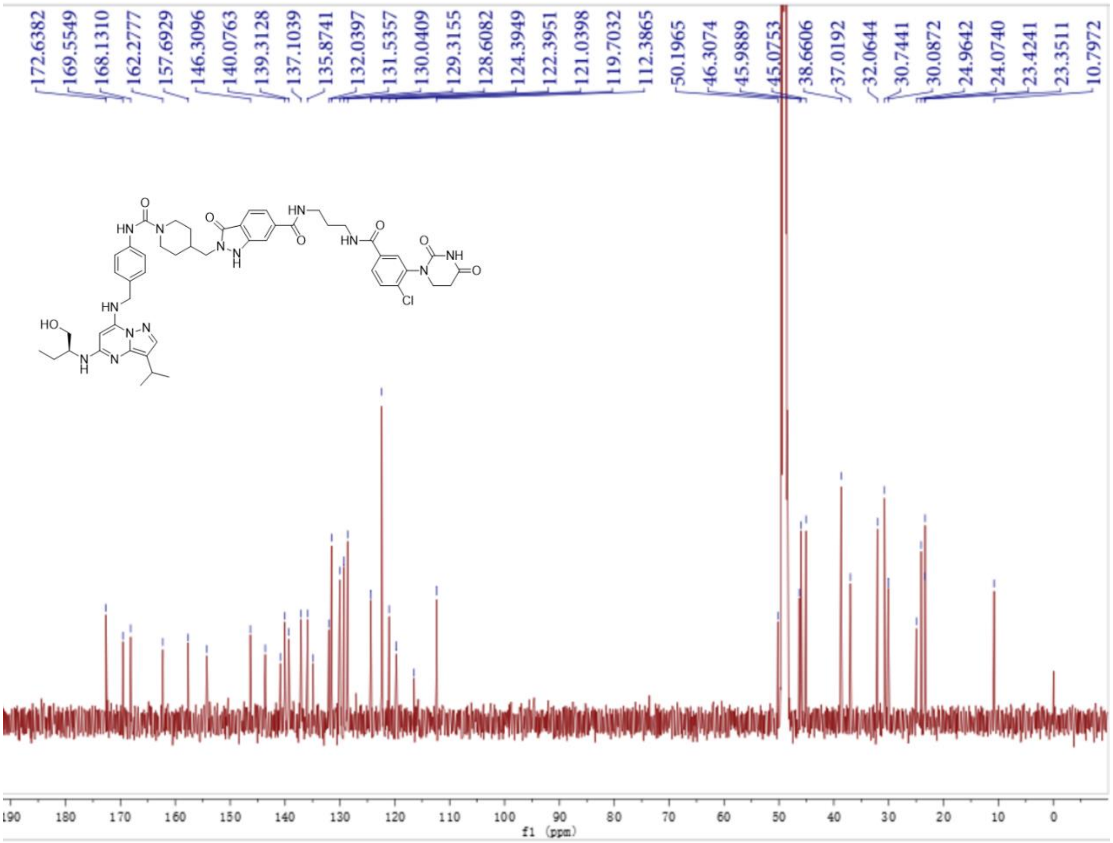

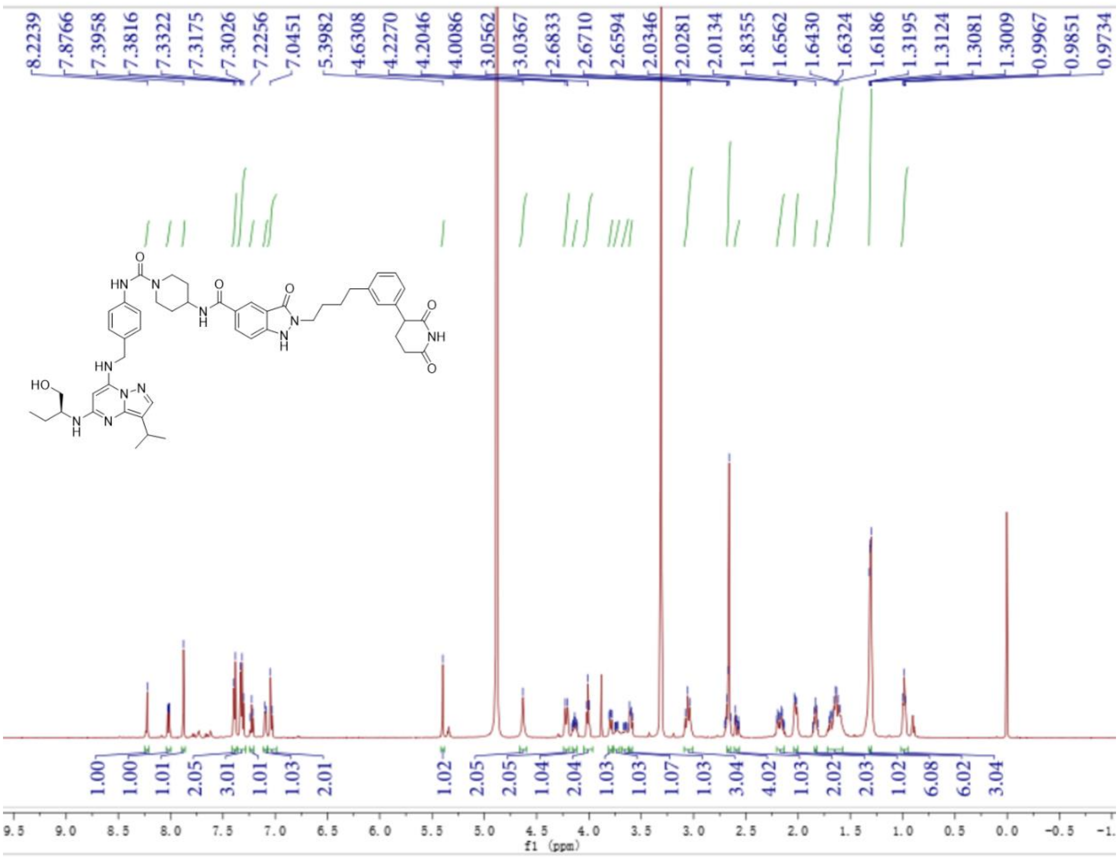
8.2239
7.8766
7.3958
7.3816
7.3322
7.3175
7.3026
7.2256
7.0451
5.3982
4.6308
4.2270
4.2046
4.0086
3.0562
3.0367
2.6833
2.6710
2.6594
2.0346
2.0281
2.0134
1.8355
1.6562
1.6430
1.6324
1.6186
1.3195
1.3124
1.3081
1.3009
0.9967
0.9851
0.9734
HN
O
N
O
N
H
O
N
N
H
HN
HO
N
H
N
N
N
N
O
NH
O
1.00
1.00
1.01
2.05
3.01
1.01
1.03
2.01
1.02
2.05
2.05
1.04
2.04
1.03
1.03
1.07
1.03
3.04
4.02
1.03
2.02
2.03
1.02
6.08
6.02
3.04
f1 (ppm)

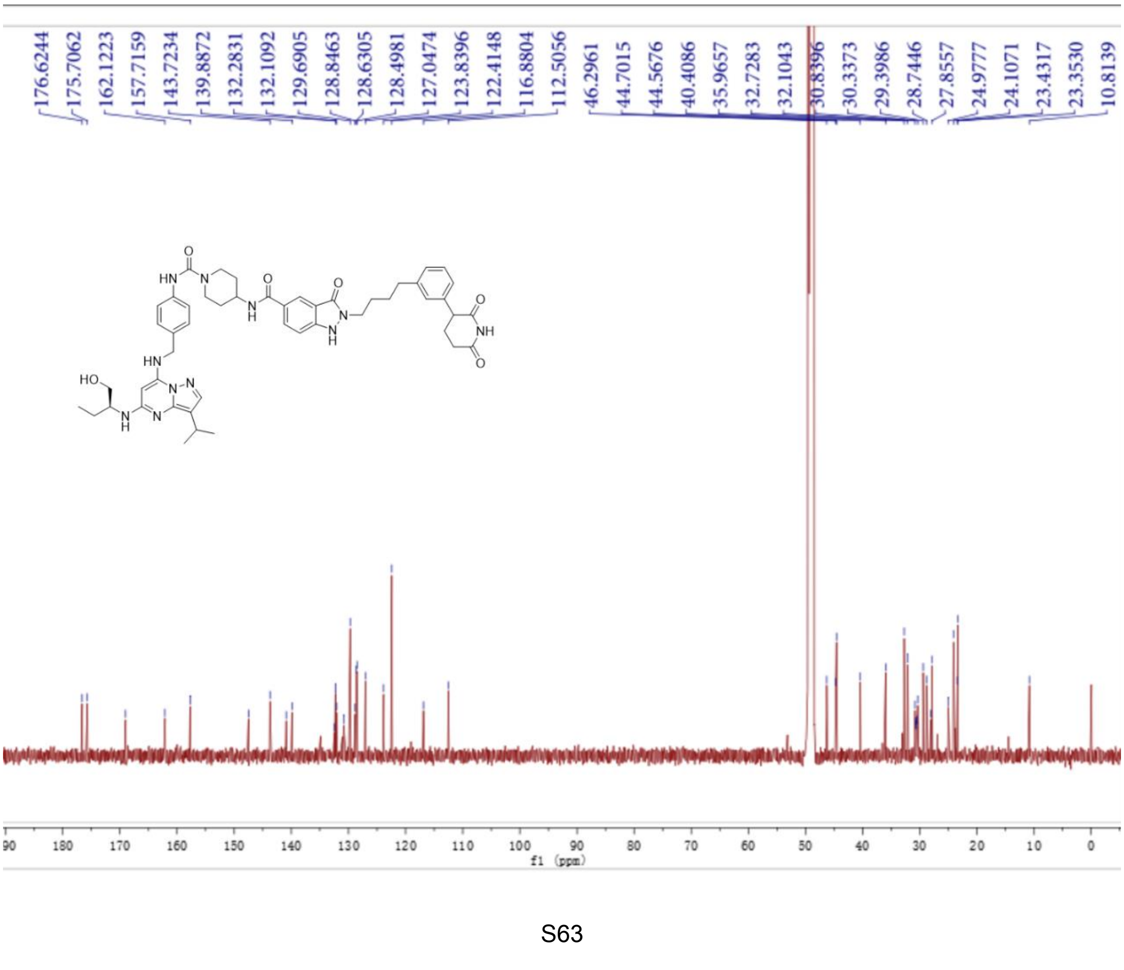
176.6244
175.7062
162.1223
157.7159
143.7234
139.8872
132.2831
132.1092
129.6905
128.8463
128.6305
128.4981
127.0474
123.8396
122.4148
116.8804
112.5056
46.2961
44.7015
44.5676
40.4086
35.9657
32.7283
32.1043
30.8396
30.3373
29.3986
28.7446
27.8557
24.9777
24.1071
23.4317
23.3530
10.8139
HN
O
N
O
N
H
O
N
N
H
HN
HO
N
H
N
N
N
N
O
NH
O
f1 (ppm)

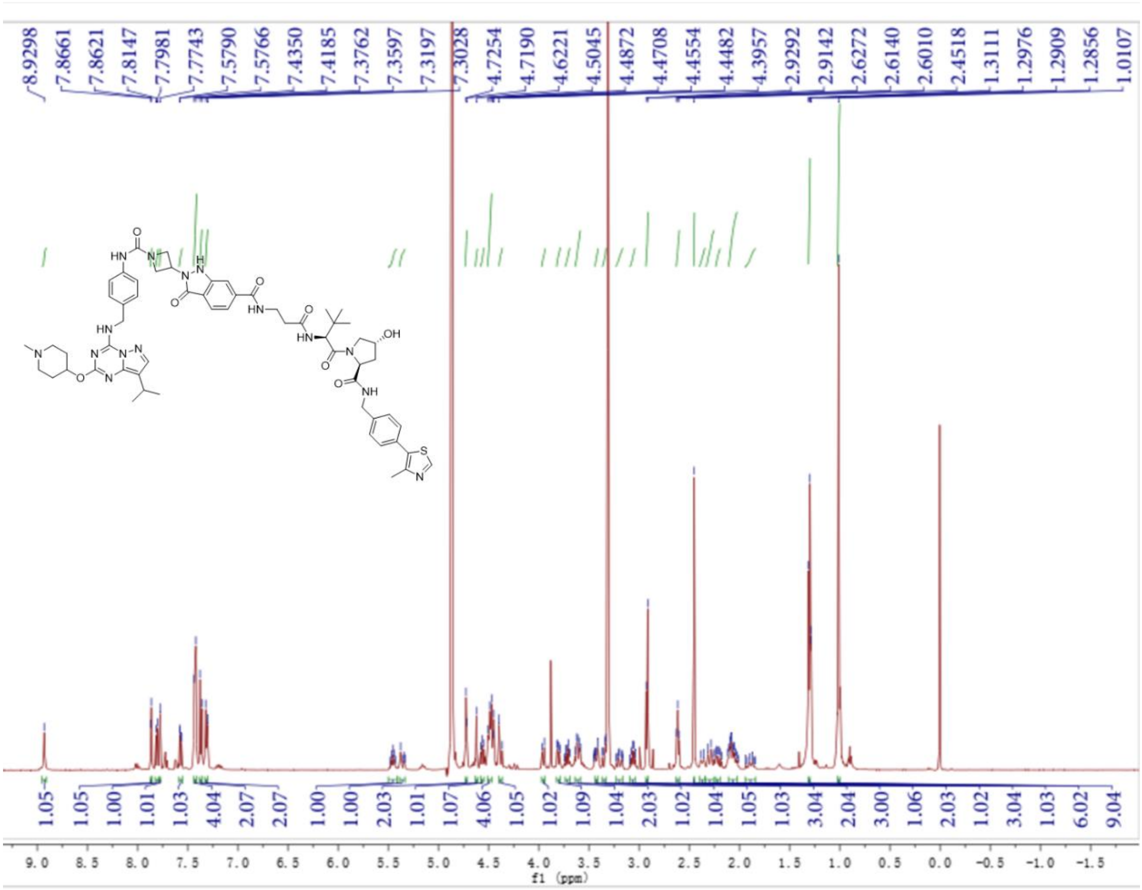
8.9298
7.8661
7.8621
7.8147
7.7981
7.7743
7.5790
7.5766
7.4350
7.4185
7.3762
7.3597
7.3197
7.3028
4.7254
4.7190
4.6221
4.5045
4.4872
4.4708
4.4554
4.4482
4.3957
2.9292
2.9142
2.6272
2.6140
2.6010
2.4518
1.3111
1.2976
1.2909
1.2856
1.0107
1.05
1.05
1.00
1.01
1.03
4.04
2.07
2.07
1.00
1.00
2.03
1.01
1.07
4.06
1.05
1.02
1.09
1.04
2.03
1.02
1.04
1.05
1.03
3.04
2.04
3.00
1.06
2.03
1.02
3.04
1.03
6.02
9.04
f1 (ppm)

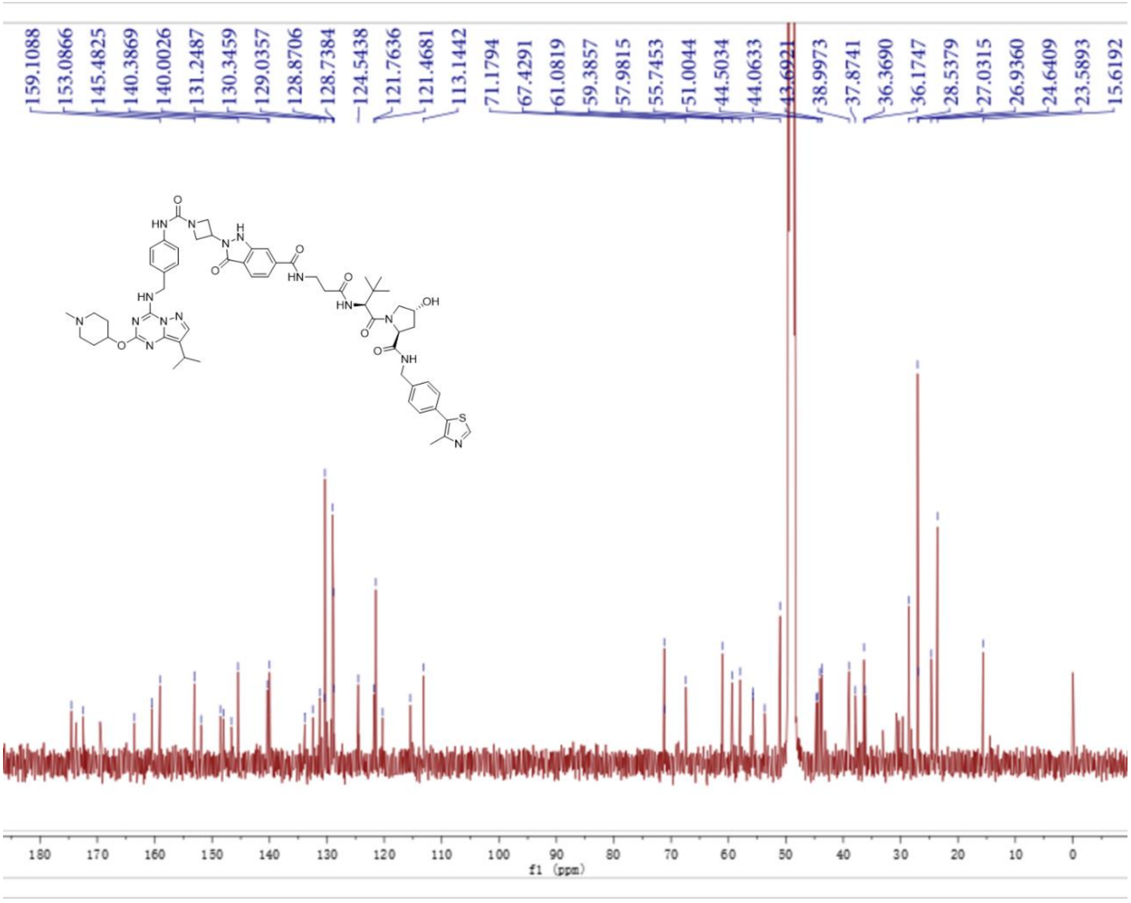
159.1088
153.0866
145.4825
140.3869
140.0026
131.2487
130.3459
129.0357
128.8706
128.7384
124.5438
121.7636
121.4681
113.1442
71.1794
67.4291
61.0819
59.3857
57.9815
55.7453
51.0044
44.5034
44.0633
43.6921
38.9973
37.8741
36.3690
36.1747
28.5379
27.0315
26.9360
24.6409
23.5893
15.6192
f1 (ppm)

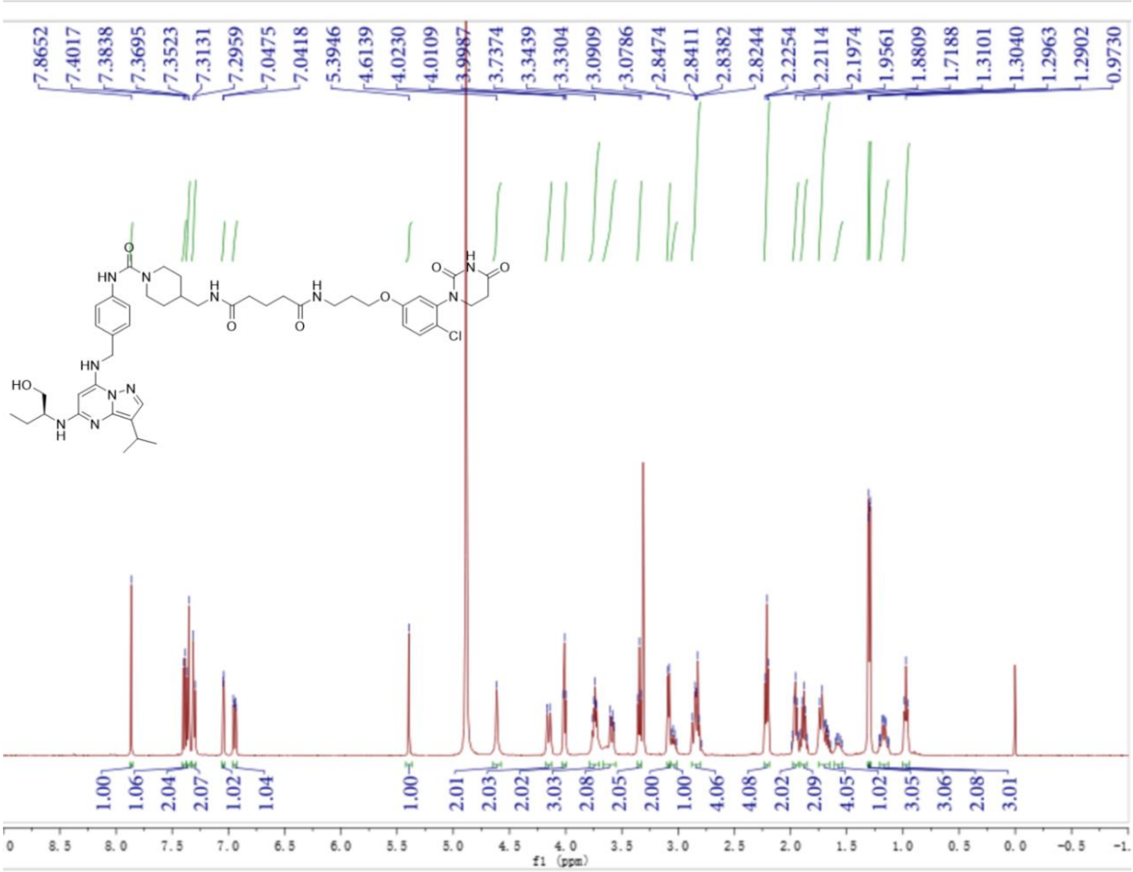
7.8652
7.4017
7.3838
7.3695
7.3523
7.3131
7.2959
7.0475
7.0418
5.3946
4.6139
4.0230
4.0109
3.9987
3.7374
3.3439
3.3304
3.0909
3.0786
2.8474
2.8411
2.8382
2.8244
2.2254
2.2114
2.1974
1.9561
1.8809
1.7188
1.3101
1.3040
1.2963
1.2902
0.9730
1.00
1.06
2.04
2.07
1.02
1.04
1.00
2.01
2.03
2.02
3.03
2.08
2.05
2.00
1.00
4.06
4.08
2.02
2.09
4.05
1.02
3.05
3.06
2.08
3.01
f1 (ppm)

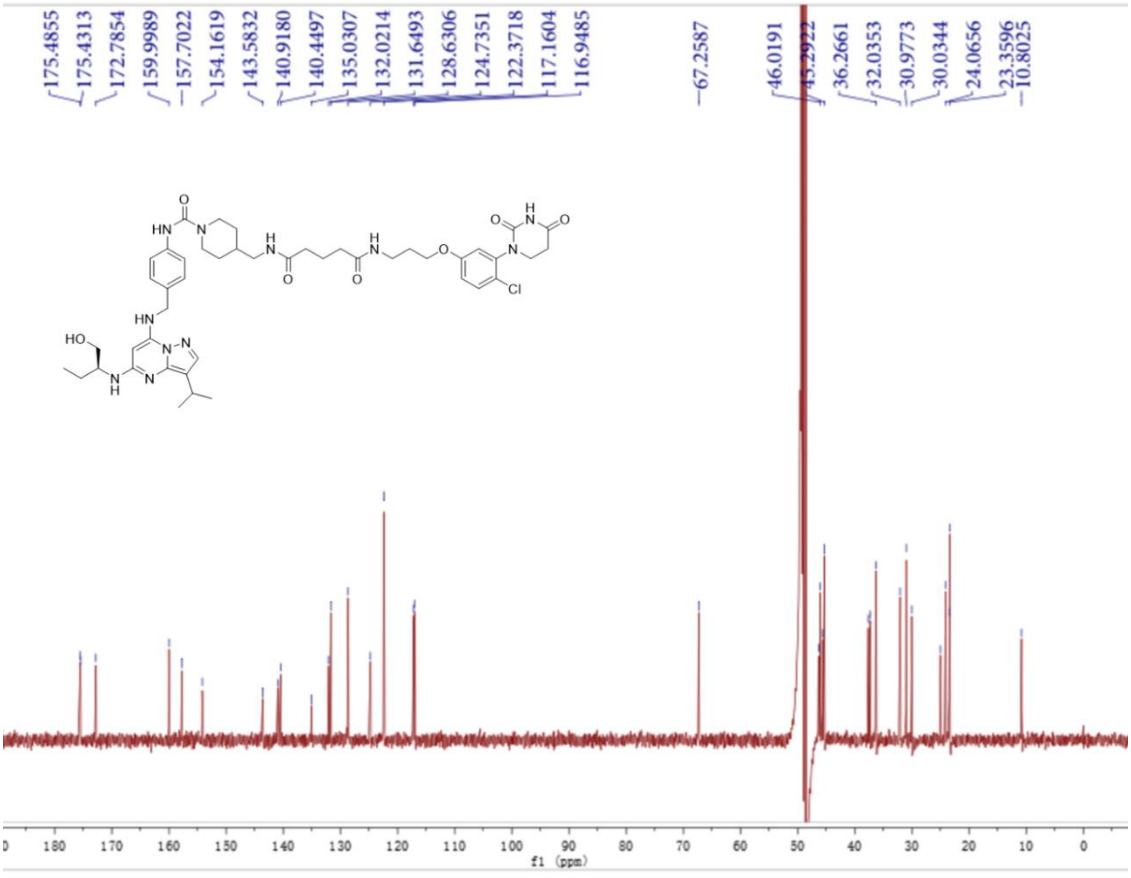
175.4855
175.4313
172.7854
159.9989
157.7022
154.1619
143.5832
140.9180
140.4497
135.0307
132.0214
131.6493
128.6306
124.7351
122.3718
117.1604
116.9485
67.2587
46.0191
45.2922
36.2661
32.0353
30.9773
30.0344
24.0656
23.3596
10.8025
f1 (ppm)

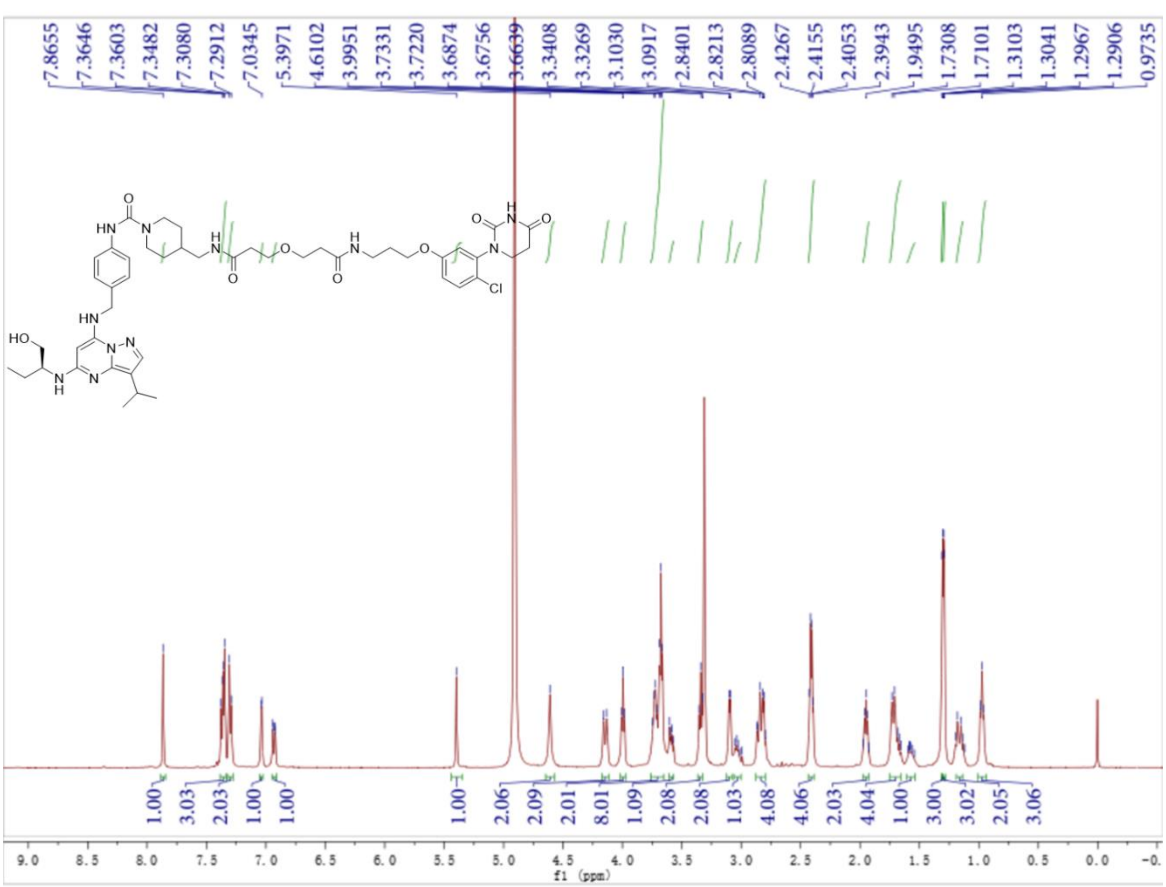
7.8655
7.3646
7.3603
7.3482
7.3080
7.2912
7.0345
5.3971
4.6102
3.9951
3.7331
3.7220
3.6874
3.6756
3.6639
3.3408
3.3269
3.1030
3.0917
2.8401
2.8213
2.8089
2.4267
2.4155
2.4053
2.3943
1.9495
1.7308
1.7101
1.3103
1.3041
1.2967
1.2906
0.9735
1.00
3.03
2.03
1.00
1.00
1.00
2.06
2.09
2.01
8.01
1.09
2.08
2.08
1.03
4.08
4.06
2.03
4.04
1.00
3.00
3.02
2.05
3.06
f1 (ppm)

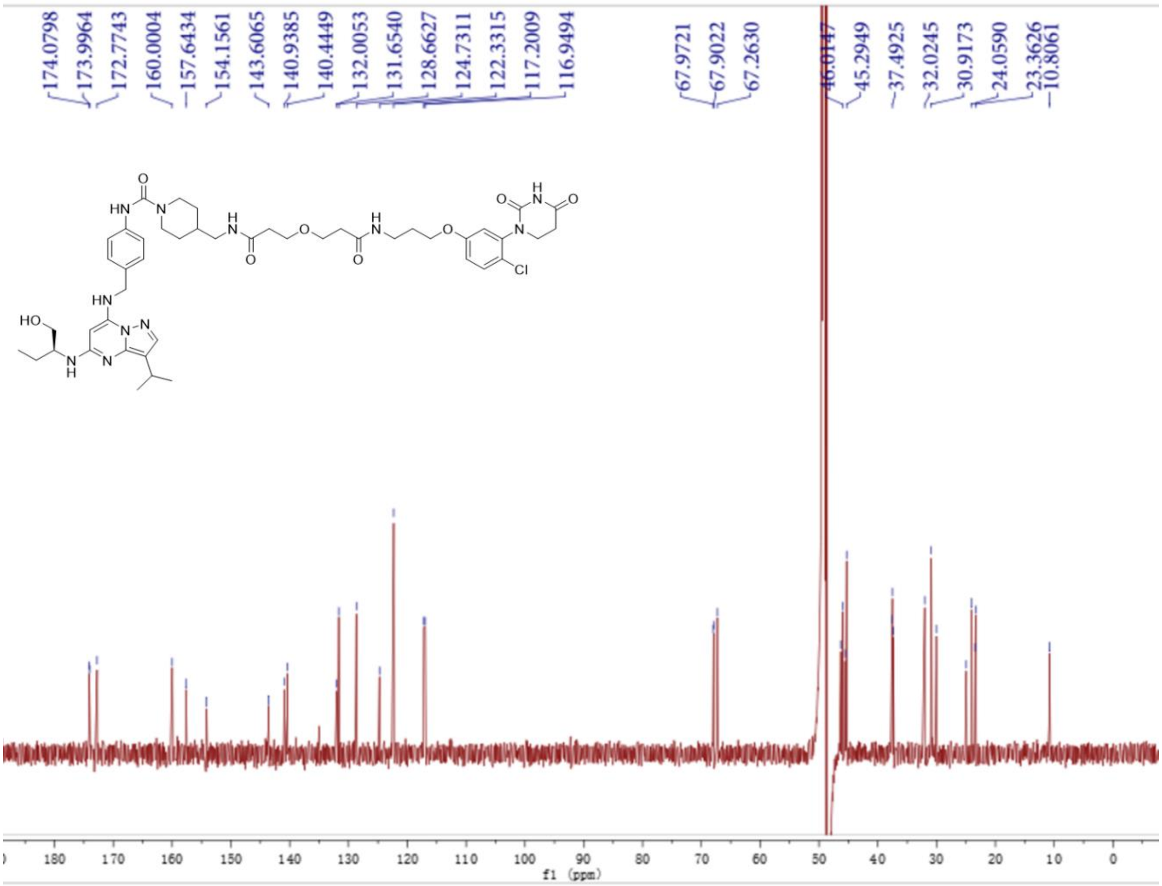
174.0798
173.9964
172.7743
160.0004
157.6434
154.1561
143.6065
140.9385
140.4449
132.0053
131.6540
128.6627
124.7311
122.3315
117.2009
116.9494
67.9721
67.9022
67.2630
46.0147
45.2949
37.4925
32.0245
30.9173
24.0590
23.3626
10.8061
f1 (ppm)

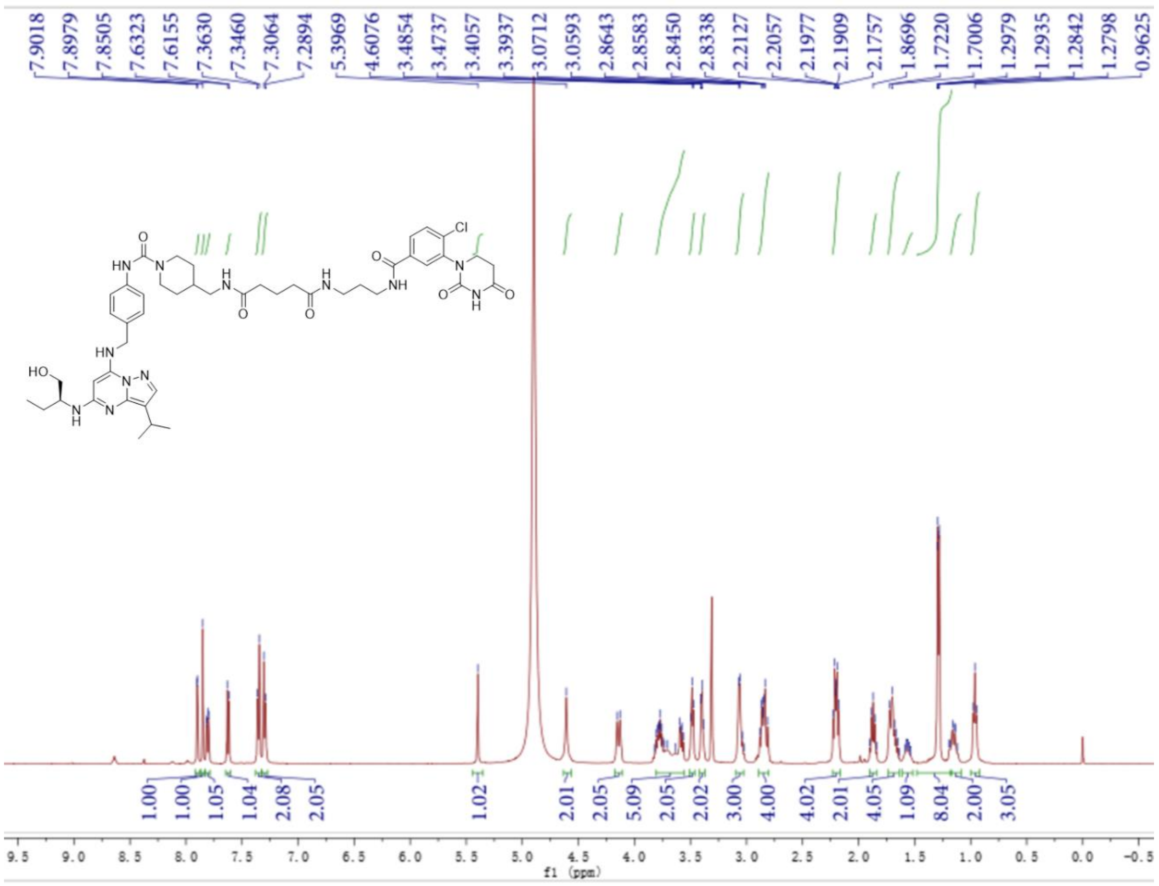
7.9018
7.8979
7.8505
7.6323
7.6155
7.3630
7.3460
7.3064
7.2894
5.3969
4.6076
3.4854
3.4737
3.4057
3.3937
3.0712
3.0593
2.8643
2.8583
2.8450
2.8338
2.2127
2.2057
2.1977
2.1909
2.1757
1.8696
1.7220
1.7006
1.2979
1.2935
1.2842
1.2798
0.9625
1.00
1.00
1.05
1.04
2.08
2.05
1.02
2.01
2.05
5.09
2.05
2.02
3.00
4.00
4.02
2.01
4.05
1.09
8.04
2.00
3.05
9.5 9.0 8.5 8.0 7.5 7.0 6.5 6.0 5.5 5.0 4.5 4.0 3.5 3.0 2.5 2.0 1.5 1.0 0.5 0.0 -0.5
f1 (ppm)

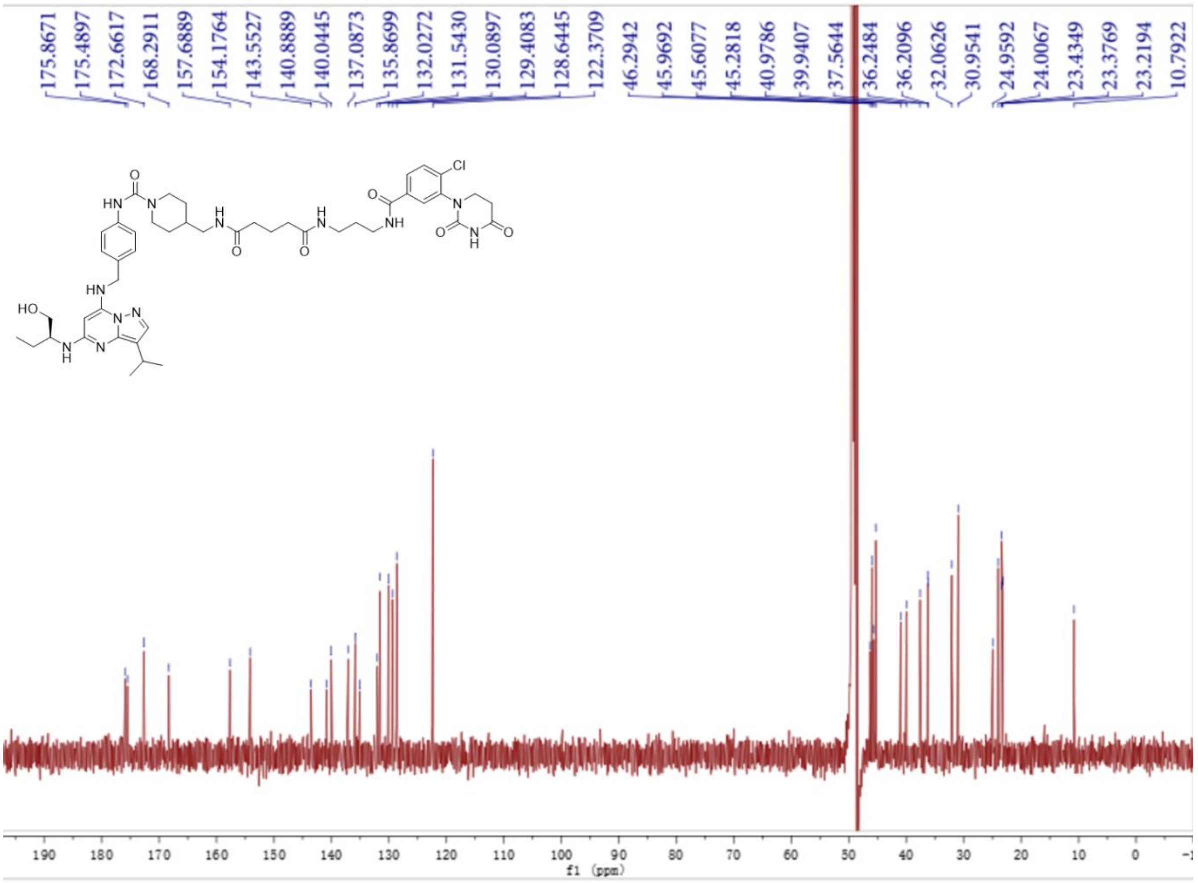
175.8671
175.4897
172.6617
168.2911
157.6889
154.1764
143.5527
140.8889
140.0445
137.0873
135.8699
132.0272
131.5430
130.0897
129.4083
128.6445
122.3709
46.2942
45.9692
45.6077
45.2818
40.9786
39.9407
37.5644
36.2484
36.2096
32.0626
30.9541
24.9592
24.0067
23.4349
23.3769
23.2194
10.7922
190 180 170 160 150 140 130 120 110 100 90 80 70 60 50 40 30 20 10 0
f1 (ppm)

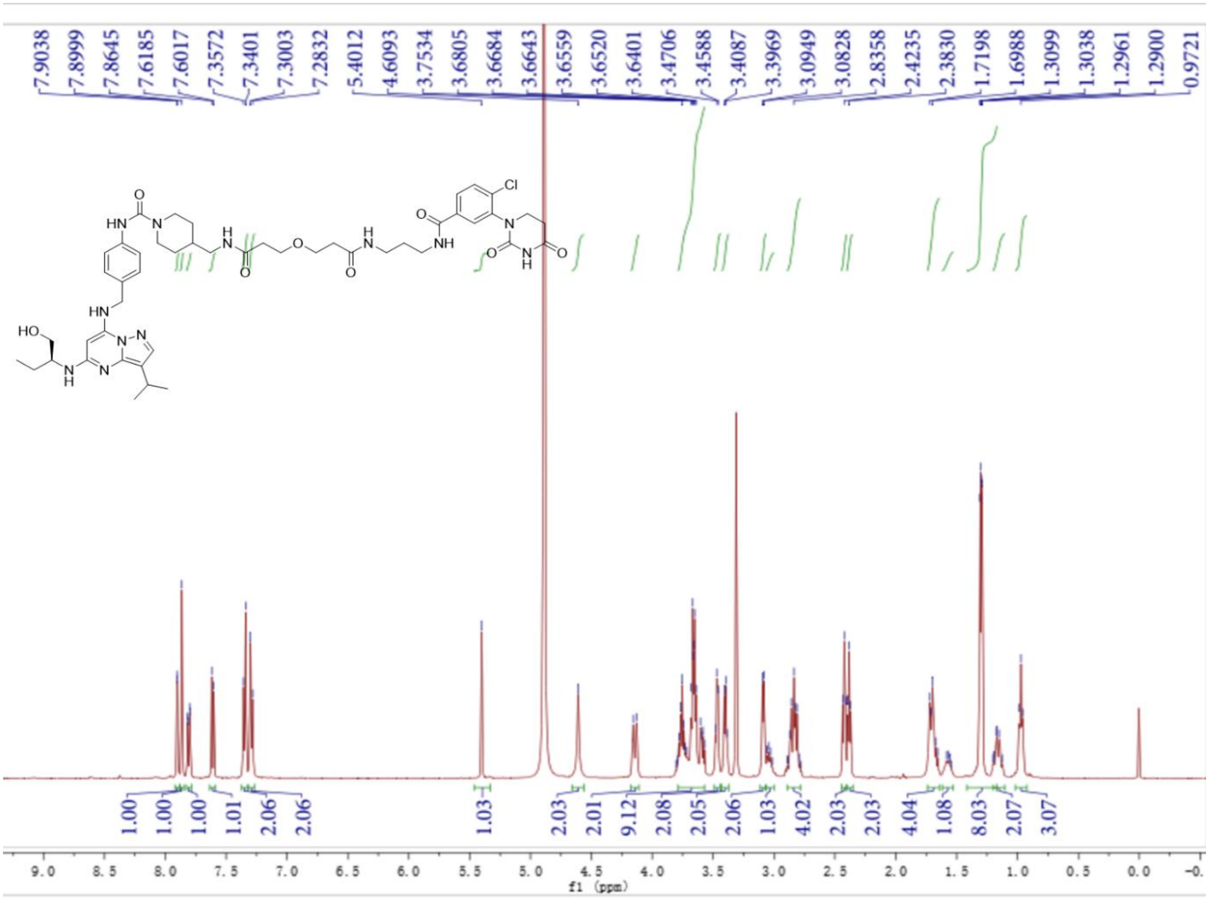
7.9038
7.8999
7.8645
7.6185
7.6017
7.3572
7.3401
7.3003
7.2832
5.4012
4.6093
3.7534
3.6805
3.6684
3.6643
3.6559
3.6520
3.6401
3.4706
3.4588
3.4087
3.3969
3.0949
3.0828
2.8358
2.4235
2.3830
1.7198
1.6988
1.3099
1.3038
1.2961
1.2900
0.9721
1.00
1.00
1.00
1.01
2.06
2.06
1.03
2.03
2.01
9.12
2.08
2.05
2.06
1.03
4.02
2.03
2.03
4.04
1.08
8.03
2.07
3.07
f1 (ppm)

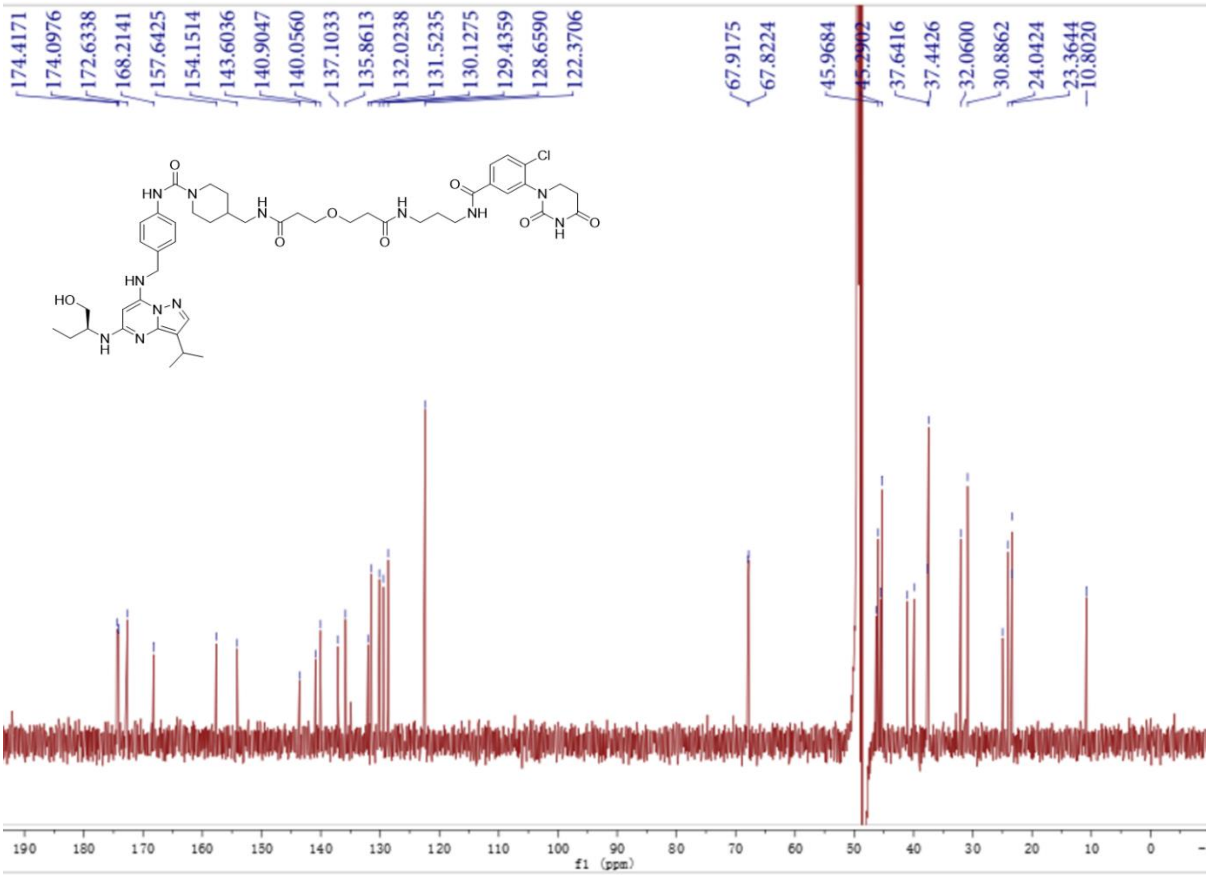
174.4171
174.0976
172.6338
168.2141
157.6425
154.1514
143.6036
140.9047
140.0560
137.1033
135.8613
132.0238
131.5235
130.1275
129.4359
128.6590
122.3706
67.9175
67.8224
45.9684
45.2902
37.6416
37.4426
32.0600
30.8862
24.0424
23.3644
10.8020
f1 (ppm)

Reference

1. Zhou, B. et al. Discovery of a Small-Molecule Degrader of Bromodomain and Extra-Terminal (BET) Proteins with Picomolar Cellular Potencies and Capable of Achieving Tumor Regression. *J. Med. Chem.* **61**, 2, 462–481 (2018).

2. Zhang, H. et al. Discovery of potent epidermal growth factor receptor (EGFR) degraders by proteolysis targeting chimera (PROTAC). *Eur. J. Med. Chem.* **189**, 112061 (2020).

3. Cromm, M. P. et al. Addressing Kinase-Independent Functions of Fak via PROTAC-Mediated Degradation. *J. Am. Chem. Soc.* **140(49)**, 17019–17026 (2018).

4. Xie, S. et al. Development of Alectinib-Based PROTACs as Novel PotentDegraders of Anaplastic Lymphoma Kinase (ALK). *J. Med. Chem.* **64**, 13, 9120–9140 (2021).

5. Matthew, N. et al. Preparation of substituted hydroxyphenylpyridazinamines for treating BAF complex-related disorders. World Intellectual Property Organization, WO2021207291.

6. Dölle A. et al. Design, Synthesis, and Evaluation of WD-Repeat-Containing Protein5 (WDR5) Degraders. *J. Med. Chem.* **64**, 15, 10682–10710 (2021).

7. Ichikawa, S. *et al.* The E3 ligase adapter cereblon targets the C-terminal cyclic imide degron. *Nature* **610**, 775–782 (2022).

8. Jaeki M. et al. Molecules and methods related to treatment of disorders associated with JAK-2 signaling dysfunction. World Intellectual Property Organization, WO2022133285.

9. Julien L. et al. Preparation of dihydropyrimidine derivatives as Brd9 bifunctional degraders for the treatment and prophylaxis of Brd9-mediated diseases. World Intellectual Property Organization, WO2021055295

10. Wei, D. et al. Discovery of potent and selective CDK9 degraders for targeting transcription regulation in triple-negative breast cancer. *J. Med. Chem.* **64**, 14822-14847 (2021).

11. Gilbert, B. et al. Preparation of pyrrolopyrimidine compounds as CDK inhibitors. World Intellectual Property Organization, WO2010020675.
